# Cationic Group 13/14/15 Element Chain Compounds with Pnictogen-Donor Ligands

**DOI:** 10.1021/acs.inorgchem.6c00556

**Published:** 2026-05-04

**Authors:** Tatiana N. Parfeniuk, Matthias T. Ackermann, Christoph Riesinger, Manfred Scheer

**Affiliations:** Institute of Inorganic Chemistry, University of Regensburg, 93053 Regensburg, Germany

## Abstract

The reactivity of IDipp·GeH_2_BH_2_OTf (**1**) (IDipp = 1,3-bis­(2,6-diisopropylphenyl)­imidazolin-2-ylidene) toward monodentate N-donor and bidentate pnictogen-donor ligands is reported. In the reaction of **1** with triethyl amine, the cationic adduct [IDipp·GeH_2_BH_2_·NEt_3_]^+^(**2** ^**+**^) is formed. In contrast, primary and secondary amines exhibit a proton transfer to the germanium center, resulting in the formation of [IDipp·GeH_3_]^+^ (**4** ^**+**^) and [IDipp·BH_2_·NHR’R’’]^+^ (**3** ^**+**^ **a–c**) species. DFT calculations reveal that the driving force for the observed difference in reactivity is the tendency of NHEt_2_ to transfer a proton to **1**, leading to the formation of **3a**, **4**, and [IDippH]^+^. Pyridine and aminopyridine also form stable adducts [IDipp·GeH_2_BH_2_·Py]^+^ (**5** ^**+**^) and [IDipp·GeH_2_BH_2_·DMAP]^+^ (**6** ^**+**^). Reactions with bidentate ligands (bipyridine and 1,2-bis­(diphenylphosphino)­ethane) lead to the formation of unprecedented dicationic chains composed of two [IDipp·GeH_2_BH_2_]^+^ units connected via the linker to form [IDipp·GeH_2_BH_2_·bipy·BH_2_GeH_2_·IDipp]^2+^ (**7** ^**2+**^) and [IDipp·GeH_2_BH_2_·dppe·BH_2_GeH_2_·IDipp]^2+^ (**8** ^**2+**^), respectively. All synthesized compounds are characterized by SC-X-ray crystallography, NMR spectroscopy, and mass spectrometry, providing insights into their structural features.

## Graphical abstract

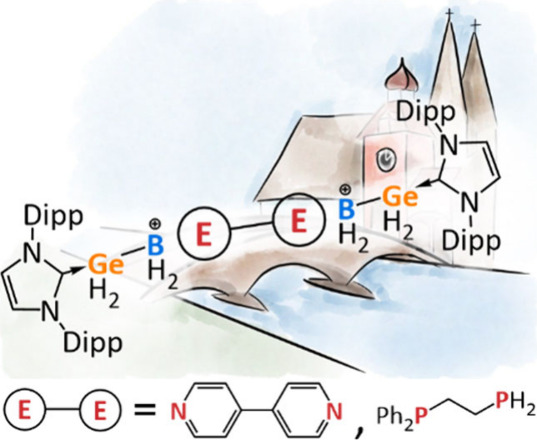

## Introduction

The synthesis of molecular compounds incorporating combinations of group 13, 14, and 15 elements has earned significant attention over the past decades due to their importance in both fundamental chemistry and materials science. Based on the isolobal analogy between the E^14^H_2_, [E^13^H_2_]^−^, and [E^15^H_2_]^+^ fragments, binary or ternary combinations of these moieties serve as inorganic analogs of hydrocarbons.^1^ Thus, such compounds can be regarded as building blocks for inorganic polymers, which are of interest as a rising class of materials with unique features.^2–8^ Furthermore, both monomeric and oligomeric compounds are prominent single-source precursors (SSPs) for important materials, such as III–V and IV–V semiconductors.^5,9–16^

Although isoelectronic with hydrocarbons, related main-group element compounds exhibit diverse reactivity due to the higher polarity of E-E’ bonds. Since the [E^13/14/15^H_2_]^−/0/+^ moiety possesses both a lone pair of electrons in its valence s-orbital, as well as an empty p-orbital, molecules with different combinations of these fragments tend to oligomerize. To prevent this oligomerization, the respective monomers require additional stabilization – either sterically or electronically. Steric protection is achieved by employing bulky organic substituents, e.g. polyalkylphenyl groups, which shield the reactive sites and prevent oligomerization and enhance the stability but disfavors a subsequent reactivity.^17–23^

A powerful strategy to suppress undesired reactivity is a donor–acceptor stabilization, for instance with electron-rich N-heterocyclic carbenes (NHCs). The donor–acceptor approach has enabled the stabilization of the low valent tetrylenes, where the ER_2_ (E = Si, Ge, Sn) fragment simultaneously possesses a lone pair of electrons and an empty p-orbital, thereby exhibiting ambiphilic behavior.^24–28^ NHC-stabilized tetrylenes have become promising building blocks for introducing Si or Ge into mixed-element molecules, and their further functionalization has been excessively studied.^29–34^ Notably, Rivard and co-workers demonstrated this donor–acceptor strategy by isolating a Ge­(II) dihydride complex stabilized by a NHC and a borane as Lewis acid, followed by the first stable Si­(II) dihydride analogue (Scheme 1a).^28,35,36^ Moreover, donor–acceptor stabilization has proven essential for isolating binary main-group compounds such as R_2_E^13^–E^15^R’_2_ and for achieving control over their polymerizations and oligomerizations.^37–41^ Significant contributions for the stabilization of parent H_2_E^13^–E^15^H_2_ compounds were made by our group, including the synthesis of combinations involving heavier group 13 and 15 elements (Scheme 1a).^42–50^ In contrast to binary main-group element combinations, ternary compounds containing elements from three different groups are much rarer and mainly represented by cyclic compounds or species containing cyclic fragments. Early examples include cluster or cage compounds with combinations such as B–Ge–P or B–Sn–P.^51–53^ More recently, cyclic frameworks featuring three different main-group elements have been isolated.^54–64^ Considering linear heteroatomic chains derived via insertion or addition reactions, the number of known examples is even smaller (Scheme 1b).^17,65–79^ For instance, some of the earlier examples from the groups of Tokitoh, Pain and Nöth involved lighter group 13 and 14 elements such as boron and silicon (**I–II**). Later, chain compounds with heavier congeners, including gallium and tin, were achieved by the groups of von Hänisch (**IV**) and Wright (**V**).^74,75^ This scarcity highlights the challenge and significance of assembling three different main-group element types in a controlled linear arrangement and show the need of organic substituents for its stabilization. Recently in a collaboration between the Rivard and our group, the synthesis of the cationic precursor [IDipp·GeH_2_BH_2_]^+^ was achieved. This compound has proven its utility as a building block for group 13/14/15 mixed-element chains with P and As donors, respectively.^80–82^ Therefore, the question arises as to whether this reactivity could be expanded to N-donors and even bidentate ligands, potentially leading to the formation of unique cationic chain compounds (Scheme 1c). Herein, we report on the expansion of the reactivity of [IDipp·GeH_2_BH_2_]^+^ toward monodentate N-donor and bidentate pnictogen-donor ligands to obtain novel group 13/14/15 compounds possessing parent group 13/14 parts.

**Scheme 1:**
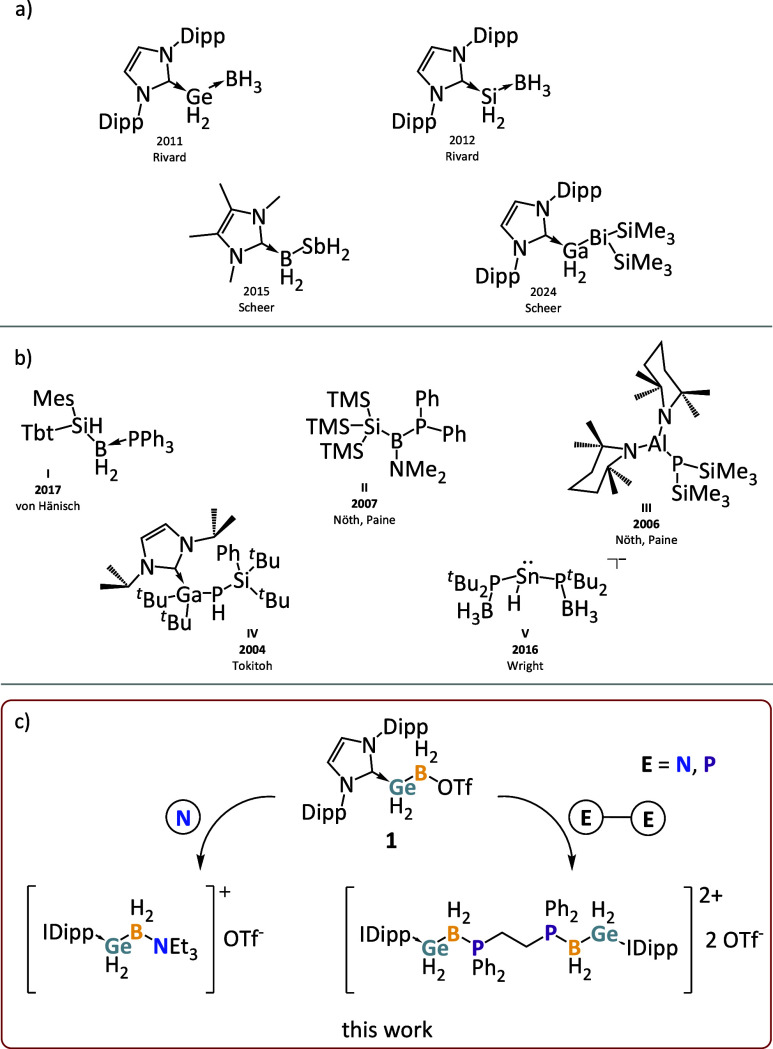
^a^

a ((a) Examples of donor–acceptor stabilized low valent groups 13/14 and 13/15 element compounds; (b) selected examples of linear mixed group 13/14/15 elements compounds; (c) functionalization pathways of IDipp·GeH_2_BH_2_OTf (formal charges are omitted but the positive charge is located on the germanium atom). Mes = 2,4,6-trimethylphenyl, Tbt = 2,4,6-tris­[bis­(trimethylsilyl)­methyl]­phenyl, TMS = trimethylsilyl, Dipp = 2,6-diisopropylphenyl.

## Results and Discussion

### Reactivity of **1** toward Amines

The reactivity of IDipp·GeH_2_BH_2_OTf (**1**) toward amines was investigated, starting with the tertiary amine NEt_3_ (Scheme 2). The reaction proceeds in Et_2_O at room temperature to give [IDipp·GeH_2_BH_2_·NEt_3_]­[OTf] (**2**) in excellent yield (86%). In the crystal structure of **2** (Figure 1), the B1–N1 bond length of 1.609(2) Å is slightly longer than the B–N bond in ammonia borane H_3_N→BH_3_ (1.56(5) Å).^83^ The Ge–B bond amounts to 2.0734(18) Å and is slightly longer than that in the neutral compound IDipp·GeH_2_BH_3_ [2.053(3) Å], but nearly identical to the corresponding bond in **1** [2.081(3) Å].^28,80^ Notably, no short contacts between the cation and the triflate anion are observed. In contrast to the adduct formation with tertiary NEt_3_, the reaction of **1** with secondary (NHEt_2_) and primary (NH_2_^*i*^Pr, NH_2_^*t*^Bu) amines affords complicated product mixtures, containing [IDipp·BH_2_·NHEt_2_]­[OTf] (**3a**), [IDipp·BH_2_·NH_2_^*i*^Pr]­[OTf] (**3b**) and [IDipp·BH_2_·NH_2_^*t*^Bu]­[OTf] (**3c**) respectively, as well as [IDipp·GeH_3_]­[OTf] (**4**) and [IDippH]­[OTf] (Scheme 2). This type of reactivity was observed for different stoichiometric ratios of **1** to amine (1:1 and 1:2), with **3a**–**3c** being the main products in each case. However, due to severe overlap of the ^1^H NMR signals of **3**, **4** and [IDippH]­[OTf], as well as the presence of other unidentified side products, it was not possible to doubtlessly determine the ratio of products. Single crystals of compounds **3a**–**3c** suitable for XRD analysis were picked from the mixture of crystals with **4** and [IDippH]­[OTf]. In the crystal structures of **3a**–**3c**, hydrogen bonding between the amine proton and the oxygen atom of the triflate anion was observed. The C–B bonds in **3a** (1.629(5) Å), **3b** (1.603(2) Å) and **3c** (1.612(2) Å) are significantly elongated, compared to a neutral adduct IDipp·BH_3_ (1.585(4) Å) due to the σ-donation from amines, that reduces the Lewis acidity of boron center and weakens the NHC→B donation.^84^ **3a** possesses the longest bond, reflecting the stronger σ-donation from the NHEt_2_, **3b** and **3c** have shorter bonds due to weaker σ-donation of NH_2_^*i*^Pr and NH_2_^*t*^Bu, however the C–B bond in **3c** is slightly longer, due to steric repulsion. The B1–N1 bond lengths amount to 1.604(5) Å, 1.604(2) Å, and 1.6074(18) Å for **3a**, **3b** and **3c**, respectively. They are shorter than the B–N bond in **2**. Based on the identified products of this reaction, it can be proposed that the interaction of **1** with secondary and primary amines leads to protonation of the “GeH_2_” moiety and elimination of [IDipp·GeH_3_]­[OTf]. Due to the interaction of the triflate anion with amine protons, it was assumed that the triflate anion may play a decisive role in the protonation process of the germanium center. To test this, the counterion in **1** was exchanged with [BAr^F^]^−^ or [TEF]^−^ (BAr^F^ = [B­(C_6_F_5_)_4_]^−^, TEF = [Al­(OC­(CF_3_)_3_)_4_]^−^) by addition of KBAr^F^ or LiTEF in diethyl ether, monitored by NMR spectroscopy and mass spectrometry (cf. Supporting Information). Subsequently, one equivalent of NH^*i*^Pr_2_ was added to this mixture. However, according to the ESI mass spectrum of the reaction mixture, no amine complex but the formation of the etherate [IDipp·GeH_2_BH_2_·OEt_2_]^+^ was observed. This suggests that the interaction between the triflate anion and the cationic counterpart may play a crucial role in the stabilization of the main-group element chain. When the counterion is exchanged by weakly coordinating anions [BAr^F^]^−^ or [TEF]^−^, an Et_2_O molecule coordinates to the boron atom, forming a very stable adduct [IDipp·GeH_2_BH_2_·OEt_2_]^+^, which was detected by mass spectrometry. Attempts to carry out the anion exchange in 1,2-difluorobenzene, in order to avoid solvent coordination, resulted only in decomposition: in the ^1^H NMR spectra of the reaction mixtures with both [BAr^F^]^−^ and [TEF]^−^, mostly the signals of **4** were observed, and in the ^11^B NMR spectra no signals attributable to a BH_2_ moiety could be detected (cf. Supporting Information).

**Scheme 2:**
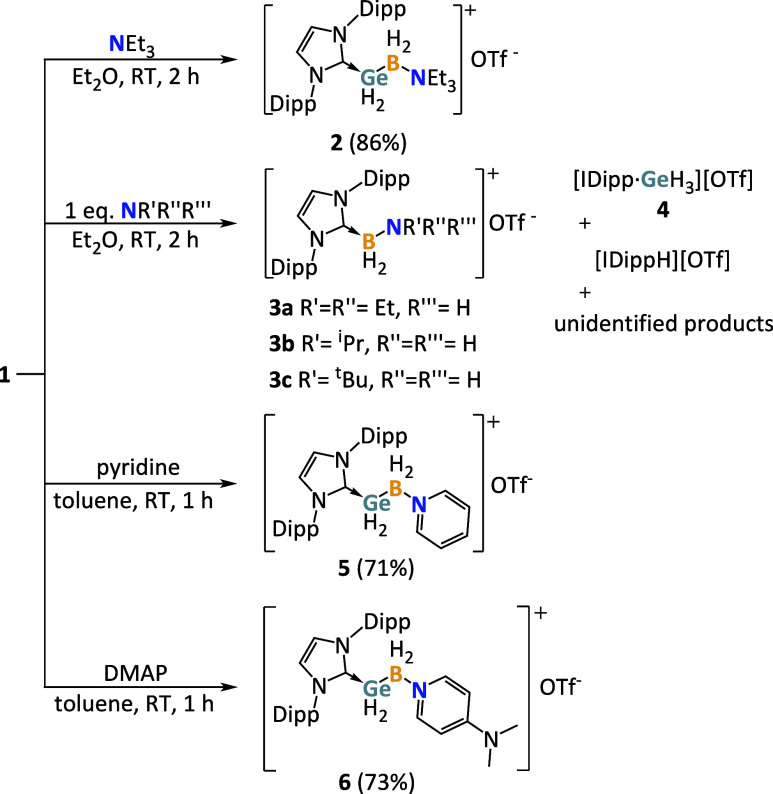
Synthesis of **2**, **3a**–**b**, **5**, **6** by the Reaction of IDipp·GeH_2_BH_2_OTf (**1**)­[Fn sch2-fn1]

A Isolated yields are given in parentheses. Formal charges are omitted but the positive charge is located on the germanium atom.

**Figure 1:**
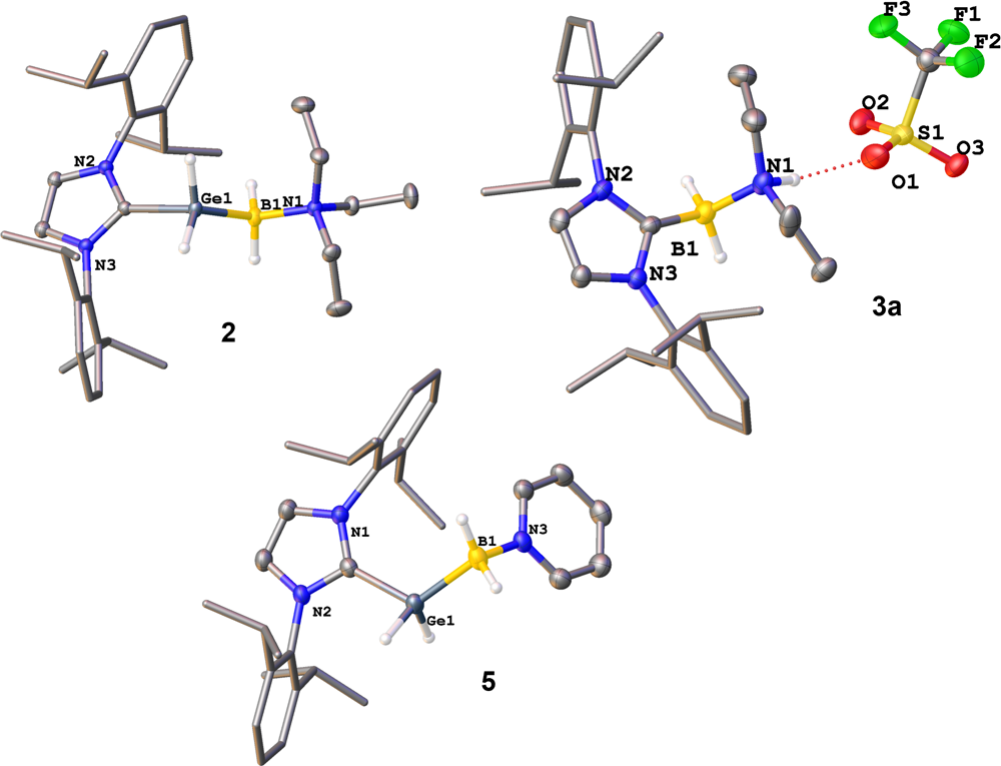
Molecular structures of the cations of **2** (top left) and **5** (bottom), respectively, and molecular structure of **3a** (top right) in the solid state with anisotropic displacement ellipsoids at the 50% probability level. Counterions and hydrogen atoms bound to carbon are omitted for clarity.

To understand the difference in reactivity of **1** with tertiary (NEt_3_) and secondary (NHEt_2_) amines, DFT calculations were performed at the B3LYP/def2-TZVP level of theory (Table 1). As products of the reaction of **1** with NHEt_2_ the cationic compounds [IDipp·BH_2_·NHEt_2_]^+^ (**3a**), [IDipp·GeH_3_]^+^ (**4**) and [IDippH]^+^ were detected by NMR spectroscopy and mass spectrometry. However, not all of the products could be identified, and therefore the complete reaction pathway could not be proposed. Thus, several potential processes leading to the formation of the cations [IDipp·BH_2_·NHEt_2_]^+^ (**3a**), [IDipp·GeH_3_]^+^ (**4**) and [IDippH]^+^ were investigated. (Table 1). The latter imidazolium salt might arise from protonation of **1** by NHEt_2_, while a formal “GeH_2_” moiety may be generated during the process, giving [IDipp·GeH_3_]^+^ (**4**). According to the computations, the formation of the expected products [IDipp·GeH_2_BH_2_·(amine)]^+^ with both NEt_3_ and NHEt_2_ is energetically favorable (Table 1, reactions 1 and 4, respectively). However, in the case of NEt_3_ the formation of a [IDipp·BH_2_·(amine)]^+^ species and the “GeH_2_” moiety is endergonic (Table 1, reaction 2), whereas for NHEt_2_ the same process is exergonic (Table 1, reaction 5). Formation of [IDipp·GeH_3_]^+^ (**4**) in the reaction of **1** with [IDippH]^+^ and NEt_3_ or NHEt_2_ (Table 1, reactions 3 and 6, respectively) is also thermodynamically favorable. In case of the secondary amine NHEt_2_, there is another type of reactivity possible: protonation of **1** by NHEt_2_, with formation of [IDipp·GeH_3_]^+^ (**4**) and the aminoborane BH_2_NEt_2_ (Table 1, reaction 7). This process is also highly energetically favorable. Thereby, out of three possible reactivity pathways proposed for the NEt_3_ reaction (Table 1, reactions 1–3), the most exergonic is the reaction 1, leading to the formation of [IDipp·GeH_2_BH_2_·NEt_3_]­[OTf] (**2**). On the contrary, for NHEt_2_ the most exergonic process out of reactions 4–7 (Table 1), is the formation of [IDipp·GeH_3_]^+^ (**4**) and the aminoborane BH_2_NEt_2_ (Table 1, reaction 7). Notably, the aminoborane can further oligomerize, acting as an additional driving force for this side reaction. This distinct reactivity pattern agrees with the experimental observations.

**Table 1:** Thermodynamic characteristics for Different Gas Phase Processes^a^

Process	Δ*H*_298_^°^	Δ*S*_298_^°^	Δ*G*_298_^°^
1 $1+{\mathrm{NEt}}_{3}=2$	–158	–221	–92
$1+{\mathrm{NEt}}_{3}={\left[\mathrm{IDipp}\cdot {\mathrm{BH}}_{2}\cdot {\mathrm{NEt}}_{3}\right]}^{+}+{\mathrm{GeH}}_{2}$ 2	–2	–91	25
$1+{\mathrm{NEt}}_{3}+{\left[\mathrm{IDippH}\right]}^{+}={\left[\mathrm{IDipp}\cdot {\mathrm{BH}}_{2}\cdot {\mathrm{NEt}}_{3}\right]}^{+}+4$ 3	–128	–280	–45
$1+{\mathrm{NHEt}}_{2}={\left[\mathrm{IDipp}\cdot {\mathrm{GeH}}_{2}{\mathrm{BH}}_{2}\cdot {\mathrm{NHEt}}_{2}\right]}^{+}$ 4	–165	–201	–106
5 $1+{\mathrm{NHEt}}_{2}=3a+{\mathrm{GeH}}_{2}$	–36	–58	–19
6 $1+{\mathrm{NHEt}}_{2}+{\left[\mathrm{IDippH}\right]}^{+}=3a+4$	–162	–246	–89
7 $1+{\mathrm{NHEt}}_{2}=4+{\mathrm{BH}}_{2}{\mathrm{NEt}}_{2}$	–154	–20	–148

a Standard reaction enthalpies Δ*H*_298_^°^ and Gibbs energies Δ*G*_298_^°^ in kJ mol^–1^, standard reactions entropies Δ*S*_298_^°^ in J mol^–1^ K^–1^. B3LYP/def2-TZVP level of theory.

### Reactivity of **1** toward Pyridine-Based Compounds

Since the amine proton appeared to be responsible for the elimination of [IDipp·GeH_3_]­[OTf], the reactivity of IDipp·GeH_2_BH_2_OTf (**1**) toward pyridine-based compounds was investigated (Scheme 2). The successful formation of the pyridine (Py) adduct [IDipp·GeH_2_BH_2_·Py]­[OTf] (**5**) was confirmed both by NMR spectroscopy and SC-XRD (Figure 2). Single crystals suitable for X-ray analysis were obtained by layering a concentrated DCM solution of the product with a 3-fold excess of *n*-hexane and storage at +9 °C. For **5**, the characteristic ^1^H NMR signals of Py were observed in the aromatic region at δ = 7.43, 7.83, and 7.94 ppm. The formation of the DMAP adduct [IDipp·GeH_2_BH_2_·DMAP]­[OTf] (**6**) was likewise confirmed by NMR spectroscopy, which showed characteristic signals for the methyl groups and the pyridine ring of DMAP in the ^1^H NMR spectrum at δ = 3.04, 6.38, and 7.35 ppm, respectively. In the crystal structure of **5**, the B1–N1 distance amounts to 1.574(2) Å, which is significantly shorter than that in the triethylamine complex **2**.

**Figure 2:**
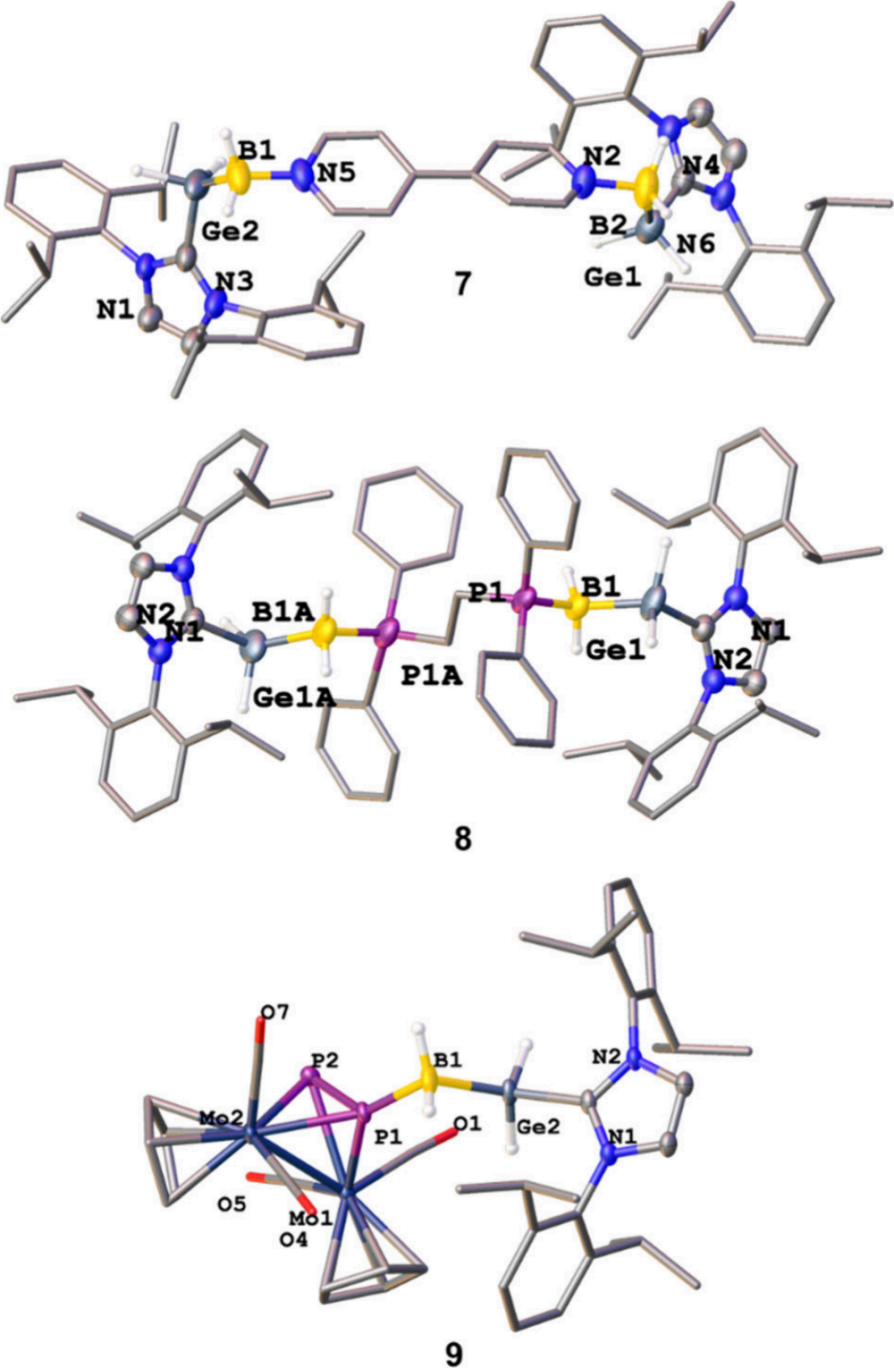
Molecular structures of the dications of **7**, **8**, and **9** in the solid state with anisotropic displacement ellipsoids at a 50% probability level. Counterions and hydrogen atoms bound to carbon are omitted for clarity.

### Reactivity of **1** toward Bidentate Linkers

Furthermore, the possibility of forming dicationic chains by the reaction of the precursor IDipp·GeH_2_BH_2_OTf (**1**) with bidentate linkers was examined. First, the reactivity toward TMEDA (tetramethylethylenediamine) was studied. However, as evident from the ^11^B NMR spectrum (cf. Supporting Information), TMEDA abstracts the “[BH_2_]^+^” moiety from the chain by chelation, giving the product [TMEDA·BH_2_]^+^, which exhibits a characteristic signal at δ = 6.2 ppm.^85^ The cation [IDipp·GeH_3_]^+^ is formed as a byproduct of this reaction. Since TMEDA, as a flexible linker, is able to cleave the Ge–B bond, rigid linkers were then employed in order to access dicationic chain compounds. The reaction of two equivalents of IDipp·GeH_2_BH_2_OTf with 4,4'-bipyridine (bipy) in toluene at room temperature led to the 2:1 complex [IDipp·GeH_2_BH_2_·bipy·BH_2_GeH_2_·IDipp]­[OTf]_2_ (**7**) (Scheme 3), which was characterized by NMR spectroscopy, SC-XRD and mass spectrometry. In the ^1^H NMR spectrum of **7**, the characteristic doublet signals of bipy appear at δ = 7.96 ppm and δ = 8.00 ppm, respectively. The integrals of the proton signals also confirm the 2:1 ratio of IDipp to bipy. The B1–N5 and B2–N2 bond lengths in **7** (Figure 2) are the same at 1.585(4) Å, despite the absence of crystallographic symmetry, a value nearly identical to the corresponding bond length in the Py adduct **5**. Reaction of IDipp·GeH_2_BH_2_OTf with 1,2-bis­(diphenylphosphino)­ethane (dppe) in toluene at room temperature afforded the 2:1 complex [IDipp·GeH_2_BH_2_·dppe·BH_2_GeH_2_·IDipp]­[OTf]_2_ (**8**) (Scheme 3). In the crystal structure of **8** (Figure 2) the two P–B bonds with distances of 1.891(18) Å and 1.948(19) Å, respectively, differ slightly. However, considering the relatively large standard deviations arising from the pronounced disorder in the central chain fragment, this difference should be interpreted with caution. Moreover, the ^31^P NMR spectrum at room temperature exhibits only one broad signal at δ = 19.2 ppm (ω_1/2_ = 90 Hz), which could not be resolved even at −80 °C (ω_1/2_ = 74 Hz), indicating that both phosphorus atoms are chemically equivalent. Interestingly, the ^1^H NMR spectrum of **7** containing the bipy linker shows a set of two doublets for the methine protons of the isopropyl groups of the IDipp carbene, whereas the dppe complex **8** gives only one doublet. This difference can be attributed to the free rotation along the dppe backbone.

**Scheme 3:**
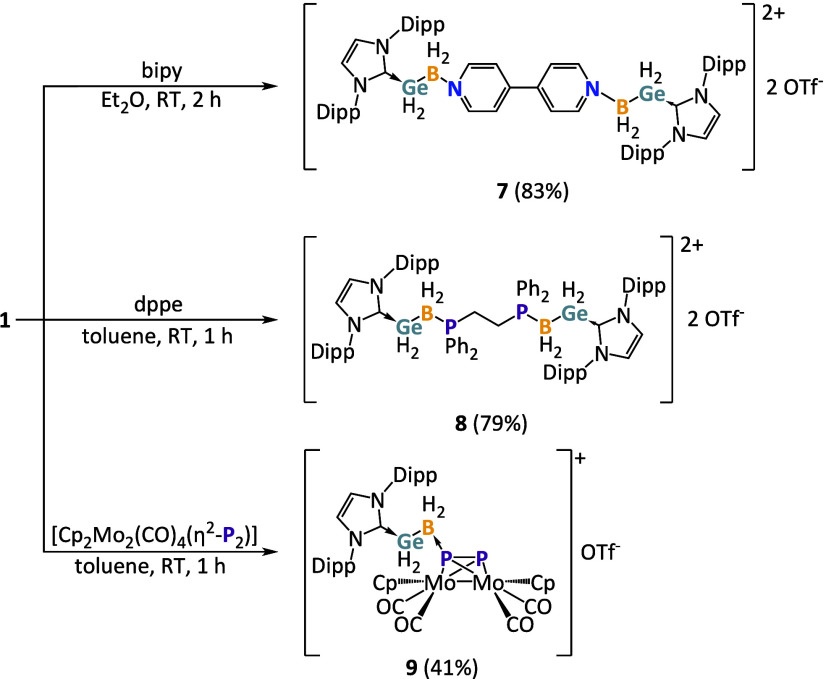
Synthesis of **7**, **8**, and **9** by the Reaction of IDipp·GeH_2_BH_2_OTf (**1**) with Different Bidentate Linkers^a^

a Isolated yields are given in parentheses. Formal charges are omitted but the positive charge is located on the germanium atom.

Other potential phosphorus-centered bidentate linkers were also tested in reactions with IDipp·GeH_2_BH_2_OTf (**1**). The tetrahedral complex [Cp_2_Mo_2_(CO)_4_(η^2^-P_2_)][Bibr ref86] was reacted with IDipp•GeH_2_BH_2_OTf in both 1:2 and 1:1 stoichiometries. However, in both cases only the one-fold addition product [Cp_2_Mo_2_(CO)_4_(η^2^-P_2_)·BH_2_GeH_2_·IDipp]­[OTf] (**9**) was obtained (Scheme 3). The ^31^P NMR spectrum of **9** shows two doublets at δ = −18.5 ppm and −158.3 ppm corresponding to two inequivalent P atoms, coupled to each other with ^1^ *J*_*P,P*_ = 505 Hz. The single crystal X-ray structure analysis of **9** also confirms the formation of the 1:1 complex. Two molecules of **9** crystallize in the asymmetric unit, exhibiting the B–P bond lengths of 1.953(3) Å and 1.949(3) Å, respectively, which are equal within an experimental error. Those values are similar to the B–P bond lengths in **8**. However the P-P bonds of the distances 2.0725(8) Å and 2.0742(8) Å in **9** are slightly shorter than in the parent compound [Cp_2_Mo_2_(CO)_4_(η^2^-P_2_)] 2.079(2) Å, indicating a polarization of the P–P bond.^86^ The moderate nucleophilicity of [Cp_2_Mo_2_(CO)_4_(η^2^-P_2_)] may explain why the reaction with a second equivalent does not proceed. The reaction of **1** with white phosphorus was studied subsequently. However, no reaction was observed, even after prolonged reflux in THF, consistent with the fact that white phosphorus is a very weak nucleophile. The tetrahedral arsenic complex [Cp*_2_Mo_2_(CO)_4_(η^2^-As_2_)][Bibr ref87] was also treated with two equivalents of **1**, but only the formation of [IDipp·GeH_3_]­[OTf] as a decomposition product was observed. The nucleophilicity of such tetrahedral pnictogen complexes decreases down the group, and the energy of the lone pairs at the arsenic atoms was shown to be relatively low.^88,89^ Therefore, the diarsenic compound is not reactive enough to bind **1**.

## Conclusion

The broad synthetic versatility of the cationic precursor [IDipp·GeH_2_BH_2_]^+^ has been clearly demonstrated through its ability to access a wide range of novel structurally diverse group 13/14/15 element chain compounds. Its rich and tunable reactivity toward various pnictogen-donor ligands enabled the isolation of several new, well-defined mono- and dicationic complexes, highlighting its utility as a modular building block for unprecedented mixed main-group systems. Reactions with tertiary amines, pyridine, and aminopyridine proceeded cleanly to afford the stable monocationic adducts [IDipp·GeH_2_BH_2_·NEt_3_]­[OTf] (**2**), [IDipp·GeH_2_BH_2_·Py]­[OTf] (**5**) and [IDipp·GeH_2_BH_2_·DMAP]­[OTf] (**6**) in which the donor ligand coordinates to the boron center. In contrast, secondary and primary amines exhibit fundamentally different reactivity toward [IDipp·GeH_2_BH_2_]^+^, readily inducing proton transfer to the germanium center. This process results in the formation of [IDipp·GeH_3_]^+^ (**4**) and aminoborane-derived cations [IDipp·BH_2_·NHR’R’’]^+^ (**3a**–**3b**), underscoring the critical influence of the amine proton on the reaction outcome. Density functional theory (DFT) calculations were performed to rationalize these experimentally observed trends and provided valuable insight into the energetic preference for adduct formation versus proton transfer pathways. Furthermore, the use of bidentate linkers, such as rigid 4,4'-bipyridine (bipy) and flexible 1,2-bis­(diphenylphosphino)­ethane (dppe), enabled the assembly of unprecedented dicationic chain compounds [IDipp·GeH_2_BH_2_·bipy·BH_2_GeH_2_·IDipp]­[OTf]_2_ (**7**) and [IDipp·GeH_2_BH_2_·dppe·BH_2_GeH_2_·IDipp]­[OTf]_2_ (**8**). These species feature two [IDipp·GeH_2_BH_2_]^+^ units bridged by a bidentate ligand, representing a novel structural motif within mixed group 13/14/15 element chemistry. In addition, the reactivity of **1** toward the tetrahedral pnictogen complex [Cp_2_Mo_2_(CO)_4_(η^2^-P_2_)] was explored, resulting exclusively in the formation of the 1:1 adduct [Cp_2_Mo_2_(CO)_4_(η^2^-P_2_)·BH_2_GeH_2_·IDipp]­[OTf] (**9**). All isolated compounds were comprehensively characterized by multinuclear NMR spectroscopy, single-crystal X-ray diffraction, and mass spectrometry, providing detailed insight into the bonding environments and structural motifs of these novel main-group element chain compounds.

## Experimental Section

### Working Techniques and Materials

All the manipulations were carried in the inert atmosphere of dry argon using the glovebox or Schlenk techniques. The argon gas was purified from traces of H_2_O and O_2_ by passing it through the BASF R 3–11 (CuO/MgSiO_3_) catalyst, concentrated H_2_SO_4_, and then Orange Gel and Sicapent supported on pumice stone. Et_2_O, CH_2_Cl_2_, toluene, hexane and pentane were purified using an MBraun SPS-800 solvent purification system, degassed at room temperature, and stored over molecular sieves for at least 48 h. Deuterated solvents and o-DFB were dried over CaH_2_, distilled under argon and stored over the molecular sieves. The NMR spectra were recorded on a Bruker Avance 400 spectrometer (^1^H: 400.13 MHz, ^31^P: 161.976 MHz, ^11^B: 128.378 MHz, ^19^F: 376.498 MHz) or Avance 500 (^1^H: 500.178 MHz; ^31^P: 202.476 MHz, ^11^B: 160.477 MHz, ^19^F: 470.637 MHz) with δ [ppm] referenced to external SiMe_4_ (^1^H), H_3_PO_4_ (^31^P), F_3_B·Et_2_O (^11^B). Mass spectra were recorded on an Agilent Q-TOF 6540 UHD (ESI-MS) and a Jeol AccuTOF GCX spectrometer (LIFDI-MS). Elemental analyses were determined with a Vario Micro Cube apparatus. The compounds IDipp·GeH_2_BH_2_OTf^80^ and Cp_2_Mo_2_(CO)_4_(η^2^-P_2_)^86^ were prepared according to literature procedures. NEt_3_, NHEt_2_, NH_2_^*i*^Pr, NH_2_^*t*^Bu, pyridine (Py), 4-(dimethylamino)­pyridine (DMAP), 4,4′-bipyridine (bipy) and 1,2-bis­(diphenylphosphino)­ethane (dppe) were obtained from Sigma-Aldrich. NHEt_2_, NH_2_^*i*^Pr, NH_2_^*t*^Bu and pyridine were used to prepare stock solutions in toluene, which were stored over the molecular sieves.

### Safety Statement

No uncommon hazards are noted.

### Synthetic Procedures

#### [IDipp•GeH_2_BH_2_•NEt_3_]­[OTf] (**2**)

IDipp·GeH_2_BH_2_OTf (1) (125 mg, 0.2 mmol, 1 equiv) was dissolved in 10 mL of Et_2_O, then NEt_3_ (25 mg, 0.25 mmol, 1.25 equiv) was added to the solution dropwise. The reaction mixture was stirred for two hours, then the mother liquor was decanted and the residue washed with 5 mL of Et_2_O. All volatiles were removed *in vacuo* and the product [IDipp·GeH_2_BH_2_•NEt_3_]­[OTf] (2) could be isolated as a white powder (125 mg, 86%). To obtain crystals suitable for X-ray analysis a concentrated DCM solution of the product was layered with 3-fold excess of *n*-hexane and stored at +9 °C. ^**1**^ **H NMR** (CD_2_Cl_2_, 500 MHz, 298 K): δ [ppm] = 0.95 (t, 9H, ^*3*^ *J*_*H,H*_ = 7.3 Hz, CH_2_C*H*_3_), 1.23 (d, 12H, ^*3*^ *J*_*H,H*_ = 6.8 Hz, CH­(C*H*_3_)_2_), 1.37 (d,12H, ^*3*^ *J*_*H,H*_ = 6.7 Hz, CH­(C*H*_3_)_2_), 1.54 (br, 2H, B*H*_2_), 2.40 (h, 4H, ^*3*^ *J*_*H,H*_ = 6.8 Hz, C*H*(CH_3_)_2_), 2.56 (t, 6H, ^*3*^ *J*_*H,H*_ = 7.3 Hz, C*H*_2_CH_3_), 3.57 (m, 2H, Ge*H*_2_), 7.43 (d, 4H, ^*3*^ *J*_*H,H*_ = 7.9 Hz, Ar*H*), 7.61 (s, 2H, N–C*H*), 7.64 (t, 2H, ^*3*^ *J*_*H,H*_ = 7.9 Hz, Ar*H*). ^**11**^ **B NMR** (CD_2_Cl_2_, 160.5 MHz, 298 K): δ [ppm] = −15.7 (t, br, BH_2_). ^**11**^ **B­{** ^**1**^ **H} NMR** (CD_2_Cl_2_, 160.5 MHz, 298 K): δ [ppm] = −15.7 (s, br, BH_2_). ^**19**^ **F NMR** (CD_2_Cl_2_, 470 MHz, 298 K): δ [ppm] = −79.09 (s, CF_3_). **ESI-MS** (pos. mod., o-DFB): *m*/*z* = 578.37 (100%, [IDipp·GeH_2_BH_2_·NEt_3_]^+^); **ESI-MS** (neg. mod., o-DFB): *m*/*z* = 148.95 (100%, [CF_3_SO_3_]^−^). **Elemental analysis** (%) calculated for C_37_H_55_N_3_GeBF_3_O_3_S: C: 56.22, H: 7.63, N: 5.79, S: 4.41; found: C: 56.23, H: 7.58, N: 5.63, S: 4.18.

#### [IDipp•BH_2_•NHEt_2_]­[OTf] (**3a**)

IDipp·GeH_2_BH_2_OTf (**1**) (63 mg, 0.1 mmol, 1 equiv) was dissolved in 10 mL of toluene, then the stock solution of NHEt_2_ (0.1 mmol, 1 equiv) was added to the reaction mixture dropwise. The reaction mixture was stirred for two hours, then all the volatiles were removed, and the residue was washed with 5 mL of *n*-hexane. The solids were dried in vacuo and the mixture of products [IDipp·BH_2_•NHEt_2_]­[OTf] (**3a**), [IDipp•GeH_3_]­[OTf] (**4**) and [IDippH]­[OTf] could be isolated as a white powder. To obtain crystals of **3a** suitable for the X-ray analysis the concentrated DCM solution of the product was layered with 3-fold excess of hexane and stored at +9 °C. Due to the severe imposition of the ^1^H signals from IDipp-carbene of **3a**, **4** and [IDippH]­[OTf] a full assignment of the signals becomes complicated. However, the presence of those compounds can be determined by the NHEt_2_ signals of **3a**, Ge*H*_3_ hydrides signal from **4** (δ = 4.00 ppm)^80^ and the imidazolium proton signal of [IDipp*H*]­[OTf] (δ = 9.10 ppm). ^**1**^ **H NMR** (CH_2_Cl_2_/C_6_D_6_, 500 MHz, 298 K): δ [ppm] = 0.77 (t, 6H, ^*3*^ *J*_*H,H*_ = 7.1 Hz, NH­(CH_2_C*H*_3_)_2_), 1.17 (d, 12H, ^*3*^ *J*_*H,H*_ = 6.8 Hz, CH­(C*H*_3_)_2_), 1.30 (d,12H, ^*3*^ *J*_*H,H*_ = 6.9 Hz, CH­(C*H*_3_)_2_), 1.82 (br, 2H, B*H*_2_), 2.35 (m, C*H*(CH_3_)_2_), 2.47 (m, NH­(C*H*_2_CH_3_)_2_), 3.24 (s, NH­(C*H*_2_CH_3_)_2_), 7.37 (d, 4H, ^*3*^ *J*_*H,H*_ = 7.8 Hz, Ar*H*), 7.60 (s, N–C*H*), 7.61 (t, Ar*H*). ^**11**^ **B NMR** (CH_2_Cl_2_/C_6_D_6_, 160.5 MHz, 298 K): δ [ppm] = −16.2 (t, br, BH_2_). ^**11**^ **B­{** ^**1**^ **H} NMR** (CH_2_Cl_2_/C_6_D_6_, 160.5 MHz, 298 K): δ [ppm] = −16.2 (s, br, BH_2_). ^**19**^ **F NMR** (CH_2_Cl_2_/C_6_D_6_, 470 MHz, 298 K): δ [ppm] = −79.09 (s, CF_3_). **ESI-MS** (pos. mod., o-DFB): *m*/*z* = 474.31 (100%, [IDipp·GeH_2_BH_2_·NHEt_2_]^+^).

#### [IDipp•BH_2_•NH_2_^*i*^Pr]­[OTf] (**3b**)

IDipp·GeH_2_BH_2_OTf (**1**) (63 mg, 0.1 mmol, 1 equiv) was dissolved in 10 mL of toluene, then a stock solution of NH_2_^*i*^Pr (0.1 mmol, 1 equiv) was added dropwise to the reaction mixture. The reaction mixture was stirred for two hours, then all the volatiles were removed and the residue was washed with 5 mL of hexane. Then the volatiles were removed in vacuo and the mixture of products [IDipp·BH_2_•NH_2_^*i*^Pr]­[OTf] (**3b**), [IDipp•GeH_3_]­[OTf] (**4**) and [IDippH]­[OTf] could be isolated as a white powder. To obtain crystals of **3b** suitable for the X-ray analysis, a concentrated DCM solution of the product was layered with 3-fold excess of *n*-hexane and stored at +9 °C. Due to the severe imposition of the ^1^H signals from IDipp carbene of **3b**, **4**, [IDippH]­[OTf] and ^*i*^Pr group of NH_2_^*i*^Pr a full assignment of the signals becomes complicated. However, the presence of those compounds can be determined by the N*H*_2_^*i*^Pr signal of **3b**, Ge*H*_3_ hydrides signal from **4** (δ = 3.93 ppm)^80^ and the imidazolium proton signal of [IDipp*H*]­[OTf] (δ = 8.95 ppm). ^**1**^ **H NMR** (CD_2_Cl_2_, 400 MHz, 298 K): δ [ppm] = 1.04 (d, 6H, ^*3*^ *J*_*H,H*_ = 6.8 Hz, NH_2_CH­(C*H*_3_)_2_), 1.08 (d, 12H, ^*3*^ *J*_*H,H*_ = 6.8 Hz, CH­(C*H*_3_)_2_), 1.22 (d,12H, ^*3*^ *J*_*H,H*_ = 6.9 Hz, CH­(C*H*_3_)_2_), 2.24 (s, 2H, N*H*_2_CH­(CH_3_)_2_), 2.18­(m, NH_2_C*H*(CH_3_)_2_), 2.31 (h, 4H, ^*3*^ *J*_*H,H*_ = 6.7 Hz, C*H*(CH_3_)_2_), 2.63 (br, 2H, B*H*_2_), 7.28 (d, 4H, ^*3*^ *J*_*H,H*_ = 8.0 Hz, Ar*H*), 7.36 (s, N–C*H*), 7.48 (t, 2H, ^*3*^ *J*_*H,H*_ = 7.7 Hz, Ar*H*). ^**11**^ **B NMR** (CD_2_Cl_2_, 128 MHz, 298 K): δ [ppm] = −8.2 (t, br, ^*1*^ *J*_*B,H*_= 100 Hz, BH_2_). ^**11**^ **B­{** ^**1**^ **H} NMR** (CD_2_Cl_2_, 128 MHz, 298 K): δ [ppm] = −8.2 (s, br, BH_2_). ^**19**^ **F NMR** (CD_2_Cl_2_, 376.5 MHz, 298 K): δ [ppm] = −76.24 (s, CF_3_). **ESI-MS** (pos. mod., o-DFB): *m*/*z* = 460.39 (100%, [IDipp·GeH_2_BH_2_·NH_2_^*i*^Pr]^+^).

#### [IDipp•BH_2_•NH_2_^*t*^Bu]­[OTf] (**3c**)

IDipp·GeH_2_BH_2_OTf (**1**) (63 mg, 0.1 mmol, 1 equiv) was dissolved in 10 mL of toluene, then a stock solution of NH_2_^*t*^Bu (0.1 mmol, 1 equiv) was added dropwise to the reaction mixture. The reaction mixture was stirred for two hours, then all the volatiles were removed and the residue was washed with 5 mL of *n*-hexane. The volatiles were removed in vacuo and the mixture of products [IDipp·BH_2_·NH_2_^*t*^Bu]­[OTf] (**3c**), [IDipp·GeH_3_]­[OTf] (**4**) and [IDippH]­[OTf] could be isolated as a white powder. To obtain crystals of **3c** suitable for the X-ray analysis, a concentrated DCM solution of the product was layered with 3-fold excess of *n*-hexane and stored at +9 °C. Due to the severe imposition of the ^1^H signals from IDipp carbene of **3b**, **4**, [IDippH]­[OTf] and ^*i*^Pr group of NH_2_^*i*^Pr a full assignment of the signals becomes complicated. However, the presence of those compounds can be determined by the N*H*_2_^*i*^Pr signal of **3b**, Ge*H*_3_ hydrides signal from **4** (δ = 4.58 ppm)^80^ and the imidazolium proton signal of [IDipp*H*]­[OTf] (δ = 9.14 ppm). ^**1**^ **H NMR** (CH_2_Cl_2_/C_6_D_6_, 500 MHz, 298 K): δ [ppm] = 0.85 (s, 9H, NH_2_C­(C*H*_3_)_3_, 1.17 (d, 12H, ^*3*^ *J*_*H,H*_ = 6.8 Hz, CH­(C*H*_3_)_2_), 1.29 (d,12H, ^*3*^ *J*_*H,H*_ = 6.8 Hz, CH­(C*H*_3_)_2_), 1.81 (br, 2H, B*H*_2_), 2.44 (h, 4H, ^*3*^ *J*_*H,H*_ = 6.9 Hz, C*H*(CH_3_)_2_), 3.45 (s, 2H, N*H*_2_C­(CH_3_)_3_, 7.33 (s, 2H, N–C*H*), 7.40 (d, 4H, ^*3*^ *J*_*H,H*_ = 7.8 Hz, Ar*H*), 7.60 (t, 2H, ^*3*^ *J*_*H,H*_ = 7.8 Hz, Ar*H*). ^**11**^ **B NMR** (CH_2_Cl_2_/C_6_D_6_, 160.5 MHz, 298 K): δ [ppm] = −21.4 (t, br, BH_2_). ^**11**^ **B­{** ^**1**^ **H} NMR** (CH_2_Cl_2_/C_6_D_6_, 160.5 MHz, 298 K): δ [ppm] = −21.4 (s, br, BH_2_). ^**19**^ **F NMR** (CH_2_Cl_2_/C_6_D_6_, 470 MHz, 298 K): δ [ppm] = −78.92 (s, CF_3_).

#### [IDipp•GeH_2_BH_2_•Py]­[OTf] (**5**)

IDipp·GeH_2_BH_2_OTf (**1**) (100 mg, 0.16 mmol, 1 equiv) was suspended in 10 mL of toluene, then the solution of pyridine (13 mg, 0.16 mmol, 1 equiv) was added dropwise. The reaction mixture was stirred for one hour, then all the volatiles were removed *in vacuo* and the product was extracted with DCM. The solution was filtered, the volatiles were removed again *in vacuo* and the product [IDipp·GeH_2_BH_2_·Py]­[OTf] (**5**) could be isolated as a white powder (80 mg, 71%). To obtain crystals suitable for the X-ray analysis, a concentrated DCM solution of the product was layered with 3-fold excess of hexane and stored at +9 °C. ^**1**^ **H NMR** (CD_2_Cl_2_, 400 MHz, 298 K): δ [ppm] = 1.12 (d, 12H, ^*3*^ *J*_*H,H*_ = 6.8 Hz, CH­(C*H*_3_)_2_), 1.20 (d, 12H, ^*3*^ *J*_*H,H*_ = 6.8 Hz, CH­(C*H*_3_)_2_), 2.31 (m, 4H, ^*3*^ *J*_*H,H*_ = 6.8 Hz, C*H*(CH_3_)_2_), 2.58 (br, 2H, B*H*_2_), 3.44 (t, ^*1*^ *J*_*Ge,H*_ = 4.2 Hz, 2H, Ge*H*_2_), 7.33 (d, 4H, ^*3*^ *J*_*H,H*_ = 7.9 Hz, Dipp-Ar*H*), 7.42 (t, 2H, ^*3*^ *J*_*H,H*_ = 7.2 Hz, Py-Ar*H*), 7.54 (s, 2H, N–C*H*), 7.56 (t, 2H, ^*3*^ *J*_*H,H*_ = 7.8 Hz, Dipp-Ar*H*), 7.83 (d, 2H, ^*3*^ *J*_*H,H*_ = 5.3 Hz, Py-Ar*H*), 7.94 (t, 1H, ^*3*^ *J*_*H,H*_ = 7.7 Hz, Py-Ar*H*). ^**11**^ **B NMR** (CD_2_Cl_2_, 128 MHz, 298 K): δ [ppm] = −14.0 (t, br, BH_2_). ^**11**^ **B­{** ^**1**^ **H} NMR** (CD_2_Cl_2_, 128 MHz, 298 K): δ [ppm] = −14.0 (s, br, BH_2_). ^**19**^ **F NMR** (CD_2_Cl_2_, 376.5 MHz, 298 K): δ [ppm] = −78.80 (s, CF_3_). **ESI-MS** (pos. mod., o-DFB): *m*/*z* = 556.29 (100%, [IDipp·GeH_2_BH_2_·Py]^+^); **ESI-MS** (neg. mod., o-DFB): *m*/*z* = 148.95 (100%, [CF_3_SO_3_]^−^). **Elemental analysis** (%) calculated for C_33_H_45_N_3_GeBF_3_O_3_S: C: 56.28, H: 6.44, N: 5.97, S: 4.55; found: C: 56.33, H: 6.64, N: 5.93, S: 4.60.

#### [IDipp•GeH_2_BH_2_•DMAP]­[OTf] (**6**)

IDipp·GeH_2_BH_2_OTf (**1**) (63 mg, 0.1 mmol, 1 equiv) and DMAP (12 mg, 0.1 mmol, 1 equiv) were combined in one flask, then suspended in toluene. The reaction mixture was stirred for one hour, all the volatiles were removed *in vacuo* and the product was extracted with DCM. The solution was filtered, then the volatiles were removed again *in vacuo* and the product [IDipp·GeH_2_BH_2_•DMAP]­[OTf] (**6**) could be isolated as a white powder (55 mg, 73%). ^**1**^ **H NMR** (CD_2_Cl_2_, 400 MHz, 298 K): δ [ppm] = 1.20 (d, 12H, ^*3*^ *J*_*H,H*_ = 7.0 Hz, CH­(C*H*_3_)_2_), 1.30 (d, 12H, ^*3*^ *J*_*H,H*_ = 6.8 Hz, CH­(C*H*_3_)_2_), 2.40 (h, 4H, ^*3*^ *J*_*H,H*_ = 6.9 Hz, C*H*(CH_3_)_2_), 2.02 (br, 2H, B*H*_2_), 3.04 (s, 6H, DMAP-CH_3_), 3.44 (t, ^*1*^ *J*_*Ge,H*_ = 4.4 Hz, 2H, Ge*H*_2_), 7.33 (d, 4H, ^*3*^ *J*_*H,H*_ = 7.9 Hz, Dipp-Ar*H*), 7.42 (t, 2H, ^*3*^ *J*_*H,H*_ = 7.2 Hz, DMAP-Ar*H*), 7.54 (s, 2H, N–C*H*), 7.56 (t, 2H, ^*3*^ *J*_*H,H*_ = 7.8 Hz, Dipp-Ar*H*), 7.83 (d, 2H, ^*3*^ *J*_*H,H*_ = 5.3 Hz, DMAP-Ar*H*), 7.94 (t, 1H, ^*3*^ *J*_*H,H*_ = 7.7 Hz, Py-Ar*H*). ^**11**^ **B NMR** (CD_2_Cl_2_, 128 MHz, 298 K): δ [ppm] = −15.4 (t, br, BH_2_). ^**11**^ **B­{** ^**1**^ **H} NMR** (CD_2_Cl_2_, 128 MHz, 298 K): δ [ppm] = −14.0 (s, br, BH_2_). ^**19**^ **F NMR** (CD_2_Cl_2_, 376.5 MHz, 298 K): δ [ppm] = −78.80 (s, CF_3_). **ESI-MS** (pos. mod., o-DFB): *m*/*z* = 599.33 (75%, [IDipp·GeH_2_BH_2_·DMAP]^+^); **ESI-MS** (neg. mod., o-DFB): *m*/*z* = 148.95 (100%, [CF_3_SO_3_]^−^). **Elemental analysis** (%) calculated for C_35_H_50_N_4_GeBF_3_O_3_S: C: 56.25, H: 6.74, N: 7.50, S 4.29; found: C: 55.98, H: 6.30, N: 7.59, S: 4.25.

#### [IDipp•GeH_2_BH_2_•bipy•BH_2_GeH_2_•IDipp]­[OTf]_2_ (**7**)

IDipp·GeH_2_BH_2_OTf (**1**) (125 mg, 0.2 mmol, 2 equiv) and bipy (16 mg, 0.1 mmol, 1 equiv) were combined in one flask, then suspended in toluene. The reaction mixture was stirred for one hour, then all the volatiles were removed *in vacuo*, the residue was washed with *n*-hexane and then dried. The product [IDipp·GeH_2_BH_2_•bipy•BH_2_GeH_2_·IDipp]­[OTf]_2_ (**7**) could be isolated as a white powder (116 mg, 83%). To obtain crystals suitable for the SC-XRD a concentrated DCM solution of the product was layered with 3-fold excess of *n*-hexane and stored at +9 °C. ^**1**^ **H NMR** (CD_2_Cl_2_, 500 MHz, 298 K): δ [ppm] = 1.11 (d, 12H, ^*3*^ *J*_*H,H*_ = 6.8 Hz, CH­(C*H*_3_)_2_), 1.22 (d, 12H, ^*3*^ *J*_*H,H*_ = 6.8 Hz, CH­(C*H*_3_)_2_), 2.30 (h, 4H, ^*3*^ *J*_*H,H*_ = 6.9 Hz, C*H*(CH_3_)_2_), 2.55 (br, 2H, B*H*_2_), 3.48 (br t, ^*1*^ *J*_*Ge,H*_ = 3.5 Hz, 2H, Ge*H*_2_), 7.35 (d, 4H, ^*3*^ *J*_*H,H*_ = 7.8 Hz, Dipp-Ar*H*), 7.47 (s, 2H, N–C*H*), 7.54 (t, 2H, ^*3*^ *J*_*H,H*_ = 7.2 Hz, Py-Ar*H*), 7.59 (t, 2H, ^*3*^ *J*_*H,H*_ = 7.8 Hz, Dipp-Ar*H*), 7.97 (d, 2H, ^*3*^ *J*_*H,H*_ = 6.8 Hz, bipy-Ar*H*), 8.00 (d, 2H, ^*3*^ *J*_*H,H*_ = 6.8 Hz, bipy-Ar*H*). ^**11**^ **B NMR** (CD_2_Cl_2_, 160.5 MHz, 298 K): δ [ppm] = −12.6 (br, BH_2_). ^**11**^ **B­{** ^**1**^ **H} NMR** (CD_2_Cl_2_, 160.5 MHz, 298 K): δ [ppm] = −12.6 (br, BH_2_). ^**19**^ **F NMR** (CD_2_Cl_2_, 470 MHz, 298 K): δ [ppm] = −78.77 (s, CF_3_). **ESI-MS** (pos. mod., o-DFB): *m*/*z* = 553.79 (2%, [IDipp·GeH_2_BH_2_•bipy•BH_2_GeH_2_·IDipp]^+^). An intensive signal for IDippH+ (*m*/*z* = 389.29) arises due to decomposition in conditions of mass spectrometry; **ESI-MS** (neg. mod., o-DFB): *m*/*z* = 148.95 (100%, [CF_3_SO_3_]^−^). **Elemental analysis** (%) calculated for C_66_H_88_N_6_Ge_2_B_2_F_6_O_6_S_2_: C: 56.36, H: 6.31, N: 5.98, S: 4.56; found: C: 56.72, H: 6.87, N: 6.19, S: 4.95.

#### [IDipp•GeH_2_BH_2_•dppe•BH_2_GeH_2_•IDipp]­[OTf]_2_ (**8**)

IDipp·GeH_2_BH_2_OTf (**1**) (125 mg, 0.2 mmol, 2 equiv) and dppe (40 mg, 0.1 mmol, 1 equiv) were combined in one flask, then suspended in toluene. The reaction mixture was stirred for one hour, all the volatiles were removed *in vacuo* and the product was extracted with DCM. The solution was decanted from precipitates, concentrated and stored at −30 °C to obtain the product [IDipp·GeH_2_BH_2_•dppe•BH_2_GeH_2_·IDipp]­[OTf]_2_ (**8**) as colorless crystals (130 mg, 79%). ^**1**^ **H NMR** (CD_2_Cl_2_, 500 MHz, 298 K): δ [ppm] = 0.69 (br, 2H, B*H*_2_), 1.22 (d, 24H, ^*3*^ *J*_*H,H*_ = 6.9 Hz, CH­(C*H*_3_)_2_), 1.84 (d, 2H, ^*2*^ *J*_*H,p*_ = 3.0 Hz, P­(CH_2_)_2_, 2.34 (h, 4H, ^*3*^ *J*_*H,H*_ = 6.8 Hz, C*H*(CH_3_)_2_), 3.25 (t, ^*1*^ *J*_*Ge,H*_ = 4.6 Hz, 2H, Ge*H*_2_), 7.03 (dd, 4H, ^*3*^ *J*_*H,H*_ = 7.0 Hz, ^*3*^ *J*_*H,P*_ = 11.5 Hz, dppe-*o*-Ar*H*) 7.38 (d, 4H, ^*3*^ *J*_*H,H*_ = 7.8 Hz, Dipp-*m*-Ar*H*), 7.41 (t, 4H, ^*3*^ *J*_*H,H*_ = 7.8 Hz, dppe-*m*-Ar*H*), 7.62 (t, 4H, ^*3*^ *J*_*H,H*_ = 7.8 Hz, Dipp-*p*-Ar*H* + dppe-*p*-Ar*H*), 7.70 (s, 2H, N–C*H*). ^**11**^ **B NMR** (CD_2_Cl_2_, 160.5 MHz, 298 K): δ [ppm] = −41.8 (br, BH_2_). ^**11**^ **B­{** ^**1**^ **H} NMR** (CD_2_Cl_2_, 160.5 MHz, 298 K): δ [ppm] = −41.8 (br, BH_2_). ^**31**^ **P NMR** (CD_2_Cl_2_, 202.5 MHz, 298 K): δ [ppm] = 18.6 (br, dppe-P). ^**31**^ **P­{** ^**1**^ **H} NMR** (CD_2_Cl_2_, 202.5 MHz, 298 K): δ [ppm] = 18.6 (br, dppe-P). ^**19**^ **F NMR** (CD_2_Cl_2_, 470 MHz, 298 K): δ [ppm] = −78.85 (s, CF_3_). **ESI-MS** (pos. mod., o-DFB): *m*/*z* = 675.32 (9%, [IDipp·GeH_2_BH_2_•dppe•BH_2_GeH_2_·IDipp]^+^). An intensive signal for IDippH+ (*m*/*z* = 389.29) arises due to decomposition in conditions of mass spectrometry.; **ESI-MS** (neg. mod., o-DFB): *m*/*z* = 148.95 (100%, [CF_3_SO_3_]^−^). **Elemental analysis** (%) calculated for C_82_H_104_N_4_Ge_2_B_2_P_2_F_6_O_6_S_2_: C: 59.74, H: 6.36, N: 3.40, S: 3.89; found: C: 58.94, H: 5.89, N: 3.22, S: 3.96.

#### [Cp_2_Mo_2_(CO)_4_(η^2^-P_2_)•BH_2_GeH_2_•IDipp]­[OTf] (**9**)

IDipp·GeH_2_BH_2_OTf (**1**) (63 mg, 0.1 mmol, 1 equiv) and [Cp_2_Mo_2_(CO)_4_(η^2^-P_2_)] (50 mg, 0.1 mmol, 1 equiv) were combined in one flask and suspended in toluene. The reaction mixture was stirred for one and a half hours, all the volatiles were removed *in vacuo* and the product was extracted with DCM. The solution was concentrated, then layered with 3-fold excess of *n*-hexane and stored at +9 °C to obtain the product [Cp_2_Mo_2_(CO)_4_(η^2^-P_2_)·BH_2_GeH_2_·IDipp]­[OTf] (**9**) as red crystals (46 mg, 41%). ^**1**^ **H NMR** (CD_2_Cl_2_, 500 MHz, 298 K): δ [ppm] = 1.24 (d, 12H, ^*3*^ *J*_*H,H*_ = 6.8 Hz, CH­(C*H*_3_)_2_), 1.35 (d, 12H, ^*3*^ *J*_*H,H*_ = 6.9 Hz, CH­(C*H*_3_)_2_), 1.71 (br, 2H, B*H*_2_), 2.43 (h, 4H, ^*3*^ *J*_*H,H*_ = 6.9 Hz, C*H*(CH_3_)_2_), 3.56 (m, ^*1*^ *J*_*Ge,H*_ = 4.4 Hz, 2H, Ge*H*_2_), 5.28 (s, 10H, Cp-H), 7.43 (d, 4H, ^*3*^ *J*_*H,H*_ = 7.8 Hz, Dipp-Ar*H*), 7.60 (s, 2H, N–C*H*), 7.65 (t, 2H, ^*3*^ *J*_*H,H*_ = 7.8 Hz, Dipp-Ar*H*). ^**11**^ **B NMR** (CD_2_Cl_2_, 160.5 MHz, 298 K): δ [ppm] = −34.8 (br, BH_2_). ^**11**^ **B­{** ^**1**^ **H} NMR** (CD_2_Cl_2_, 160.5 MHz, 298 K): δ [ppm] = −34.8 (br, BH_2_). ^**31**^ **P NMR** (CD_2_Cl_2_, 202.5 MHz, 298 K): δ [ppm] = −18.5 (d, ^*1*^ *J*_*P,P*_ = 505 Hz, Mo_2_P_2_), −158.3 (d, ^*1*^ *J*_*P,P*_ = 505 Hz, Mo_2_P_2_). ^**31**^ **P­{** ^**1**^ **H} NMR** (CD_2_Cl_2_, 202.5 MHz, 298 K): δ [ppm] = −18.5 (d, ^*1*^ *J*_*P,P*_ = 505 Hz, Mo_2_P_2_), −158.3 (d, ^*1*^ *J*_*P,P*_ = 505 Hz, Mo_2_P_2_). ^**19**^ **F NMR** (CD_2_Cl_2_, 470 MHz, 298 K): δ [ppm] = −78.89 (s, CF_3_). **ESI-MS** (pos. mod., o-DFB): *m*/*z* = 973.07 (0.3%, [Cp_2_Mo_2_(CO)_4_(η^2^-P_2_)•BH_2_GeH_2_·IDipp]^+^). An intensive signal for IDippH+ (*m*/*z* = 389.29) arises due to decomposition in conditions of mass spectrometry.; **ESI-MS** (neg. mod., o-DFB): *m*/*z* = 148.95 (100%, [CF_3_SO_3_]^−^). **Elemental analysis** (%) calculated for C_42_H_50_N_2_GeBF_3_O_7_SMo_2_P_2_: C: 44.99, H: 4.50, N: 2.50, S: 2.86; found: C: 45.75, H: 4.72, N: 2.97, S: 3.22.

Details about anion exchange experiments and the reaction with TMEDA are given in Section 1 of the Supporting Information. The NMR data can be found in Section 2 of the Supporting Information.

### X-ray Crystallographic Details

Single-crystal X-ray diffraction experiments were performed on a XtaLAB Synergy R DW system (Rigaku) equipped with a HyPix-Arc 150 detector. Data were collected using Cu–Kα radiation (λ = 1.54178 Å). Data reduction, scaling and absorption corrections were performed using CrysAlisPro^90^ (Rigaku). Using Olex2 ^91^ all structures were solved with ShelXT^92^ and a least-squares refinement on *F* ^*2*^ was carried out with ShelXL.^93^ All non-hydrogen atoms were refined anisotropically. All the hydrogen atoms at the carbon atoms have been located in idealized positions and refined isotropically according to the riding model. Figures were created with Olex2. Crystallographic data and details of the experiments are given in Tables S1, S2 and S3 in the Supporting Information. CIF files with comprehensive information on the details of the diffraction experiments and full tables of bond lengths and angles are deposited in the Cambridge Crystallographic Data Centre under the deposition codes CCDC 2525632–2525639. These data can be obtained free of charge at www.ccdc.cam.ac.uk/conts/retrieving.html (or from the Cambridge Crystallographic Data Centre, 12 Union Road, Cambridge CB2 1EZ, UK; Fax: + 44–1223–336–033; e-mail: deposit@ccdc.cam.ac.uk). Further crystallographic data can be found in Sections 3 and 4 of the Supporting Information.

### Computational Details

The geometries of the compounds have been fully optimized with gradient-corrected density functional theory (DFT) in form of Becke’s three-parameter hybrid method B3LYP^94,95^ with the def2-TZVP^96^ all electron basis set. The Gaussian 09 ^97^ program package was used throughout. All structures correspond to minima on their respective potential energy surfaces as verified by computation of second derivatives. Additional information is provided in Section 5 of the Supporting Information.

## Supplementary Material



## Notes

Crystallographic data and computational details (PDF)

## †

Imperial College London (White City Campus), London W12 0BZ, United Kingdom
