# Supplementary material for: Cationic Group 13/14/15 Element Chain Compounds with Pnictogen-Donor Ligands
Source: Inorg Chem. 2026 May 4;65(19):10622–31. doi: 10.1021/acs.inorgchem.6c00556 (PMC13188059; doi:10.1021/acs.inorgchem.6c00556)
Supplement: Supplementary file 1 [file ic6c00556_si_001.pdf]

Supplementary information

Cationic Group 13/14/15 Element Chain Compounds with Pnictogen-Donor Ligands

Tatiana N. Parfeniuk, Matthias T. Ackermann, Christoph Riesinger, Manfred Scheer*

Institute of Inorganic Chemistry, University of Regensburg

93053 Regensburg (Germany)

Homepage: http://www.uni-regensburg.de/chemie-pharmazie/anorganische-chemie-scheer

E-mail: manfred.scheer@ur.de

Table of Contents

1. Experimental Details 3

a. Anion exchange experiments 3

i. Synthesis of [IDipp·GeH_2BH_2][TEF] 3

ii. Reaction of [IDipp·GeH_2BH_2][TEF] with $NHEt_2$ 3

iii. Synthesis of [IDipp·GeH_2BH_2][BAr^F] 4

iv. Reaction of [IDipp·GeH_2BH_2][BAr^F] with $NHEt_2$ 4

v. Reaction of [IDipp·GeH_2BH_2][OTf] with $KBAr^F$ in o-DFB 5

vi. Reaction of [IDipp·GeH_2BH_2][OTf] with TlTEF in o-DFB 5

b. Reaction of IDipp·GeH_2BH_2OTf (1) with TMEDA 5

2. NMR data 6

a. [IDipp · GeH_2BH_2 · NEt_3][OTf] (2) 6

b. [IDipp · BH_2 · $NHEt_2$][OTf] (3a) 8

c. [IDipp · BH_2 · $NH_2{}^iPr$][OTf] (3b) 10

d. [IDipp · BH_2 · $NH_2{}^tBu$][OTf] (3c) 12

e. [IDipp · GeH_2BH_2 · Py][OTf] (5) 14

f. [IDipp · GeH_2BH_2 · DMAP][OTf] (6) 16

g. [IDipp · GeH_2BH_2 · bipy · BH_2GeH_2 · IDipp][OTf]$_2$ (7) 18

h. [IDipp · GeH_2BH_2 · dppe · BH_2GeH_2 · IDipp][OTf]$_2$ (8) 20

i. [$Cp_2Mo_2(CO)_4(\eta^2$-$P_2)$ · BH_2GeH_2 · IDipp][OTf] (9) 23

j. Anion exchange experiments 26

i. [IDipp·GeH_2BH_2][TEF] 26

ii. Reaction of [IDipp·GeH_2BH_2][TEF] with $NHEt_2$ 28
iii. [IDipp·GeH_2BH_2][BAr^F] 30
iv. Reaction of [IDipp·GeH_2BH_2][BAr^F] with $NHEt_2$ 32
v. Reaction of [IDipp·GeH_2BH_2][OTf] with $KBAr^F$ in o-DFB 34
vi. Reaction of [IDipp·GeH_2BH_2][OTf] with TlTEF in o-DFB 36
k. IDipp·GeH_2BH_2OTf (1) with TMEDA 38
3. Crystallographic data 40
4. Crystal Structures 44
a. [IDipp · GeH_2BH_2 · NEt_3][OTf] (2) 44
b. [IDipp · BH_2 · $NHEt_2$][OTf] (3a) 44
c. [IDipp · BH_2 · $NH_2{}^i$Pr][OTf] (3b) 45
d. [IDipp · BH_2 · $NH_2{}^t$Bu][OTf] (3c) 46
e. [IDipp · GeH_2BH_2 · Py][OTf] (5) 46
f. [IDipp · GeH_2BH_2 · bipy · BH_2GeH_2 · IDipp][OTf]$_2$ (7) 47
g. [IDipp · GeH_2BH_2 · dppe · BH_2GeH_2 · IDipp][OTf]$_2$ (8) 48
h. [$Cp_2Mo_2(CO)_4(\eta^2\text{-}P_2)$ · BH_2GeH_2 · IDipp][OTf] (9) 49
5. Computational Details 50

1. Experimental Details

a. Anion exchange experiments

i. Synthesis of [IDipp·GeH_2BH_2][TEF]

IDipp·GeH_2BH_2OTf (**1**) (125 mg, 0.2 mmol, 1 eq) and LiTEF (TEF = $[Al(OC(CF_3)_3)_4]^-$) (195 mg, 0.2 mmol, 1 eq) were combined in one flask as solids, then dissolved in Et_2O, stirred for 1 hour, then all the volatiles were removed, washed with pentane and dried.

The NMR spectra of the reaction mixture of **1** with LiTEF were recorded to control the process. Due to the overlap of the [IDipp·GeH_2BH_2][TEF] ^{1}H NMR signals with the solvent signals the complete assignment of the signals was complicated, so the presence of typical IDipp-carbene proton signals (and the absence of the [IDipp*H*][OTf] signal) together with present ^{11}B signal of BH_2-group and ^{19}F signal of TEF-anion were accounted as the evidence of successful anion exchange.

<u>[IDipp·GeH_2BH_2][TEF]</u>

^{1}H NMR (Et_2O/C_6D_6, 400 MHz, 298 K): δ [ppm] = 1.90 (d, 12H, $^3J_{H,H}$ = 6.9 Hz, CH(CH_3)$_2$), 2.03 (d,12H, $^3J_{H,H}$ = 7.0 Hz, CH(CH_3)$_2$), 3.09 (h, 4H, $^3J_{H,H}$ = 6.9 Hz, C*H*(CH_3)$_2$), 8.13 (d, 4H, $^3J_{H,H}$ = 7.8 Hz, Ar*H*), 8.33 (t, 2H, $^3J_{H,H}$ = 7.8 Hz, Ar*H*), 8.64 (s, 2H, N-C*H*). **^{11}B NMR** (Et_2O/C_6D_6, 128 MHz, 298 K): δ [ppm] = -2.23 (br, BH_2). **^{11}B{^{1}H} NMR** (Et_2O/C_6D_6, 128 MHz, 298 K): δ [ppm] = -2.23 (s, br, BH_2). **^{19}F NMR** (Et_2O/C_6D_6, 376.5 MHz, 298 K): δ [ppm] = -75.43 (s, CF_3).

ii. Reaction of [IDipp·GeH_2BH_2][TEF] with $NHEt_2$

The [IDipp·GeH_2BH_2][TEF] from the previous step (i) was redissolved in CH_2Cl_2, then $NHEt_2$ (0.2 mmol, 1 eq) was added dropwise. The rection mixture was stirred for 1 hour, then all the volatiles were removed.

In the ^{1}H NMR of the residue several IDipp-carbene species were observed, causing a severe overlap of their signals and complicating the assignment. However the signals of hydrides at Ge and B atoms could be observed as well as the signals of OEt_2 in the [IDipp·GeH_2BH_2]$^+$ to OEt_2 ratio 1:1, indicating the formation of [IDipp·GeH_2BH_2·OEt_2][TEF] complex in the absence of $NHEt_2$ signals. Moreover [IDipp·GeH_3][OTf] hydrides signal is present in ^{1}H NMR spectrum. In the ^{11}B also several signals are present, including the signal of IDipp·BH_3.

<u>[IDipp·GeH_2BH_2·OEt_2][TEF]</u>

^{1}**H NMR** (CD_2Cl_2, 400 MHz, 298 K): δ [ppm] = 1.12-1.38(two d, CH(CH_3)$_2$), 1.56 (t, 6H, $^3J_{H,H}$ = 7.2 Hz, O(CH_2CH_3)$_2$), 2.38 (h, 4H, $^3J_{H,H}$ = 6.8 Hz, CH(CH_3)$_2$), 2.64 (br, BH_2), 3.14 (m, 2H, GeH_2), 3.74 (q, 4H, $^3J_{H,H}$ = 7.2 Hz, O(C$H_2$$CH_3$)$_2$), 7.45 (d, 4H, $^3J_{H,H}$ = 7.8 Hz, ArH), 7.66 (t, 2H, $^3J_{H,H}$ = 7.9 Hz, ArH), 7.74 (s, 2H, N-CH). ^{11}**B NMR** (CD_2Cl_2, 128 MHz, 298 K): δ [ppm] = -2.6 (br, BH_2). ^{11}**B{**^{1}**H} NMR** (CD_2Cl_2, 128 MHz, 298 K): δ [ppm] = -2.6 (s, br, BH_2). ^{19}**F NMR** (CD_2Cl_2, 376.5 MHz, 298 K): δ [ppm] = -75.62 (s, CF_3). **ESI-MS** (pos. mod., o-DFB): m/z = 551.32 (17%, [IDipp·$GeH_2BH_2OEt_2$]$^+$); **ESI-MS** (neg. mod., o-DFB): m/z = 966.92 (100%, [TEF]$^-$).

iii. Synthesis of [IDipp·GeH_2BH_2][BAr^F]

IDipp·GeH_2BH_2OTf (**1**) (125 mg, 0.2 mmol, 1 eq) and KBAr^F (BAr^F = [B(C_6F_5)$_4$]$^-$) (144 mg, 0.2 mmol, 1 eq) were combined in one flask as solids, then dissolved in Et_2O, stirred for 1 hour. Then the solution was decanted from precipitates, solvent was removed *in vacuo*, the residue washed with pentane and dried.

The NMR spectra of the reaction mixture of **1** with KBAr^F were recorded to control the process. Due to the overlap of the [IDipp·GeH_2BH_2][BAr^F] ^{1}H NMR signals with the solvent signals the complete assignment of the signals was complicated, so the presence of typical IDipp-carbene proton together with present ^{11}B signal of BH_2-group and ^{19}F signals of BAr^F-anion were accounted as the evidence of successful anion exchange. However, the formation of [IDippH][A] and the presence of triflate anion in solution are observed.

[IDipp·GeH_2BH_2][BAr^F]

^{1}**H NMR** (Et_2O/C_6D_6, 400 MHz, 298 K): δ [ppm] = 1.80-2.00(two d, CH(CH_3)$_2$), 2.96 (h, 4H, $^3J_{H,H}$ = 6.9 Hz, CH(CH_3)$_2$), 8.04 (d, 4H, $^3J_{H,H}$ = 7.8 Hz, ArH), 8.22 (t, 2H, $^3J_{H,H}$ = 7.8 Hz, ArH), 8.82 (s, 2H, N-CH). ^{11}**B NMR** (Et_2O/C_6D_6, 128 MHz, 298 K): δ [ppm] = -1.84 (br, BH_2), -16.19 (s, br, BAr^F). ^{11}**B{**^{1}**H} NMR** (Et_2O/C_6D_6, 128 MHz, 298 K): δ [ppm] = -1.84 (br, BH_2), -16.19 (s, br, BAr^F). ^{19}**F NMR** (Et_2O/C_6D_6, 376.5 MHz, 298 K): δ [ppm] = -132.08 (s, BAr^F), -164.69 (t, BAr^F), -168.11 (m, BAr^F).

iv. Reaction of [IDipp·GeH_2BH_2][BAr^F] with $NHEt_2$

The [IDipp·GeH_2BH_2][BAr^F] from the previous step was redissolved in CH_2Cl_2, then $NHEt_2$ (0.2 mmol, 1 eq) was added dropwise. The rection mixture was stirred for 1 hour, Then the solution was decanted from precipitates and concentrated.

In the ^{1}H NMR of the residue mostly two IDipp-carbene species were observed, that could be attributed to the [IDipp · GeH_3][BAr^F] and [IDippH][BAr^F]. In the ^{11}B and ^{19}F NMR spectra only the BAr^{F-} signals are observed.

The ESI-MS experiment showed the presence of [IDipp·$GeH_2BH_2OEt_2$]$^+$ (m/z = 551.32 (0.8%,)), however the most intensive signal comes from [IDippH]$^+$.

v. Reaction of [IDipp·GeH_2BH_2][OTf] with KBAr^F in o-DFB

IDipp·GeH_2BH_2OTf (**1**) (63 mg, 0.1 mmol, 1 eq) and KBAr^F (BAr^F = [B(C_6F_5)$_4$]$^-$) (72 mg, 0.1 mmol, 1 eq) were combined in one flask as solids, then dissolved in o-DFB, stirred for 1 hour. Then the solution was decanted from precipitates, concentrated, layered with three-fold excess of hexane and stored at +9 °C to obtain the crystals. The crystalline product was isolated, washed with hexane and dried *in vacuo*. The NMR spectra showed only presence of [IDipp · GeH_3][BAr^F], indicating the decomposition.

vi. Reaction of [IDipp·GeH_2BH_2][OTf] with TlTEF in o-DFB

IDipp·GeH_2BH_2OTf (**1**) (63 mg, 0.1 mmol, 1 eq) and TlTEF (TEF = [Al(OC(CF_3)$_3$)$_4$]$^-$) (60 mg, 0.1 mmol, 1 eq) were combined in one flask as solids, then dissolved in o-DFB, stirred for 1 hour. Then the solution was decanted from precipitates, concentrated, layered with three-fold excess of hexane and stored at +9 °C to obtain the crystals. The crystalline product was isolated, washed with hexane and dried *in vacuo*. The NMR spectra showed only presence of [IDipp · GeH_3][TEF], indicating the decomposition.

b. Reaction of IDipp·GeH_2BH_2OTf (1) with TMEDA

IDipp·GeH_2BH_2OTf (**1**) (125 mg, 0.2 mmol, 1 eq) was dissolved in 10 mL of Et_2O, then TMEDA (35 mg, 0.3 mmol, 1.5 eq) was added to the solution dropwise. The reaction mixture was stirred overnight, then all volatiles were removed *in vacuo* and the product extracted with DCM. The NMR spectra showed the formation of [IDippH][OTf] and [BH_2·TMEDA][OTf].

2. NMR data

a. [IDipp·GeH_2BH_2·NEt_3][OTf] (2)

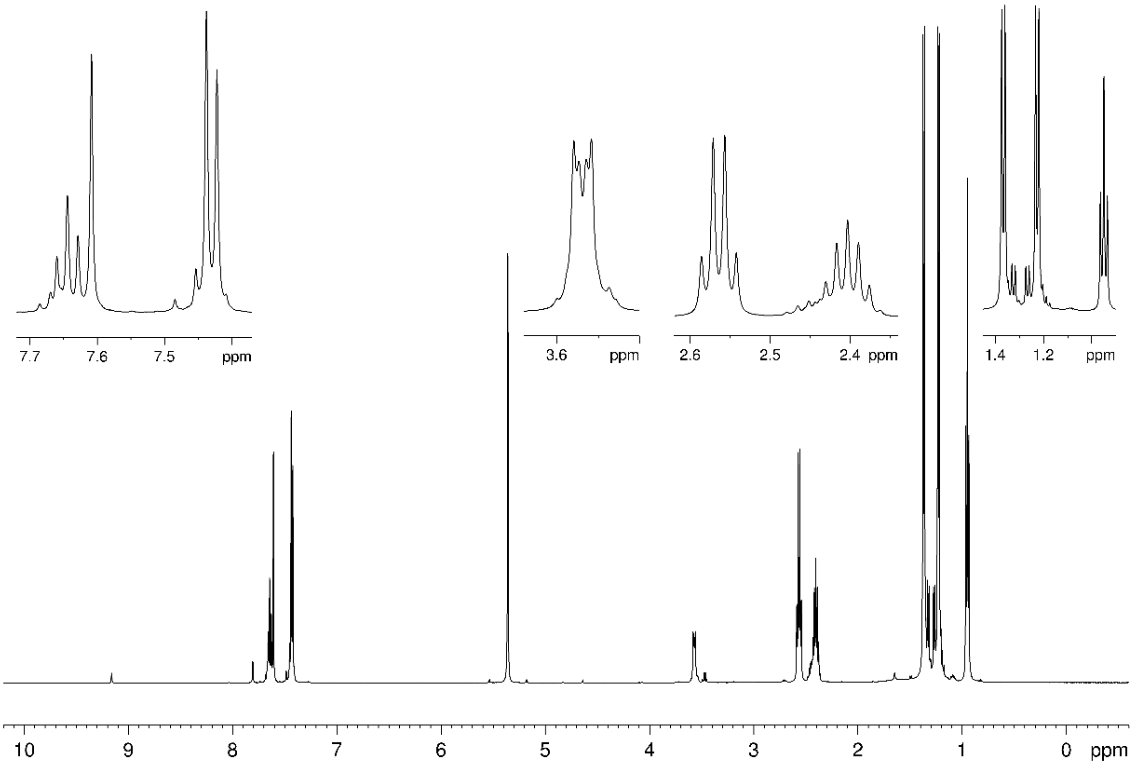

Figure S1. ^{1}H NMR spectrum of **2** in CD_2Cl_2 at 298 K.

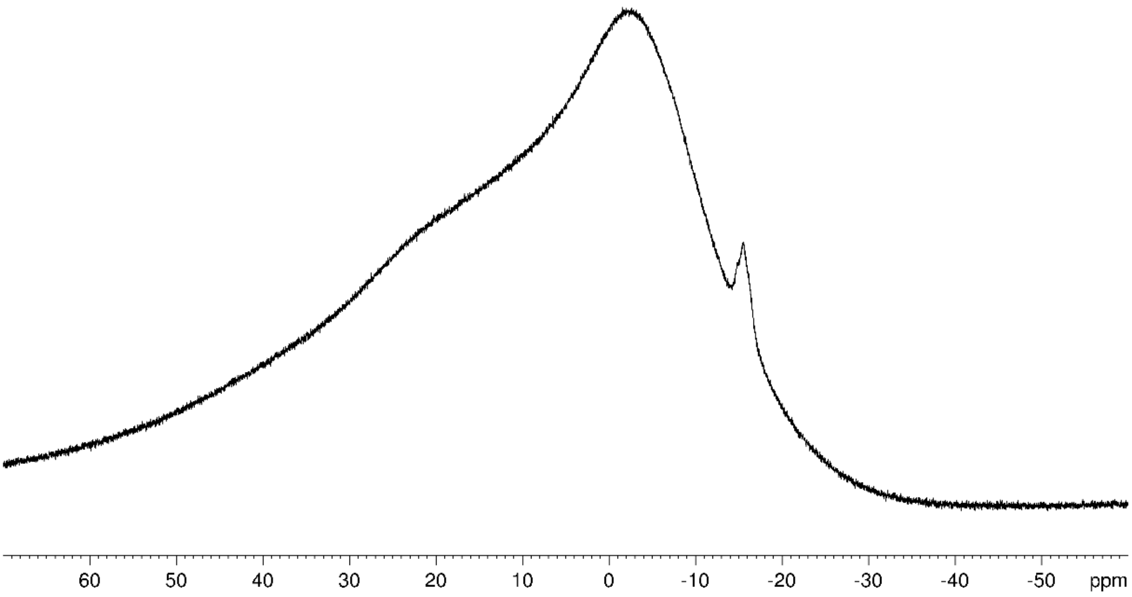

Figure S2. ^{11}B NMR spectrum of **2** in CD_2Cl_2 at 298 K.

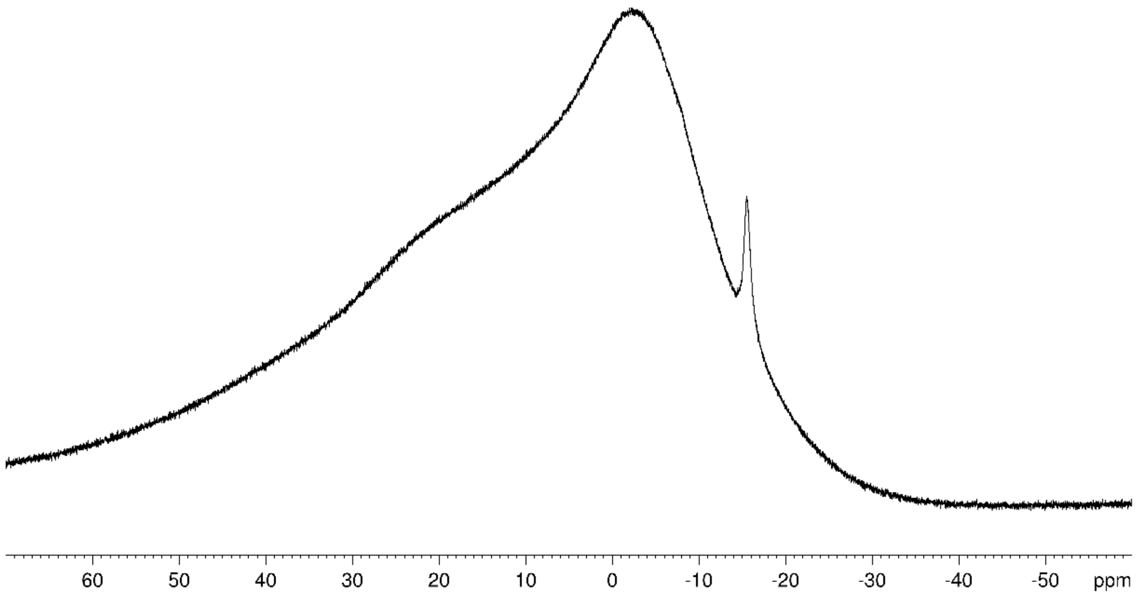

Figure S3. $^{11}B\{^{1}H\}$ NMR spectrum of **2** in CD_2Cl_2 at 298 K.

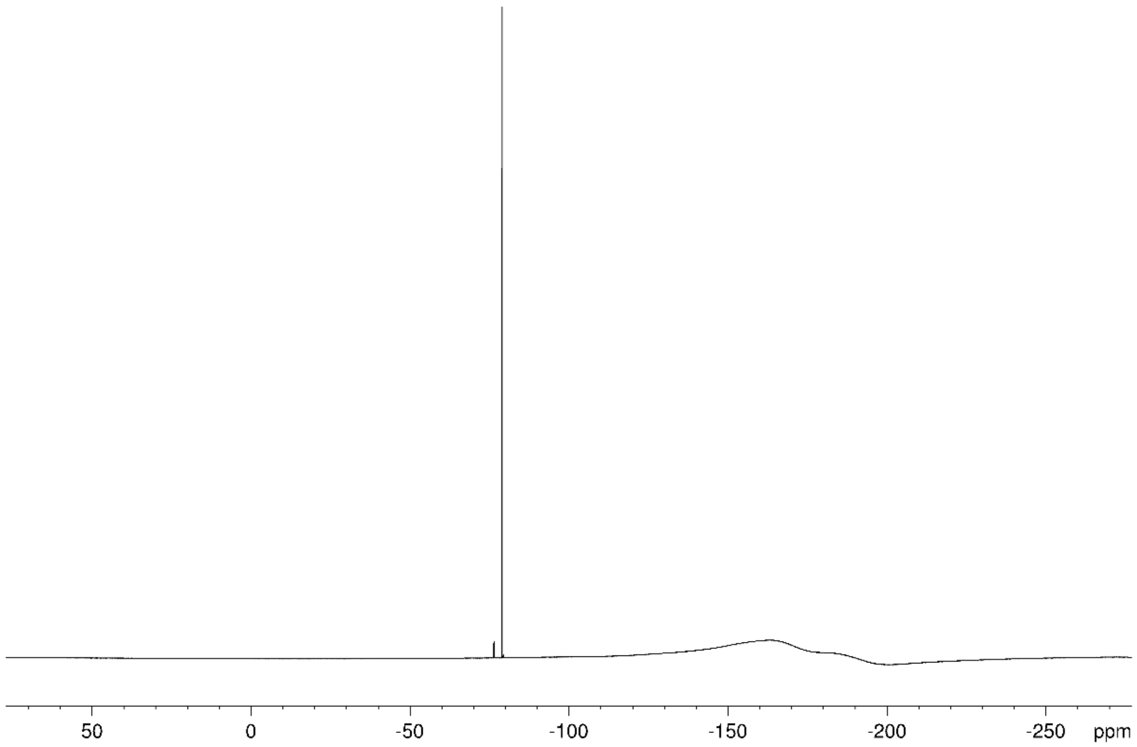

Figure S4. ^{19}F NMR spectrum of **2** in CD_2Cl_2 at 298 K.

b. [IDipp·BH_2·$NHEt_2$][OTf] (3a)

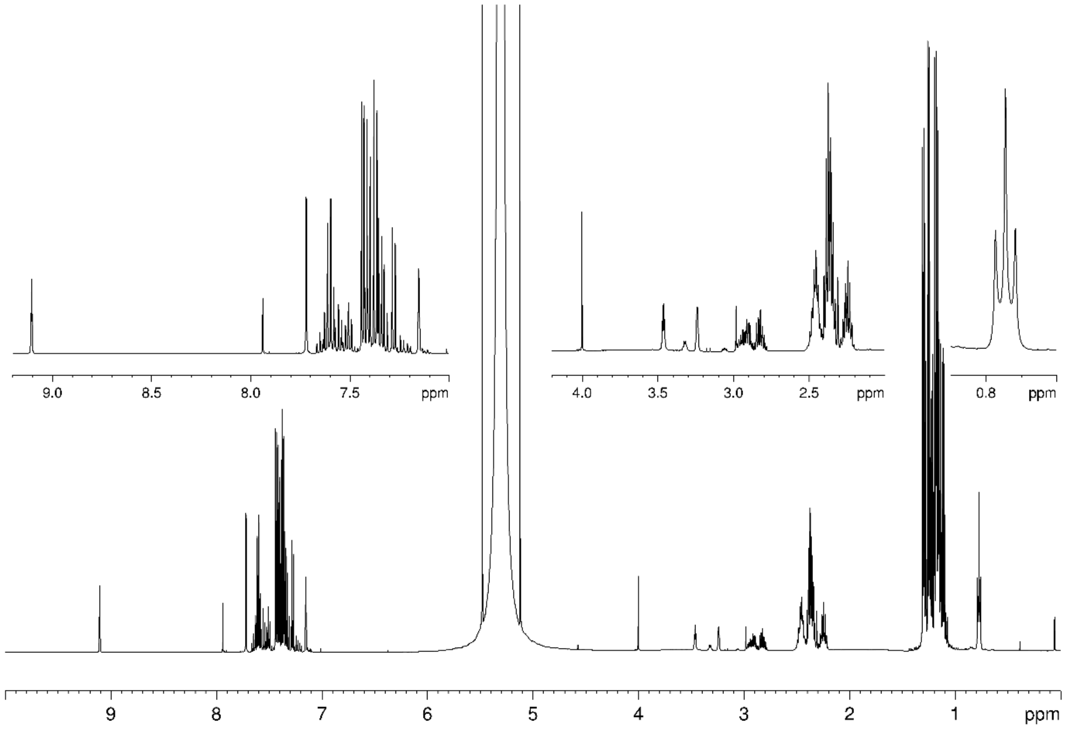

Figure S5. ^{1}H NMR spectrum of **3a** in CH_2Cl_2/C_6D_6 at 298 K.

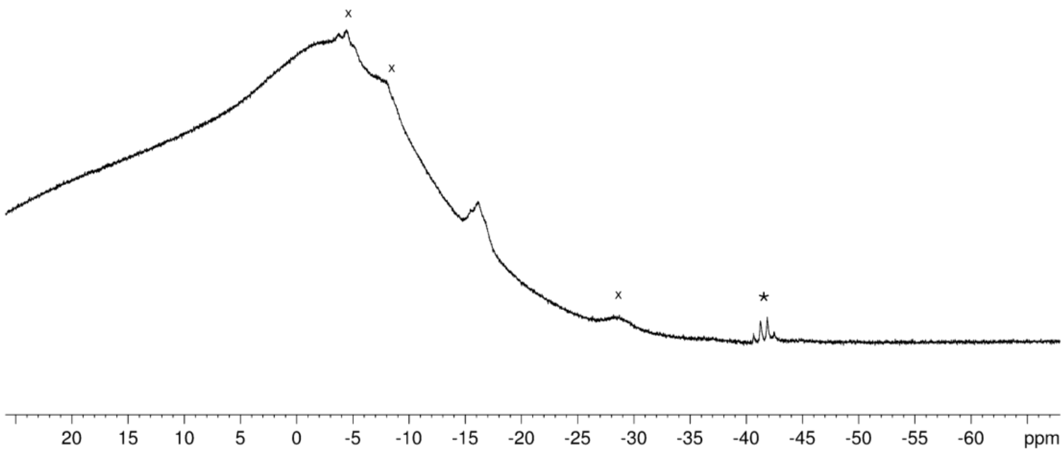

Figure S6. ^{11}B NMR spectrum of **3a** in CH_2Cl_2/C_6D_6 at 298 K. * - IDippBH$_3$, x – unidentified impurities.

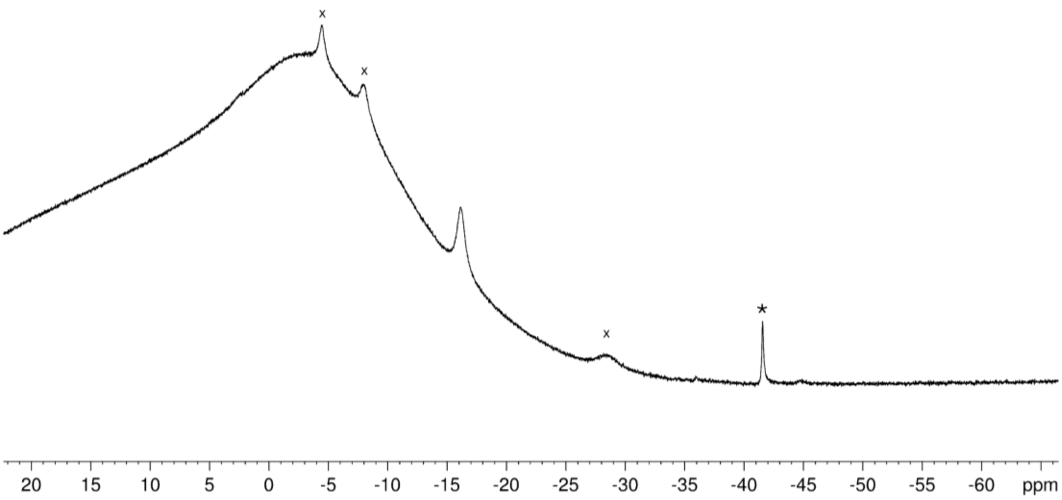

Figure S7. $^{11}B\{^{1}H\}$ NMR spectrum of **3a** in CH_2Cl_2/C_6D_6 at 298 K. * - $IDippBH_3$, x – unidentified impurities.

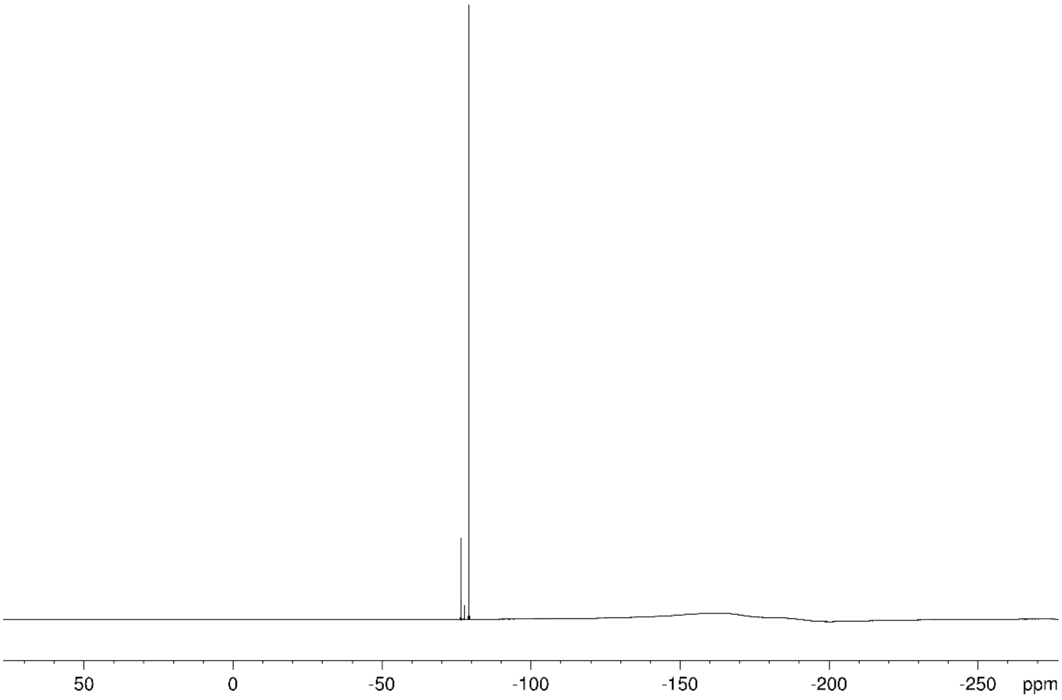

Figure S8. ^{19}F NMR spectrum of **3a** in CH_2Cl_2/C_6D_6 at 298 K.

c. [IDipp·BH$_2$·NH$_2$^{i}Pr][OTf] (3b)

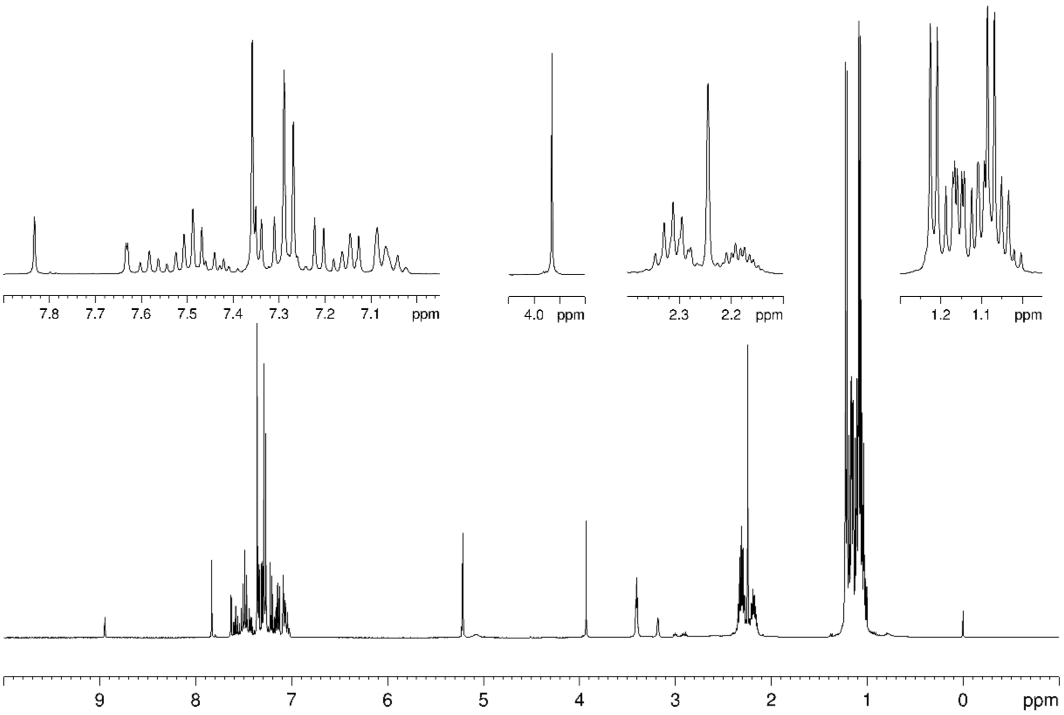

Figure S9. ^{1}H NMR spectrum of **3b** in CD_2Cl_2 at 298 K.

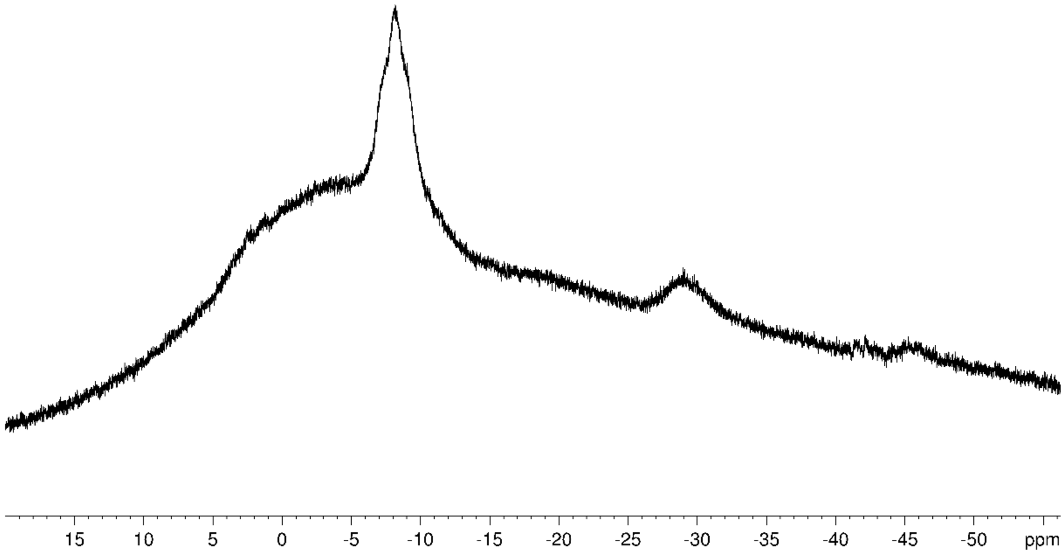

Figure S10. ^{11}B NMR spectrum of **3b** in CD_2Cl_2 at 298 K.

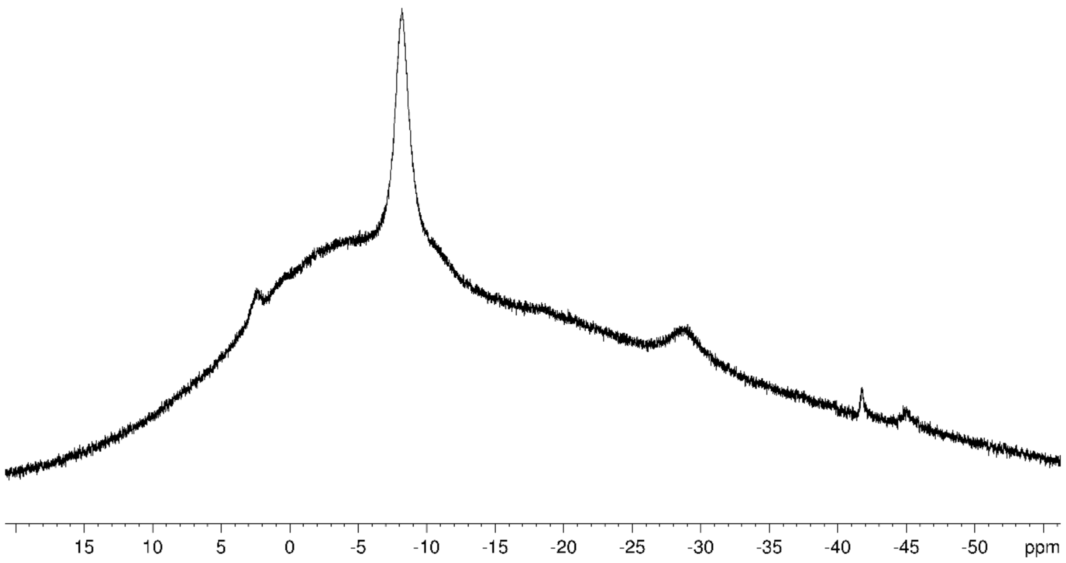

Figure S11. $^{11}B\{^{1}H\}$ NMR spectrum of **3b** in CD_2Cl_2 at 298 K.

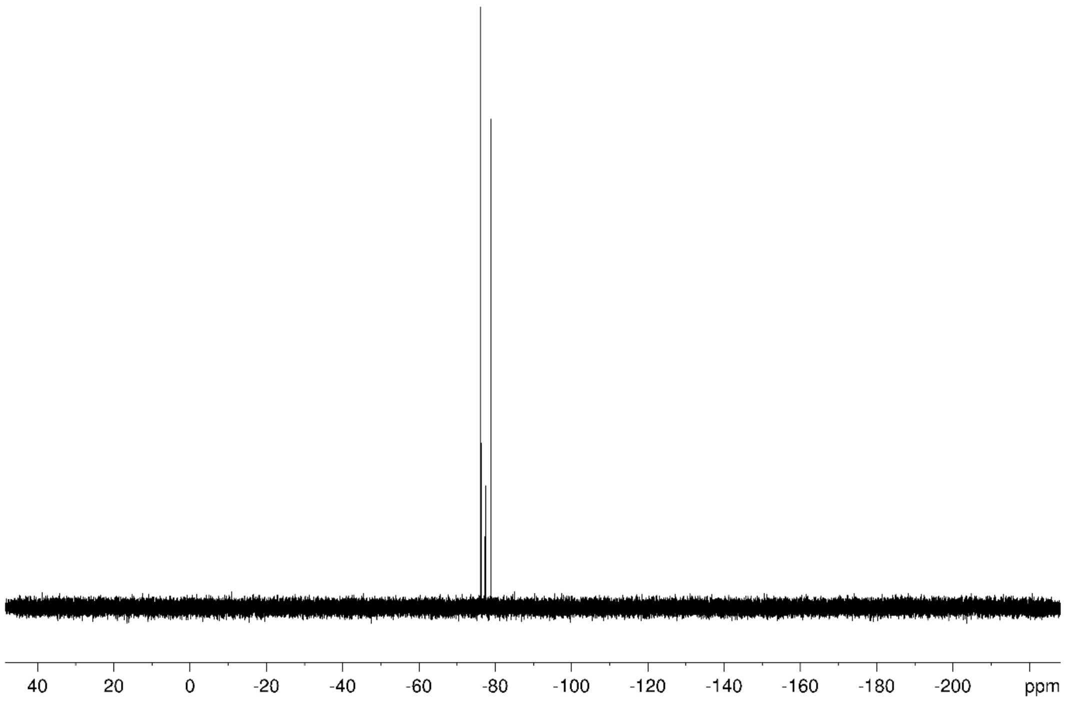

Figure S12. ^{19}F NMR spectrum of **3b** in CD_2Cl_2 at 298 K.

d. [IDipp·BH_2·$NH_2{}^tBu$][OTf] (3c)

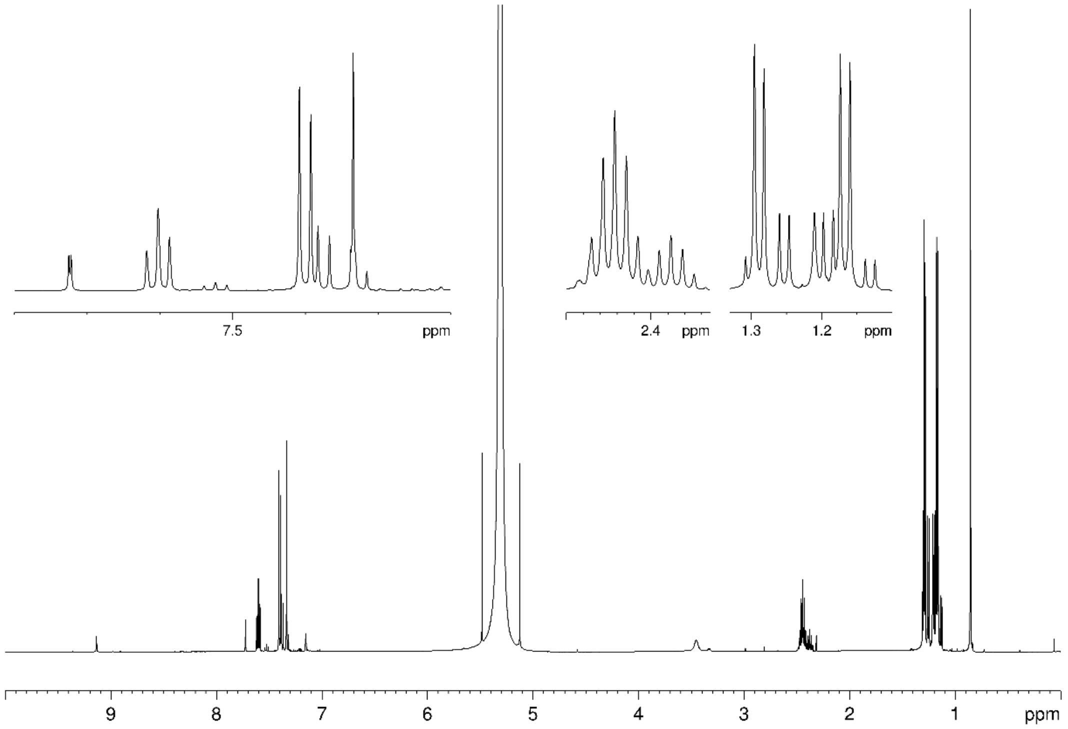

Figure S13. ^{1}H NMR spectrum of **3c** in CH_2Cl_2/C_6D_6 at 298 K.

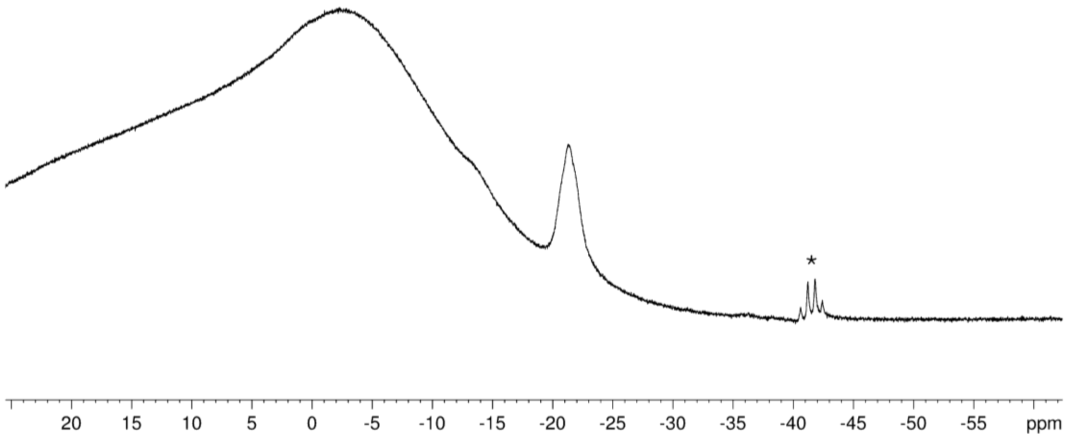

Figure S14. ^{11}B NMR spectrum of **3c** in CH_2Cl_2/C_6D_6 at 298 K. * - IDippBH$_3$.

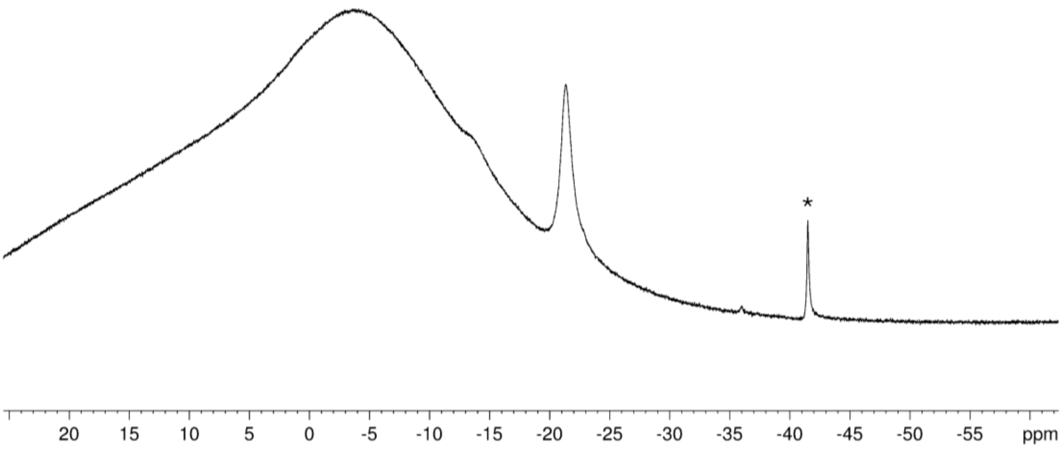

Figure S15. $^{11}B\{^{1}H\}$ NMR spectrum of **3c** in CH_2Cl_2/C_6D_6 at 298 K. * - $IDippBH_3$

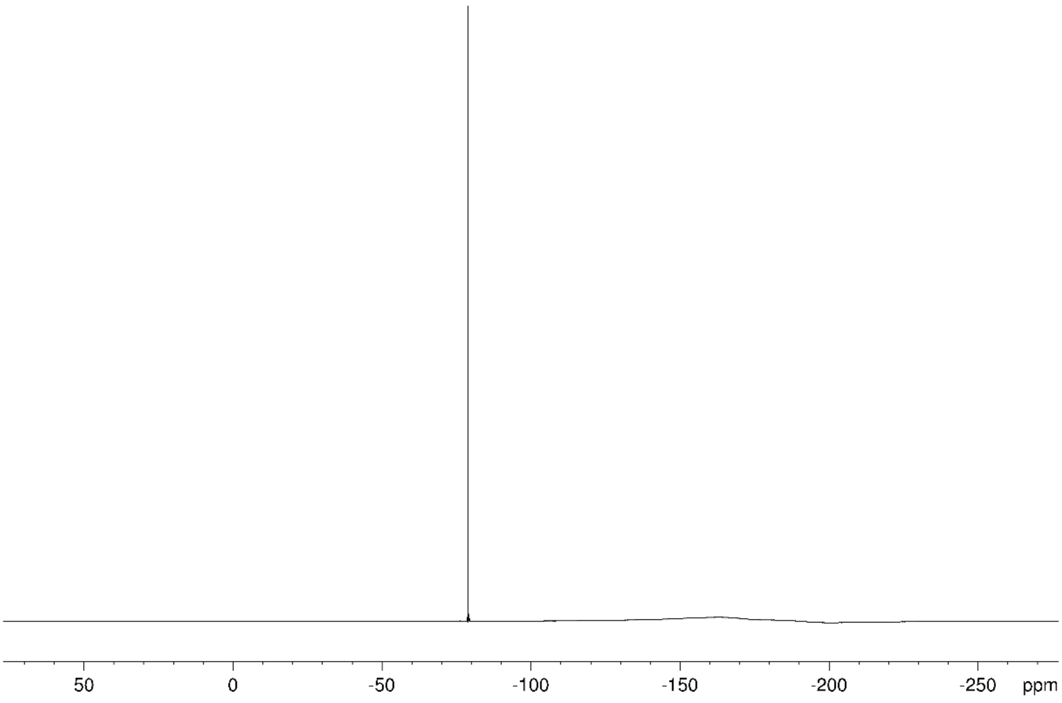

Figure S16. ^{19}F NMR spectrum of **3c** in CH_2Cl_2/C_6D_6 at 298 K.

e. [IDipp·GeH$_2$BH$_2$·Py][OTf] (5)

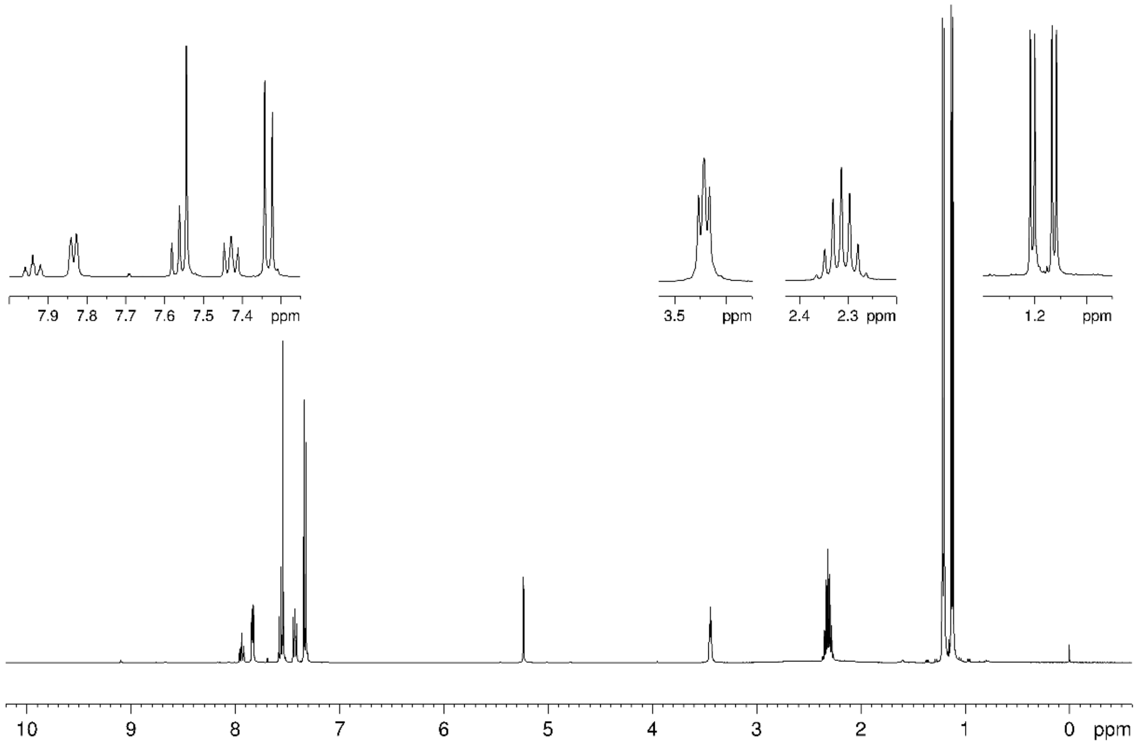

Figure S17. ^{1}H NMR spectrum of **5** in CD_2Cl_2 at 298 K.

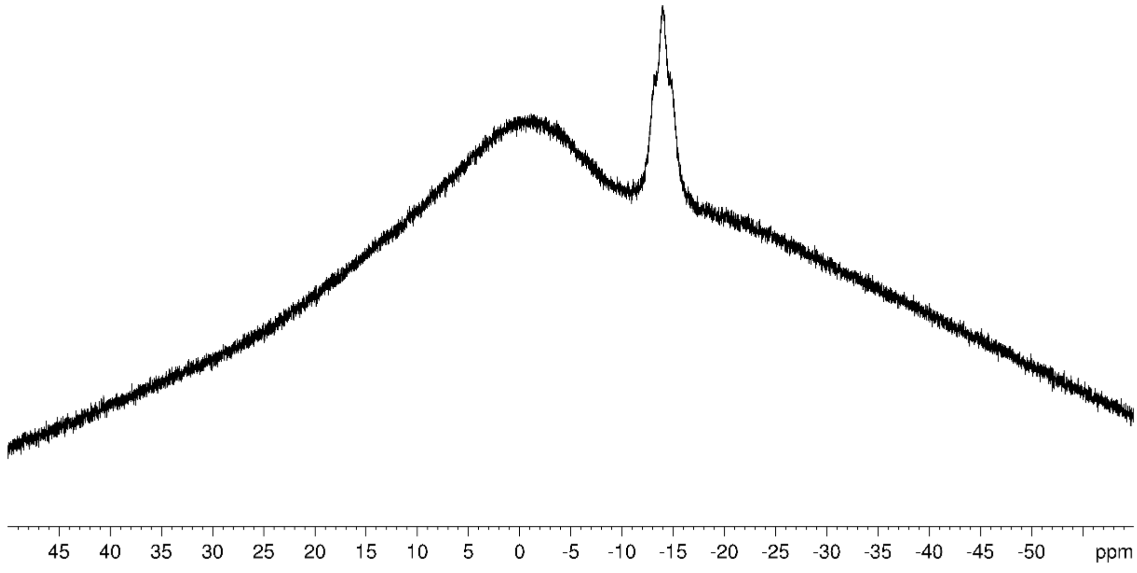

Figure S18. ^{11}B NMR spectrum of **5** in CD_2Cl_2 at 298 K.

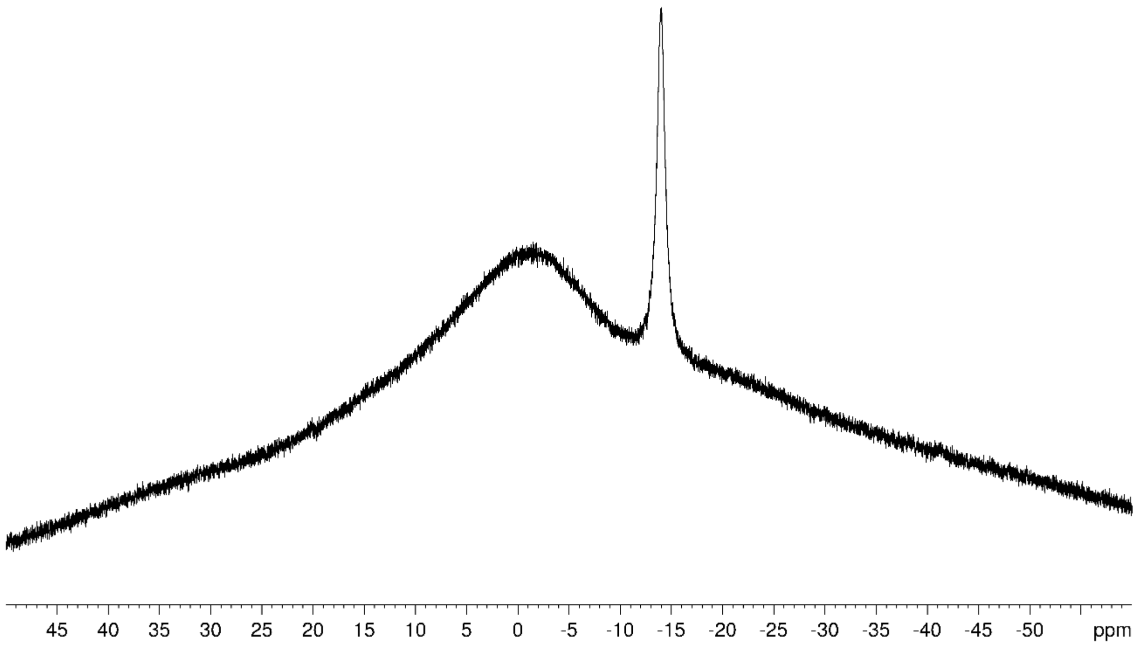

Figure S19. $^{11}B\{^{1}H\}$ NMR spectrum of **5** in CD_2Cl_2 at 298 K.

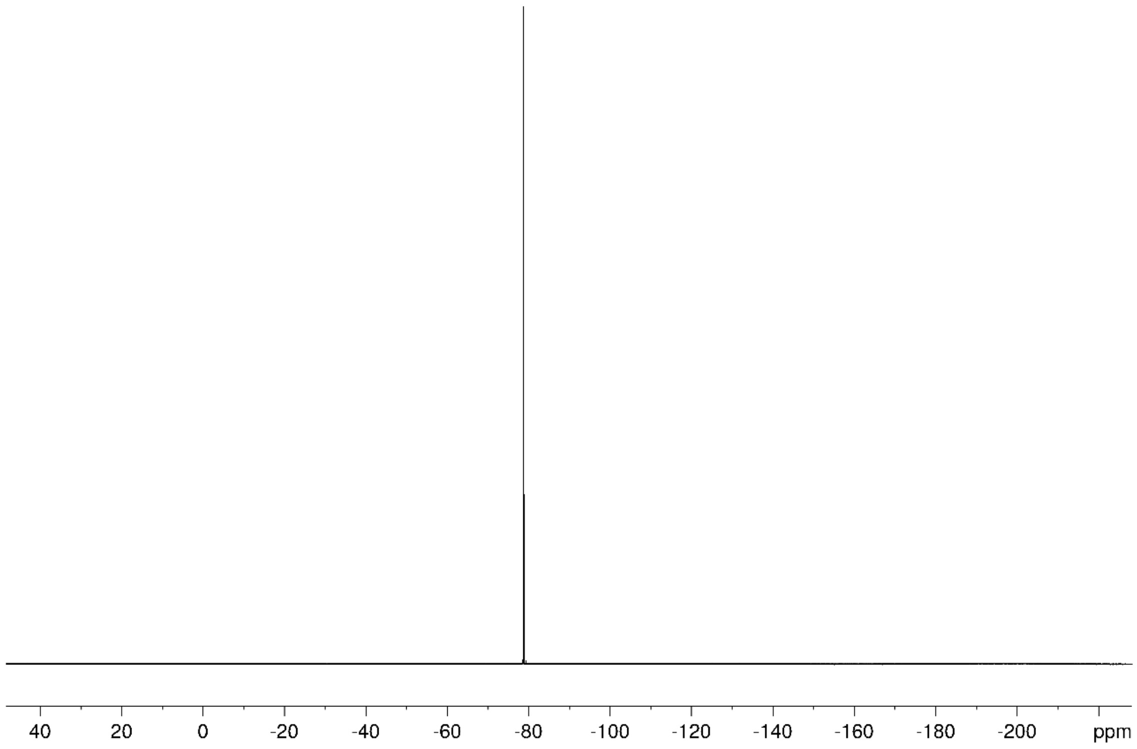

Figure S20. ^{19}F NMR spectrum of **5** in CD_2Cl_2 at 298 K.

f. [IDipp·GeH_2BH_2·DMAP][OTf] (6)

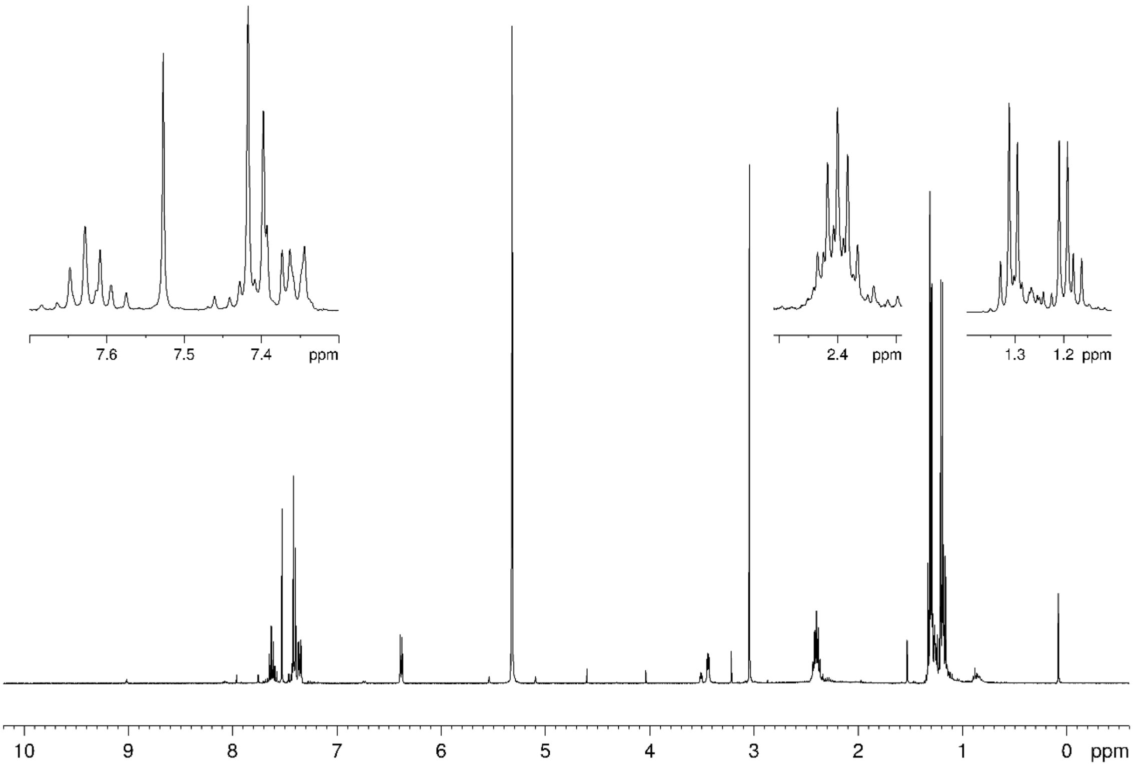

Figure S21. ^{1}H NMR spectrum of **6** in CD_2Cl_2 at 298 K.

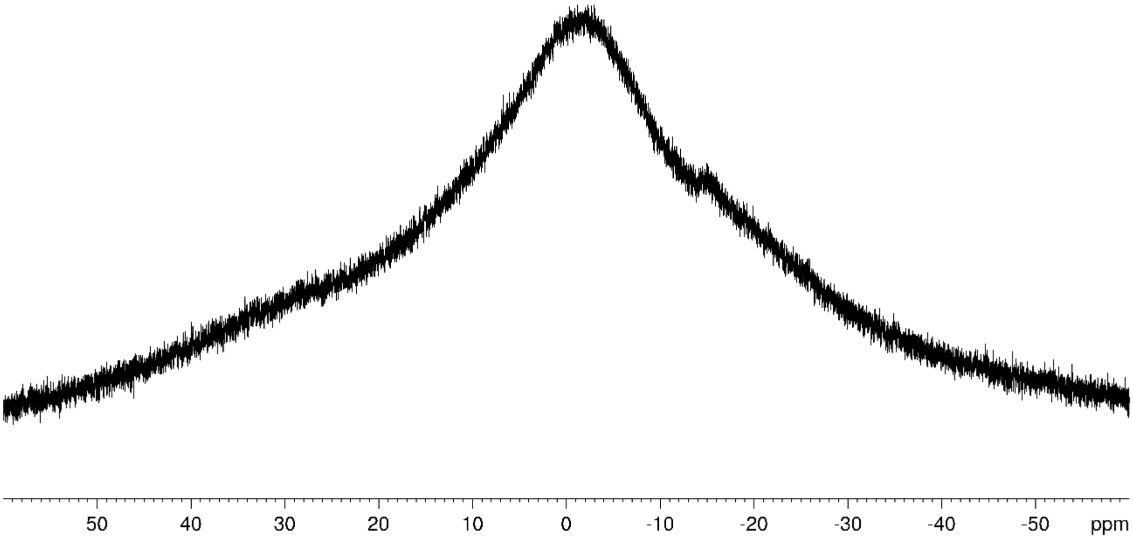

Figure S22. ^{11}B NMR spectrum of **6** in CD_2Cl_2 at 298 K.

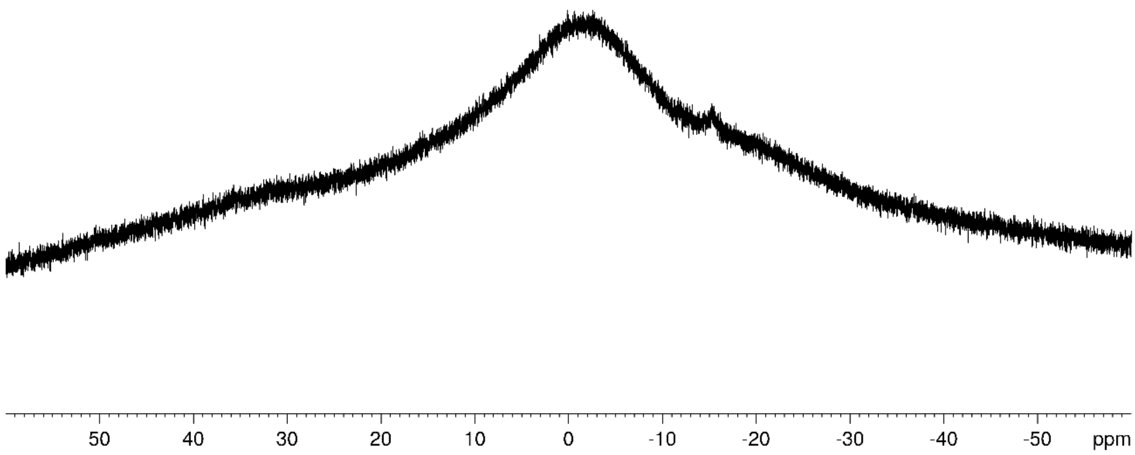

Figure S23. $^{11}B\{^{1}H\}$ NMR spectrum of **6** in CD_2Cl_2 at 298 K.

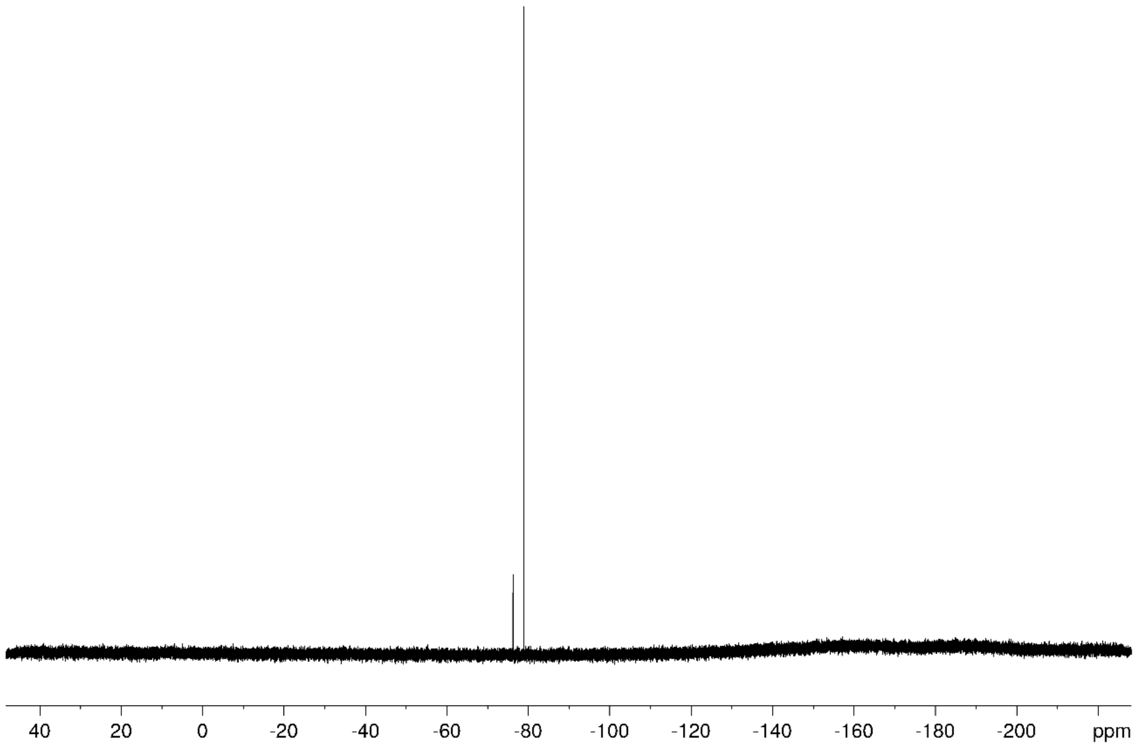

Figure S24. ^{19}F NMR spectrum of **6** in CD_2Cl_2 at 298 K.

g. [IDipp·GeH_2BH_2·bipy·BH_2GeH_2·IDipp][OTf]$_2$ (7)

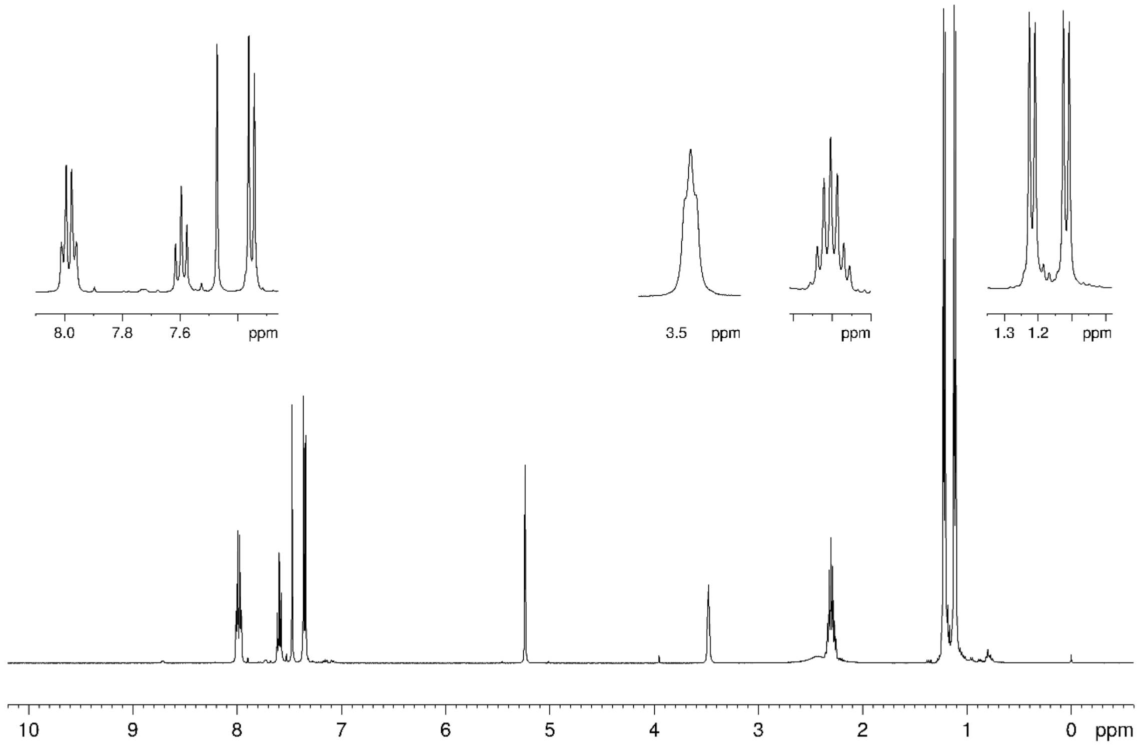

Figure S25. ^{1}H NMR spectrum of **7** in CD_2Cl_2 at 298 K.

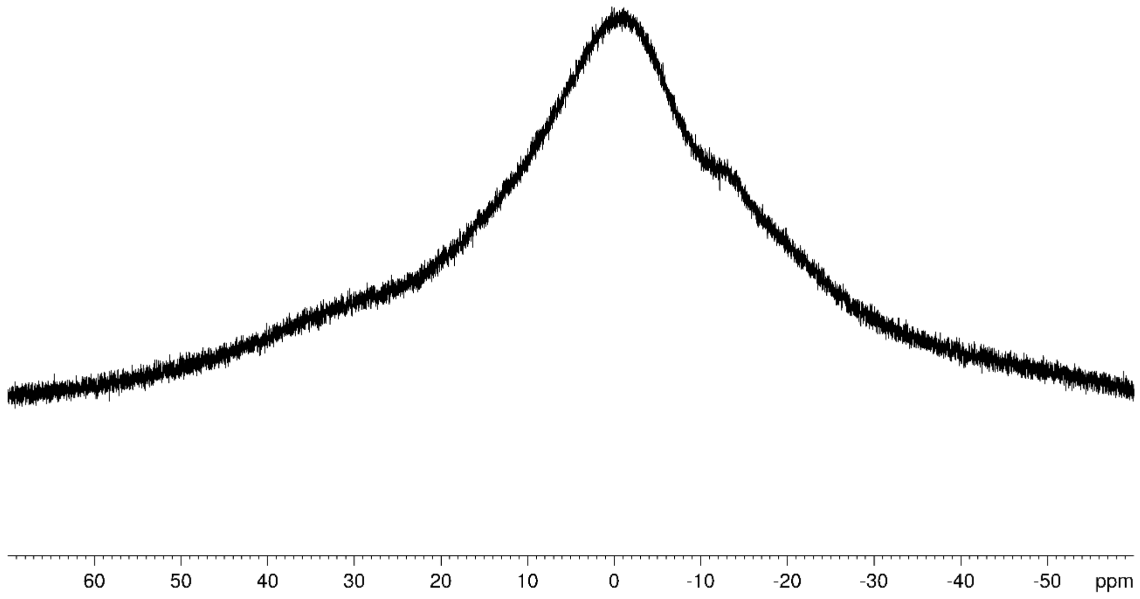

Figure S26. ^{11}B NMR spectrum of **7** in CD_2Cl_2 at 298 K.

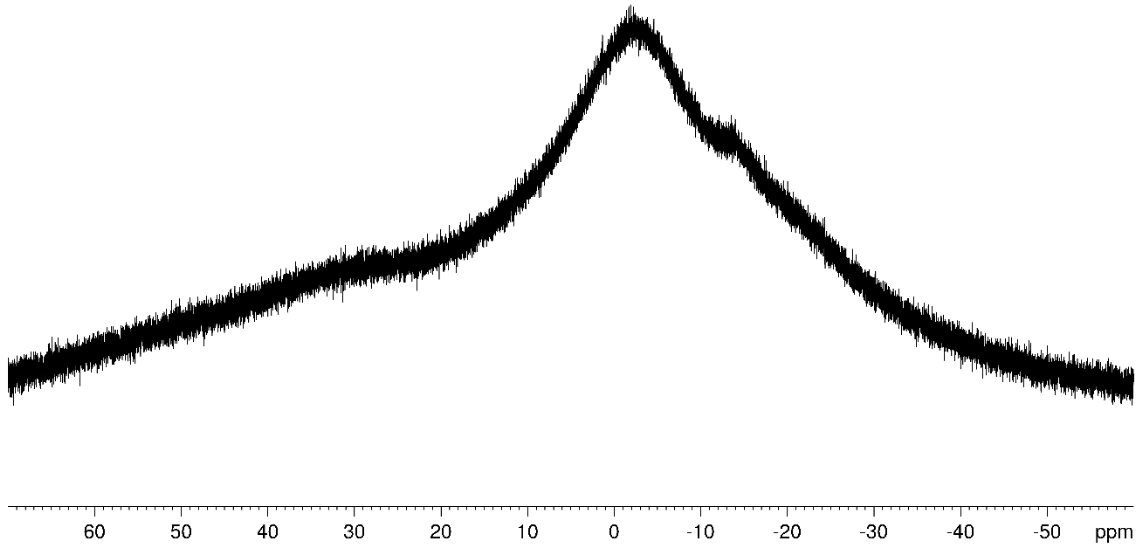

Figure S27. $^{11}B\{^{1}H\}$ NMR spectrum of **7** in CD_2Cl_2 at 298 K.

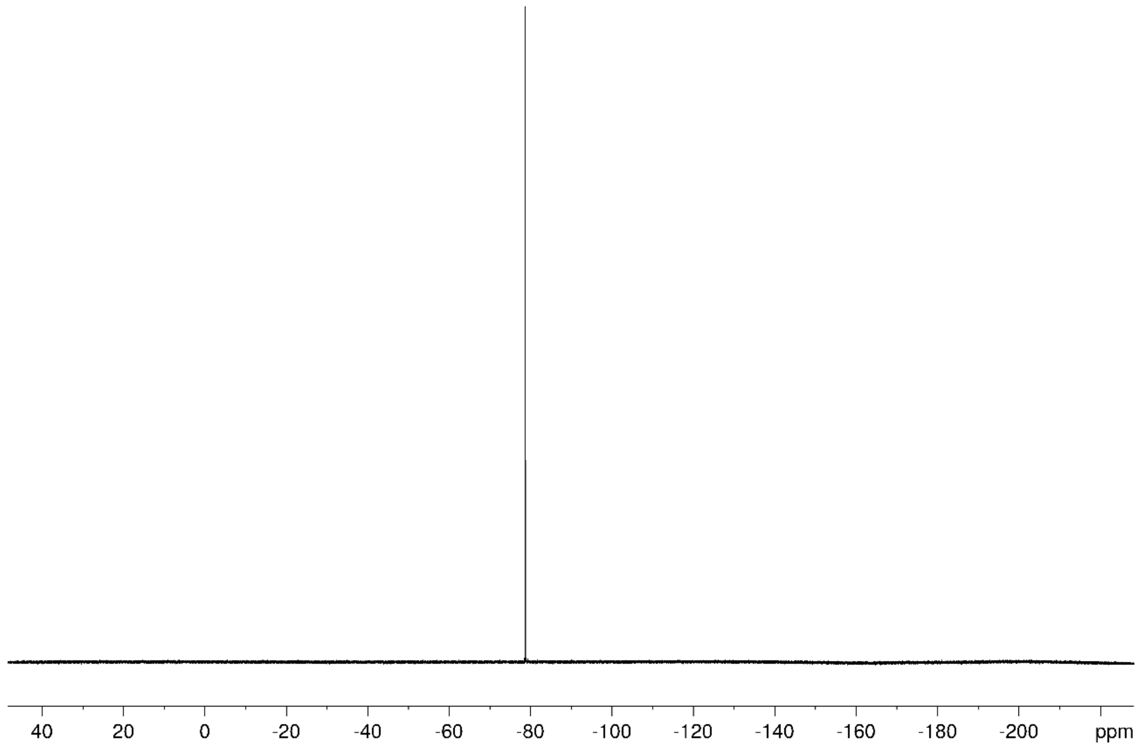

Figure S28. ^{19}F NMR spectrum of **7** in CD_2Cl_2 at 298 K.

h. [IDipp·GeH_2BH_2·dppe·BH_2GeH_2·IDipp]$[OTf]_2$ (8)

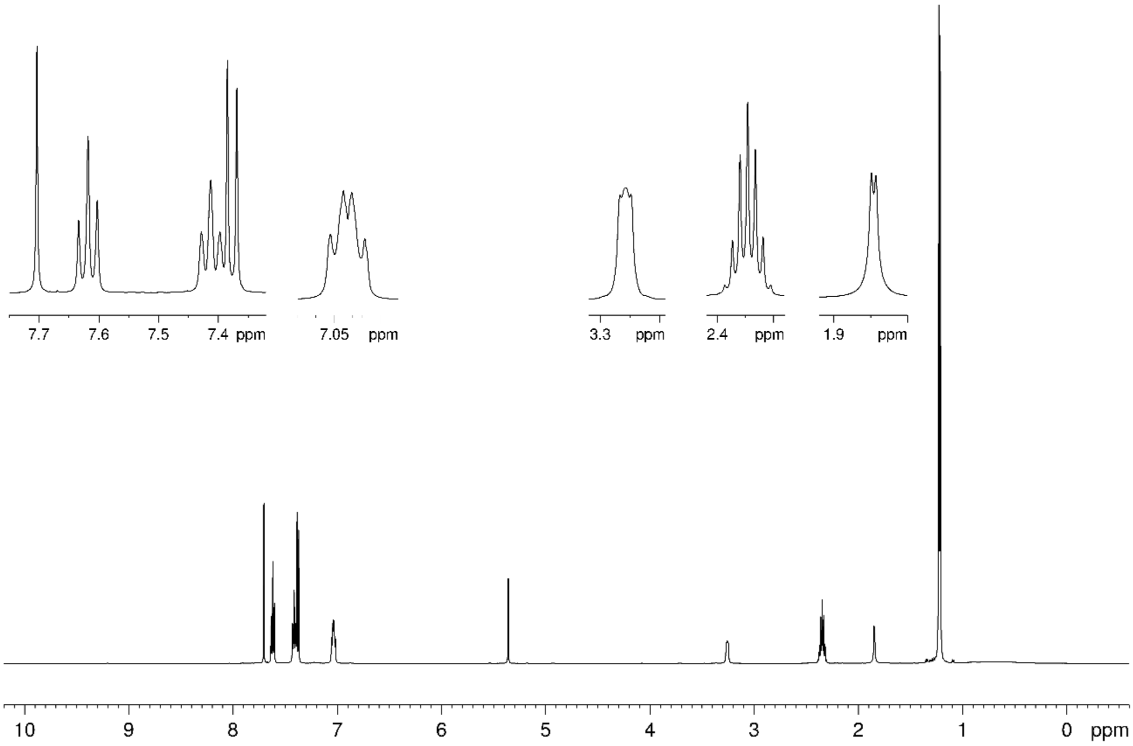

Figure S29. ^{1}H NMR spectrum of **8** in CD_2Cl_2 at 298 K.

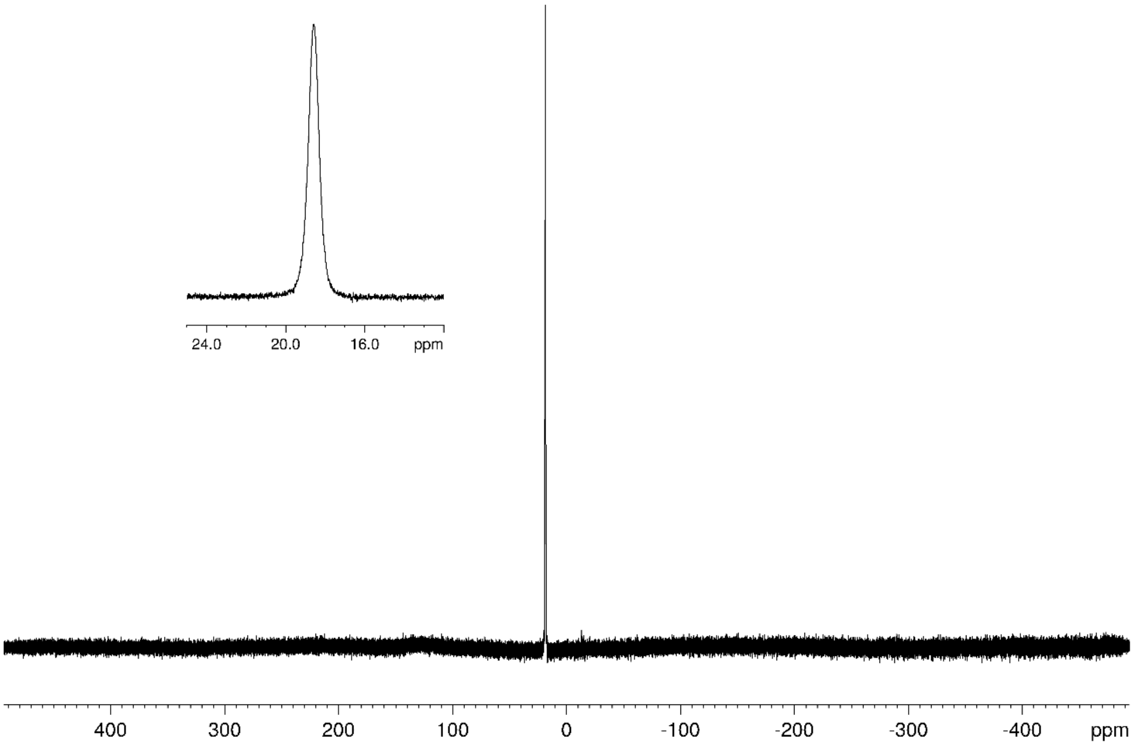

Figure S30. ^{31}P NMR spectrum of **8** in CD_2Cl_2 at 298 K.

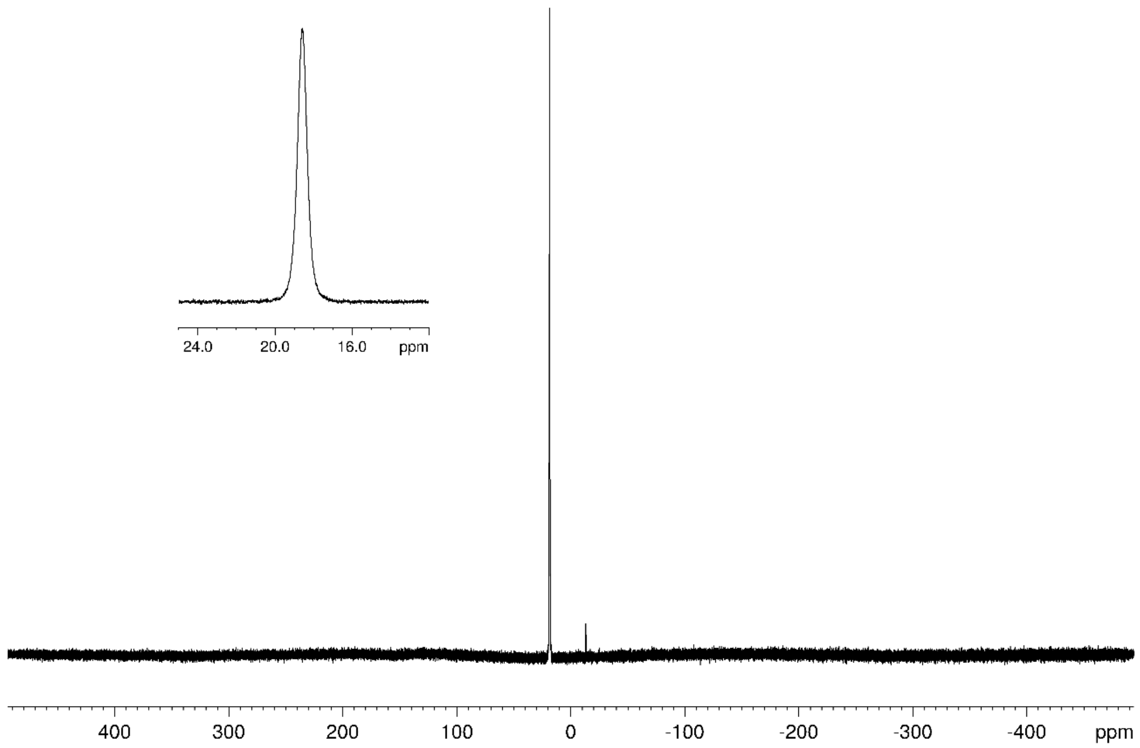

Figure S31. $^{31}P\{^{1}H\}$ NMR spectrum of **8** in CD_2Cl_2 at 298 K.

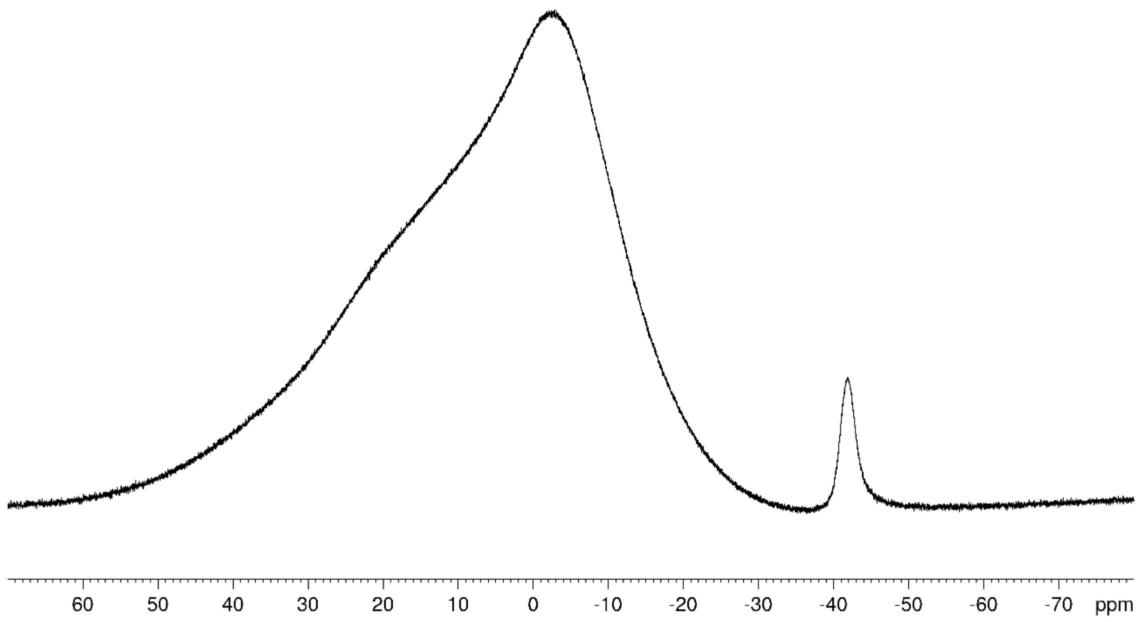

Figure S32. ^{11}B NMR spectrum of **8** in CD_2Cl_2 at 298 K.

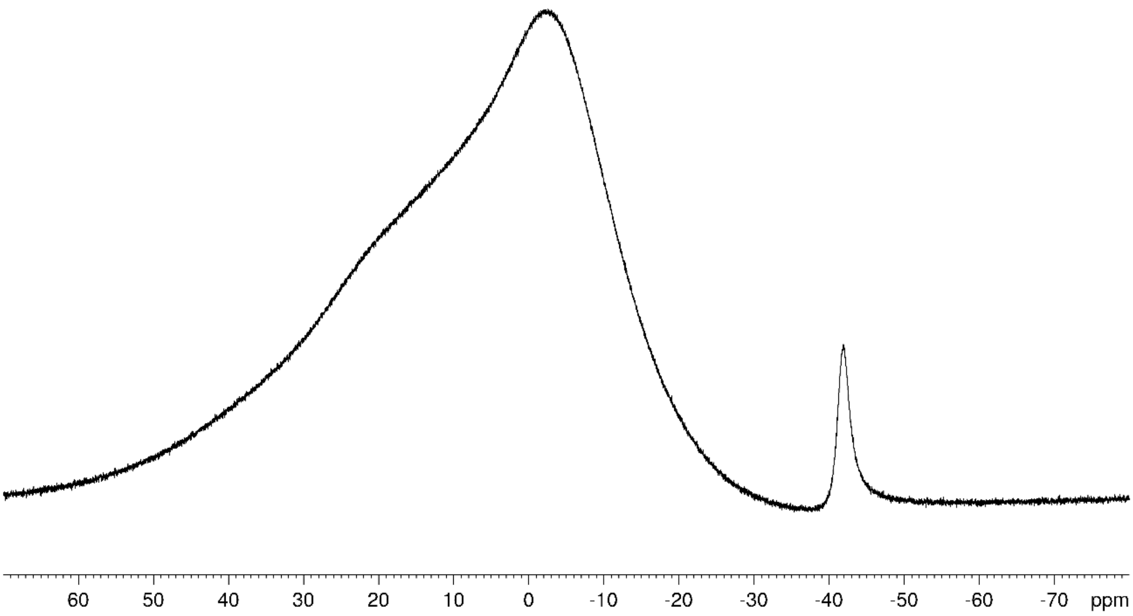

Figure S33. $^{11}B\{^{1}H\}$ NMR spectrum of **8** in CD_2Cl_2 at 298 K.

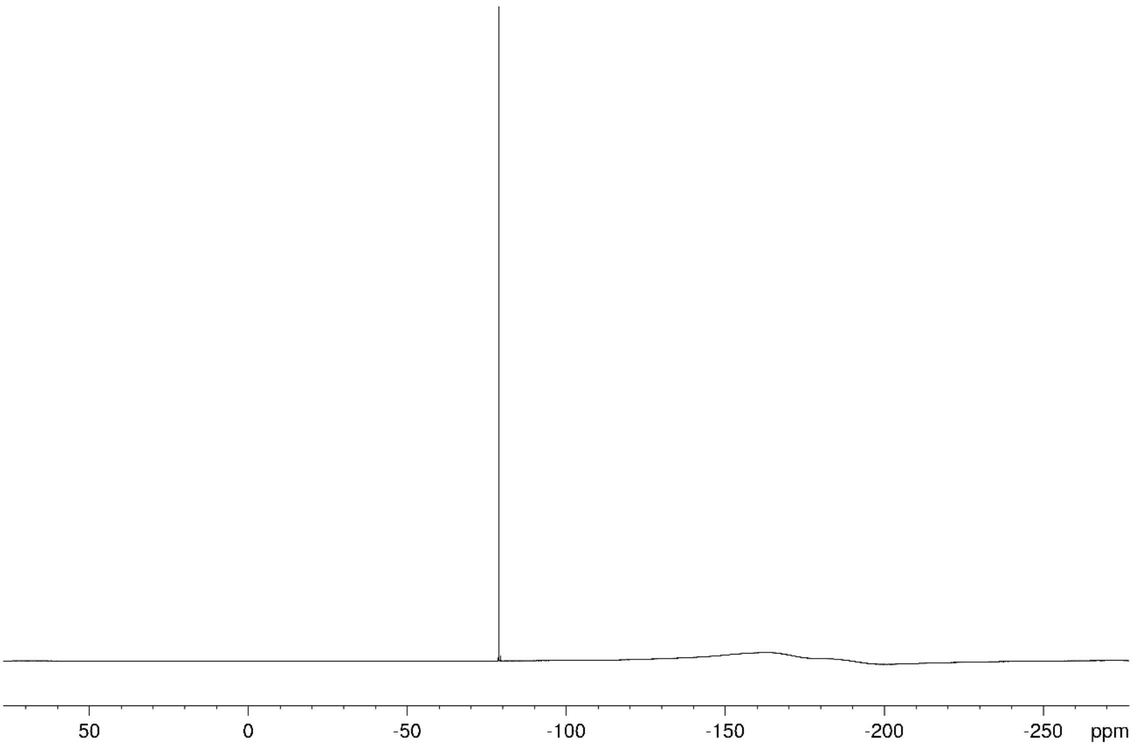

Figure S34. ^{19}F NMR spectrum of **8** in CD_2Cl_2 at 298 K.

i. $[Cp_2Mo_2(CO)_4(\eta^2\text{-}P_2)\cdot BH_2GeH_2\cdot IDipp][OTf]$ (9)

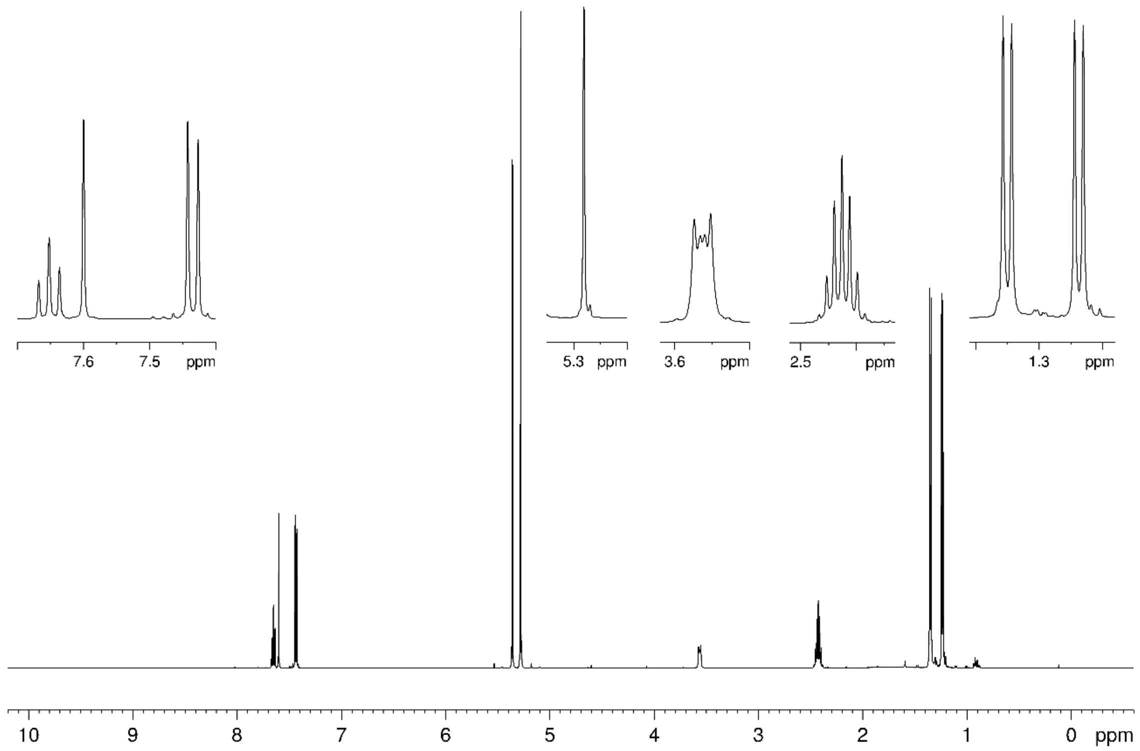

Figure S35. ^1H NMR spectrum of **9** in CD_2Cl_2 at 298 K.

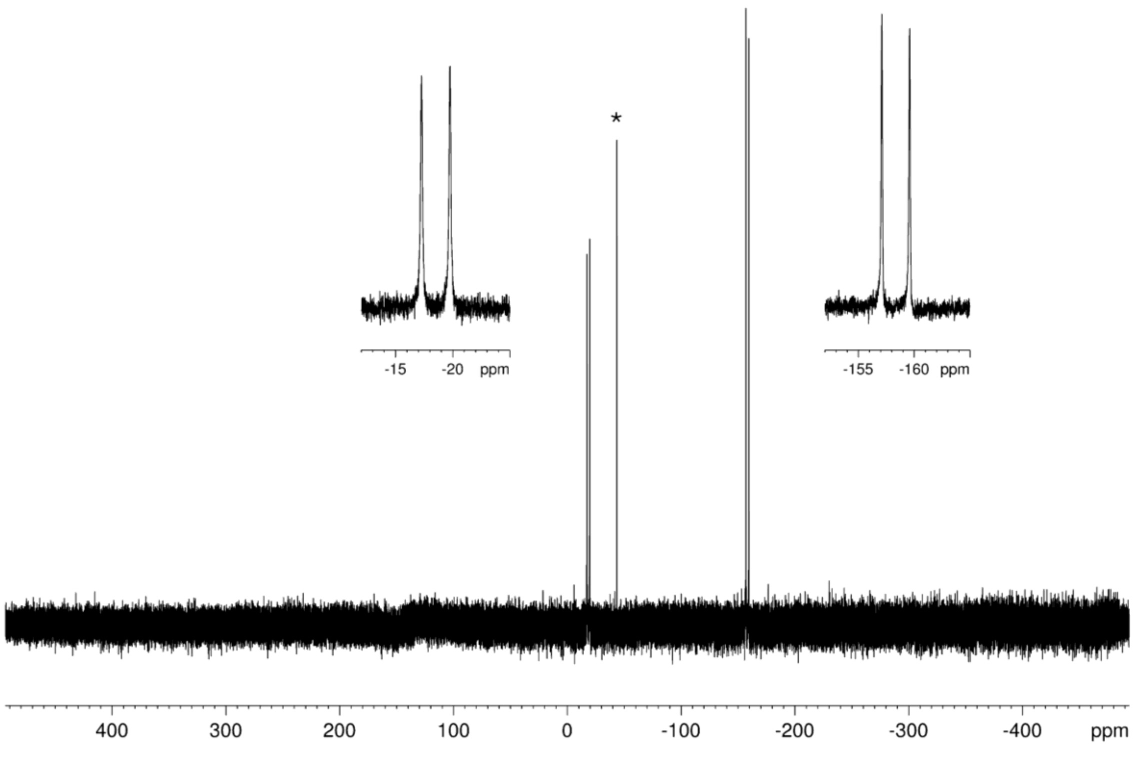

Figure S36. ^{31}P NMR spectrum of **9** in CD_2Cl_2 at 298 K. * - $Cp_2Mo_2(CO)_4(\eta^2\text{-}P_2)$.

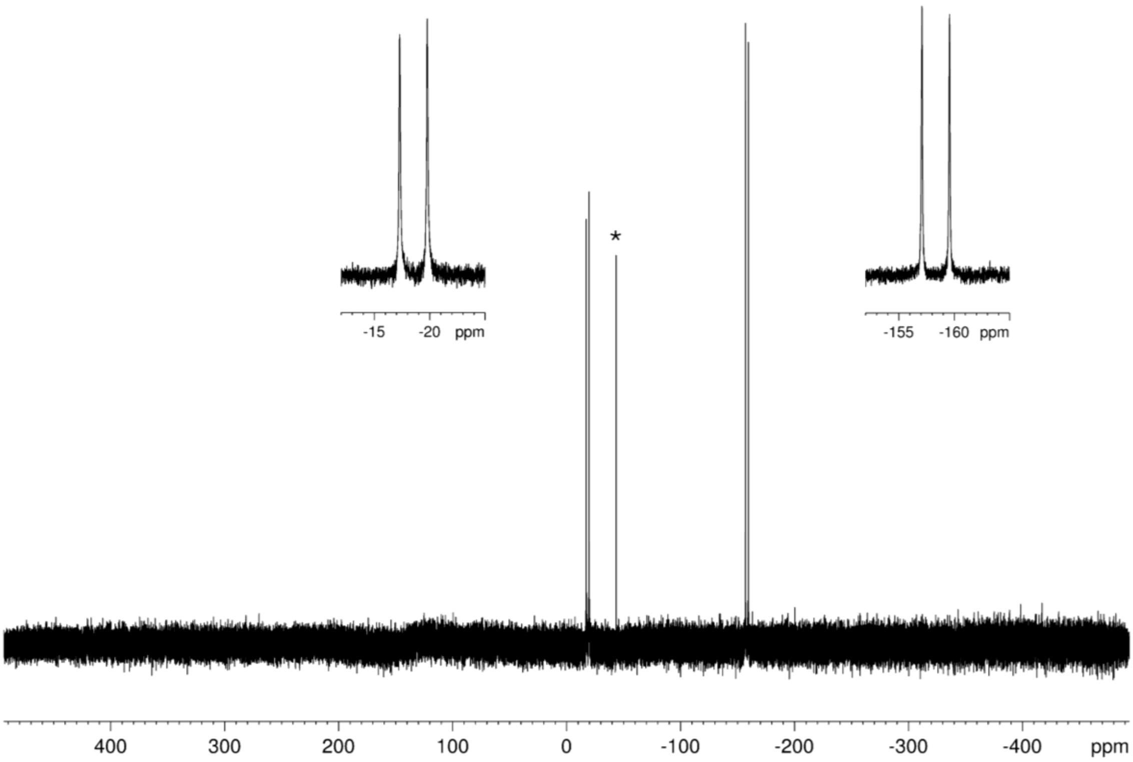

Figure S37. $^{31}P\{^{1}H\}$ NMR spectrum of **9** in CD_2Cl_2 at 298 K. * - $Cp_2Mo_2(CO)_4(\eta^2\text{-}P_2)$.

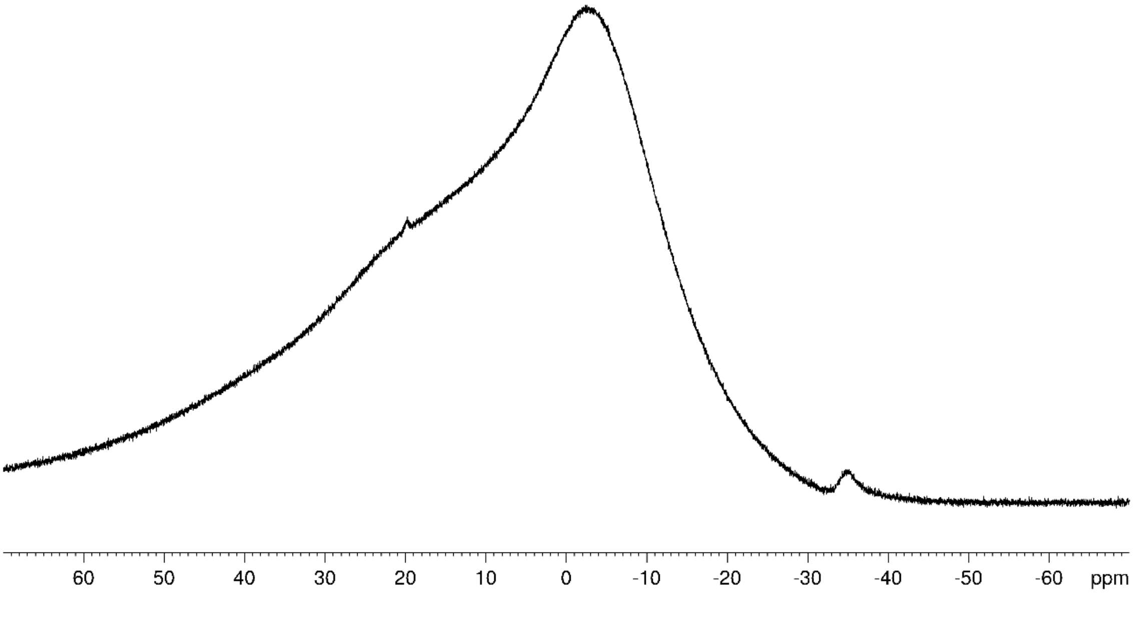

Figure S38. ^{11}B NMR spectrum of **9** in CD_2Cl_2 at 298 K.

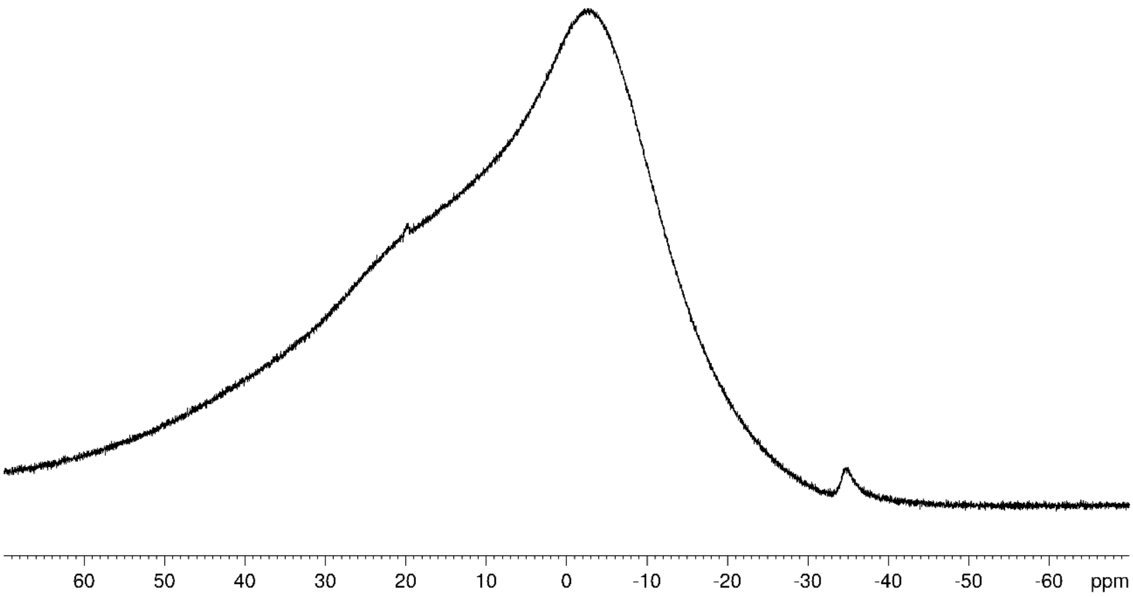

Figure S39. $^{11}B\{^{1}H\}$ NMR spectrum of **9** in CD_2Cl_2 at 298 K.

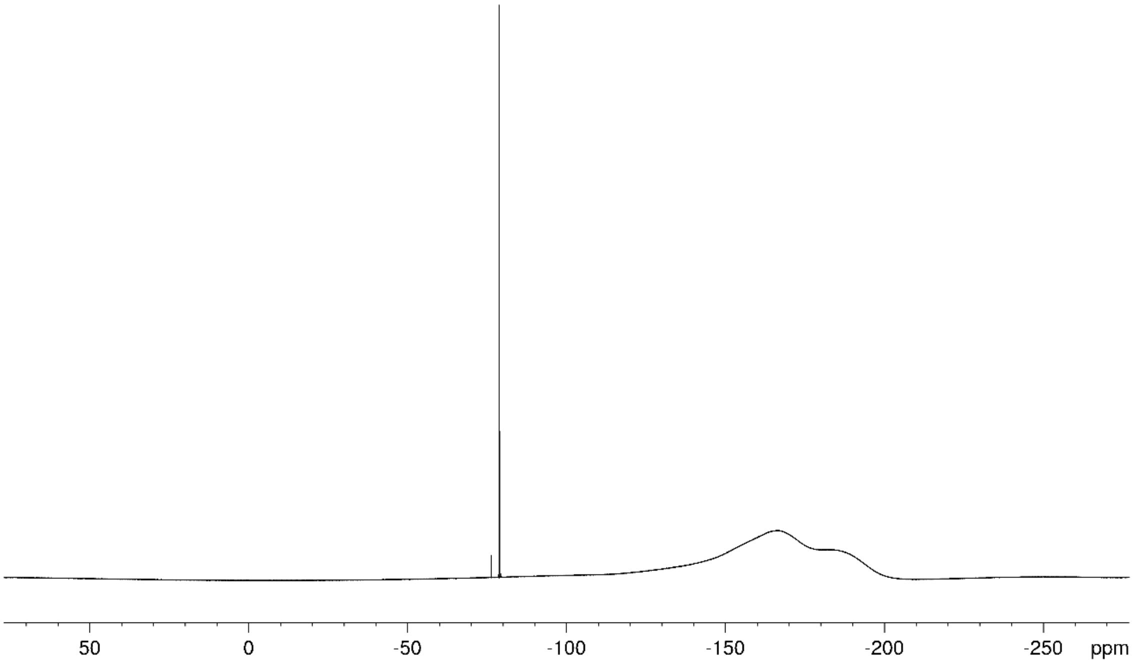

Figure S40. ^{19}F NMR spectrum of **9** in CD_2Cl_2 at 298 K.

j. Anion exchange experiments

i. [IDipp·GeH_2BH_2][TEF]

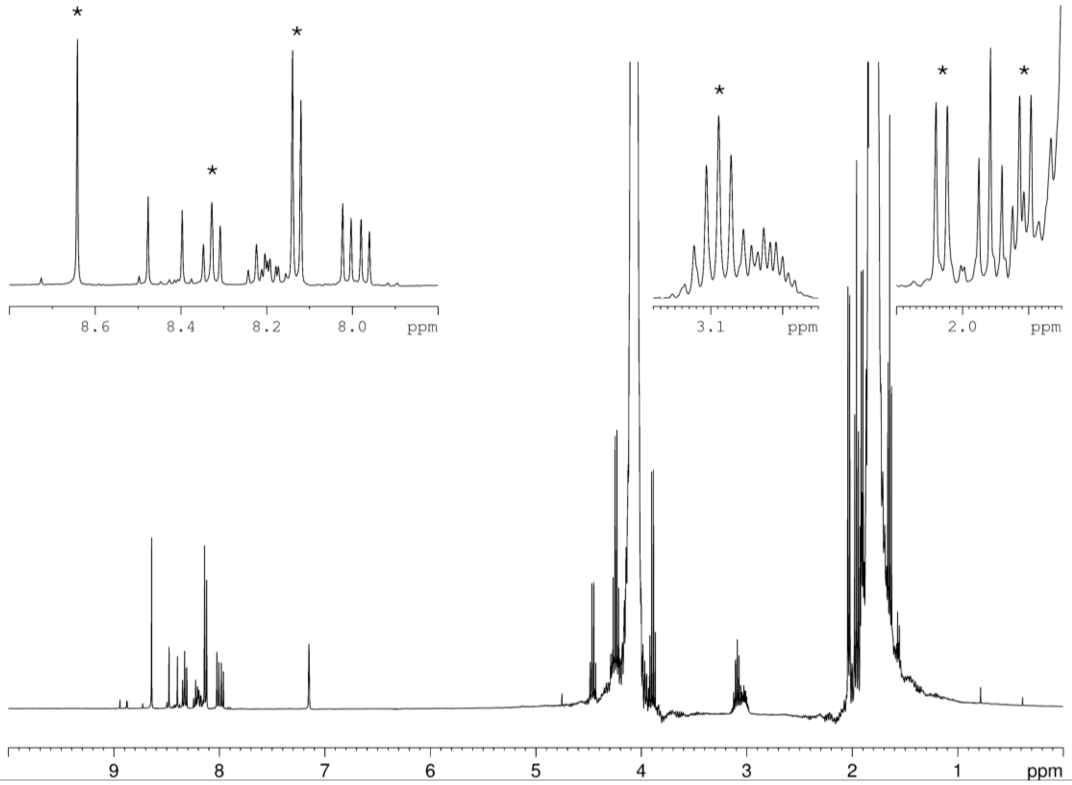

Figure S41. ^{1}H NMR spectrum of [IDipp·GeH_2BH_2][TEF] in Et_2O/C_6D_6 at 298 K. * - product signals.

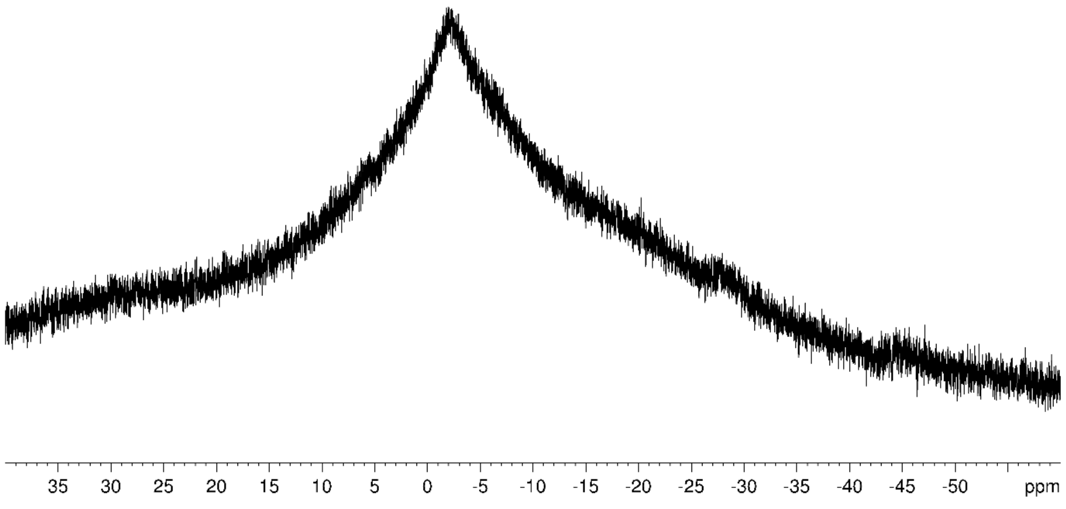

Figure S42. ^{11}B NMR spectrum of [IDipp·GeH_2BH_2][TEF] in Et_2O/C_6D_6 at 298 K.

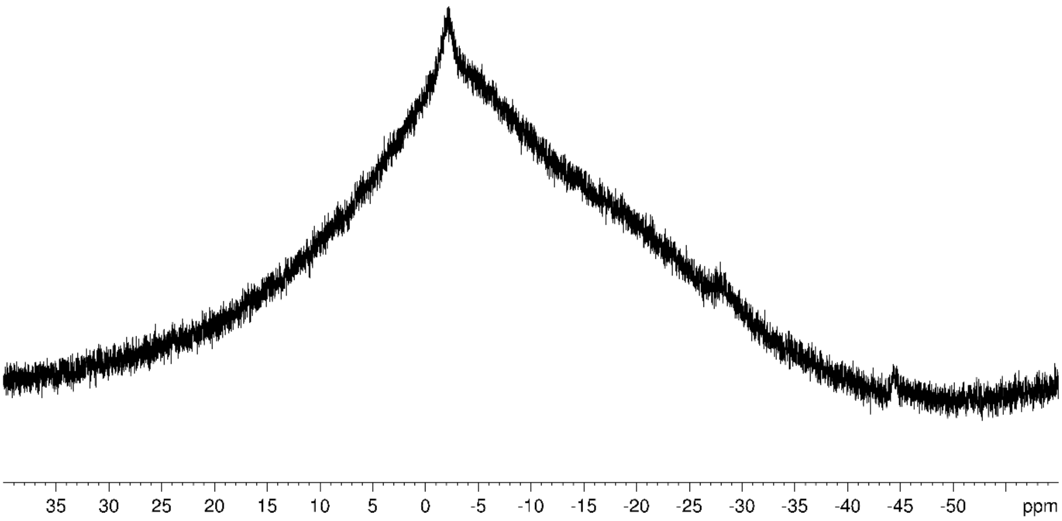

Figure S43. $^{11}B\{^{1}H\}$ NMR spectrum of [IDipp·GeH_2BH_2][TEF] in Et_2O/C_6D_6 at 298 K.

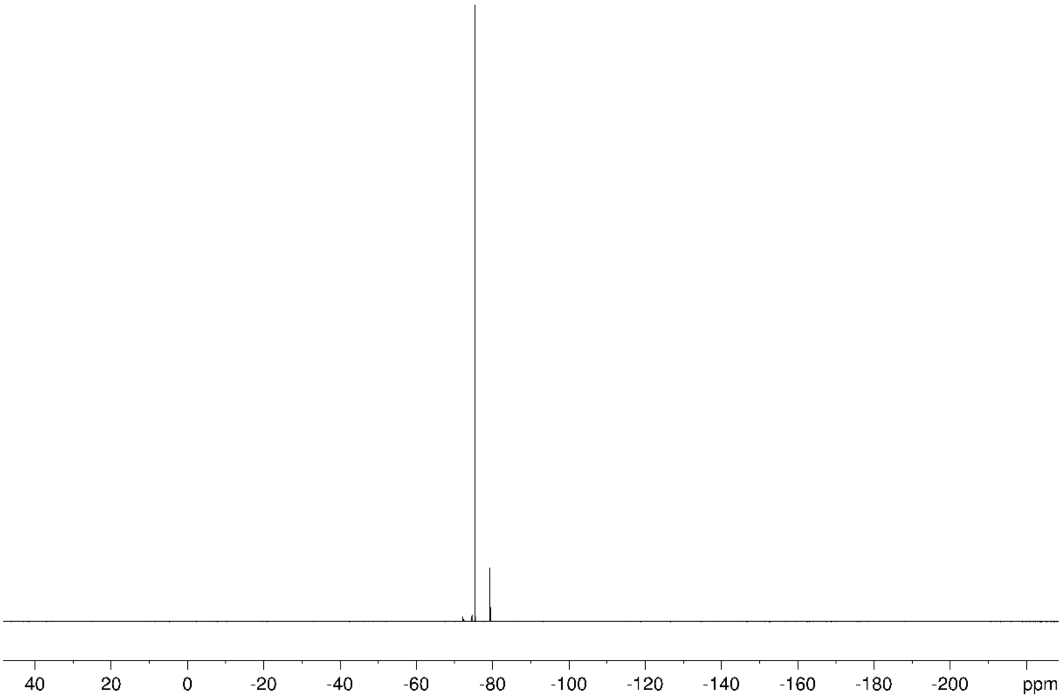

Figure S44. ^{19}F NMR spectrum of [IDipp·GeH_2BH_2][TEF] in Et_2O/C_6D_6 at 298 K.

ii. Reaction of [IDipp·GeH_2BH_2][TEF] with $NHEt_2$

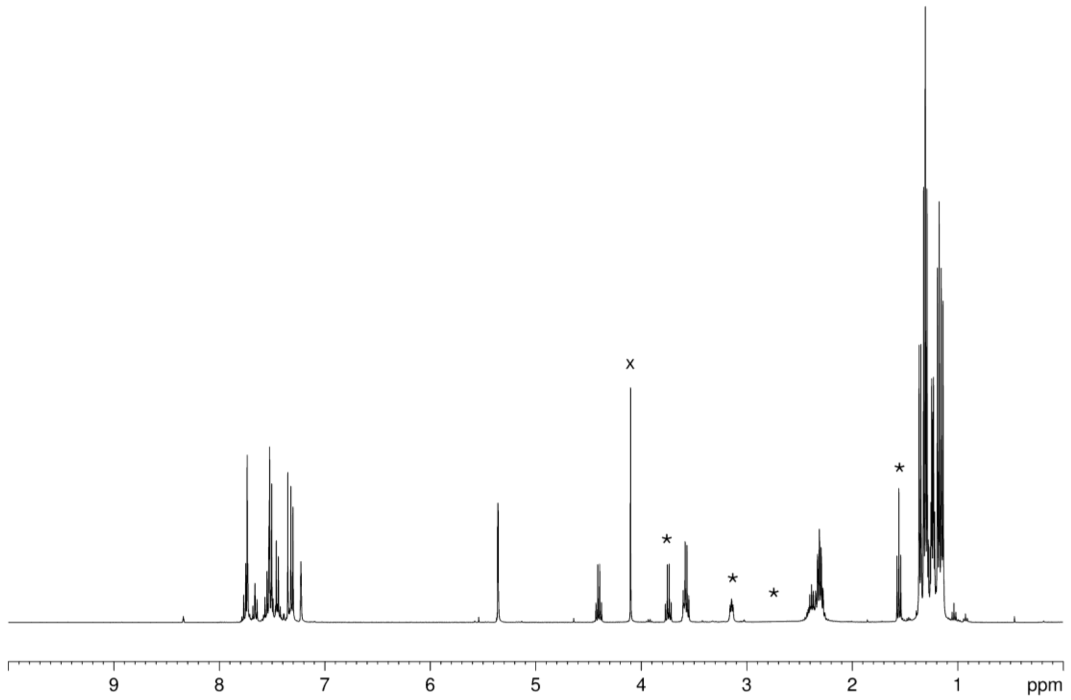

Figure S45. ^{1}H NMR spectrum of [IDipp·GeH_2BH_2·OEt_2][TEF] in CD_2Cl_2 at 298 K. * - product signals, x - [IDipp·GeH_3][OTf] signal.

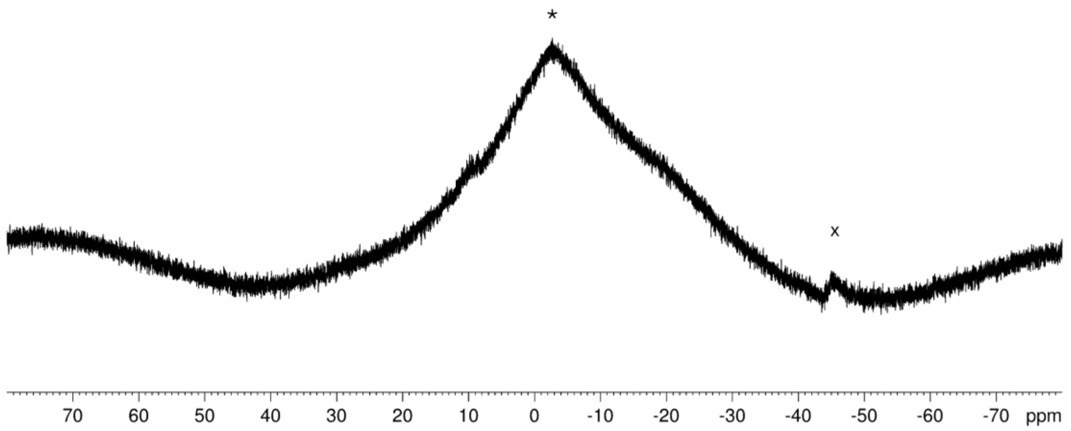

Figure S46. ^{11}B NMR spectrum of [IDipp·GeH_2BH_2·OEt_2][TEF] in CD_2Cl_2 at 298 K. * - product signals, x - IDipp·BH_3 signal.

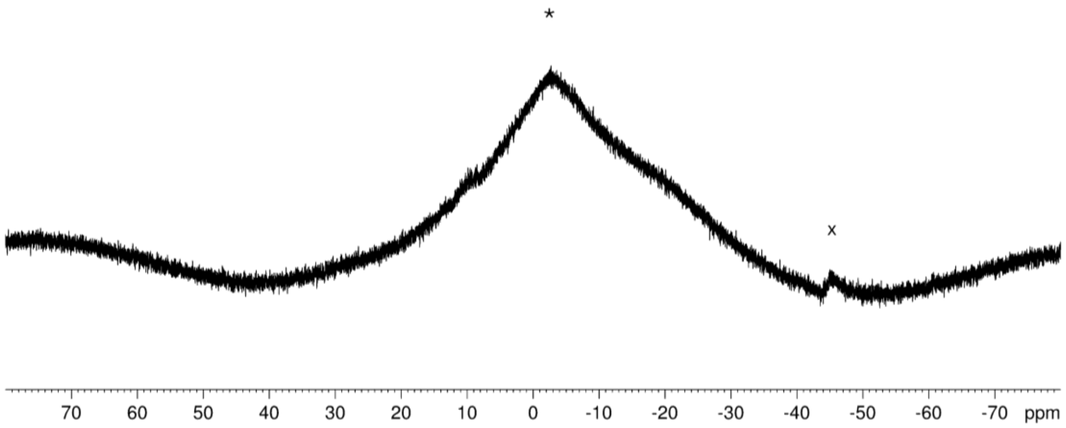

Figure S47. $^{11}B\{^1H\}$ NMR spectrum of [IDipp·GeH_2BH_2·OEt_2][TEF] in CD_2Cl_2 at 298 K. * - product signals, x - IDipp·BH_3 signal.

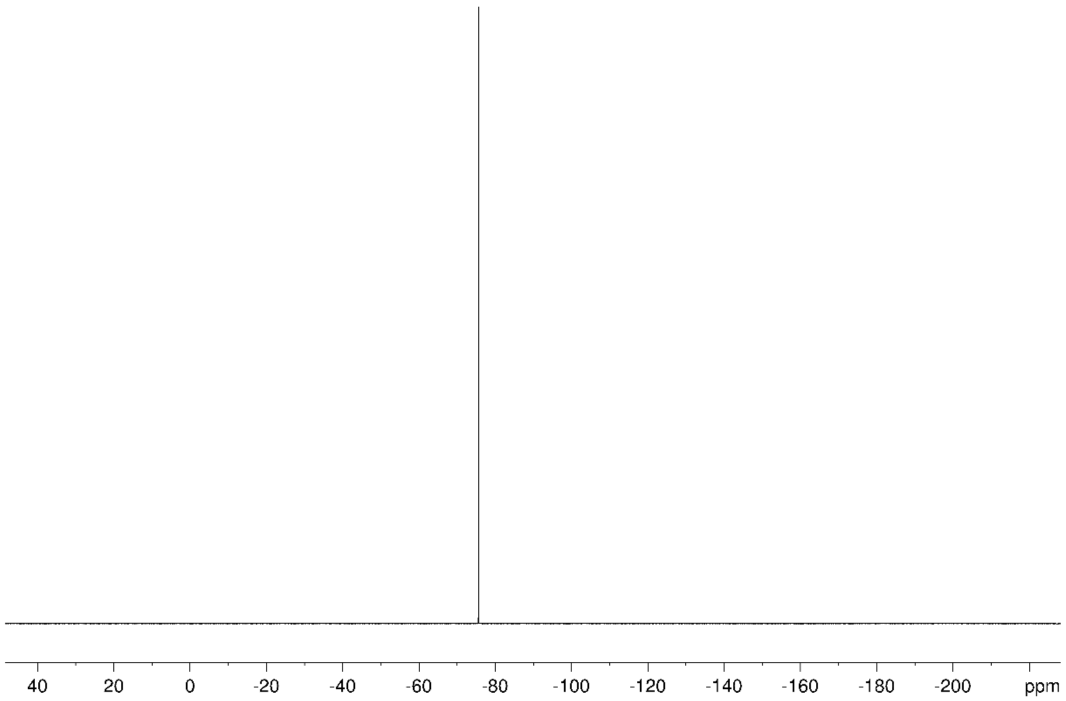

Figure S48. ^{19}F NMR spectrum of [IDipp·GeH_2BH_2·OEt_2][TEF] in CD_2Cl_2 at 298 K.

iii. [IDipp·GeH_2BH_2][BAr^F]

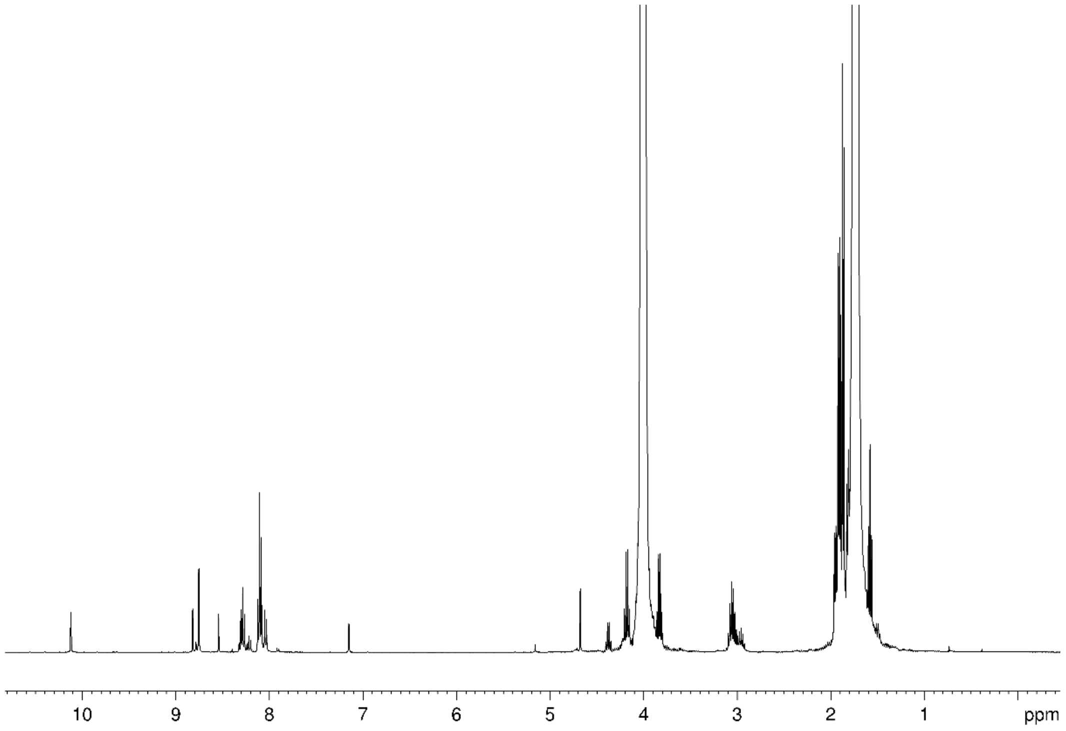

Figure S49. ^{1}H NMR spectrum of [IDipp·GeH_2BH_2][BAr^F] in Et_2O/C_6D_6 at 298 K.

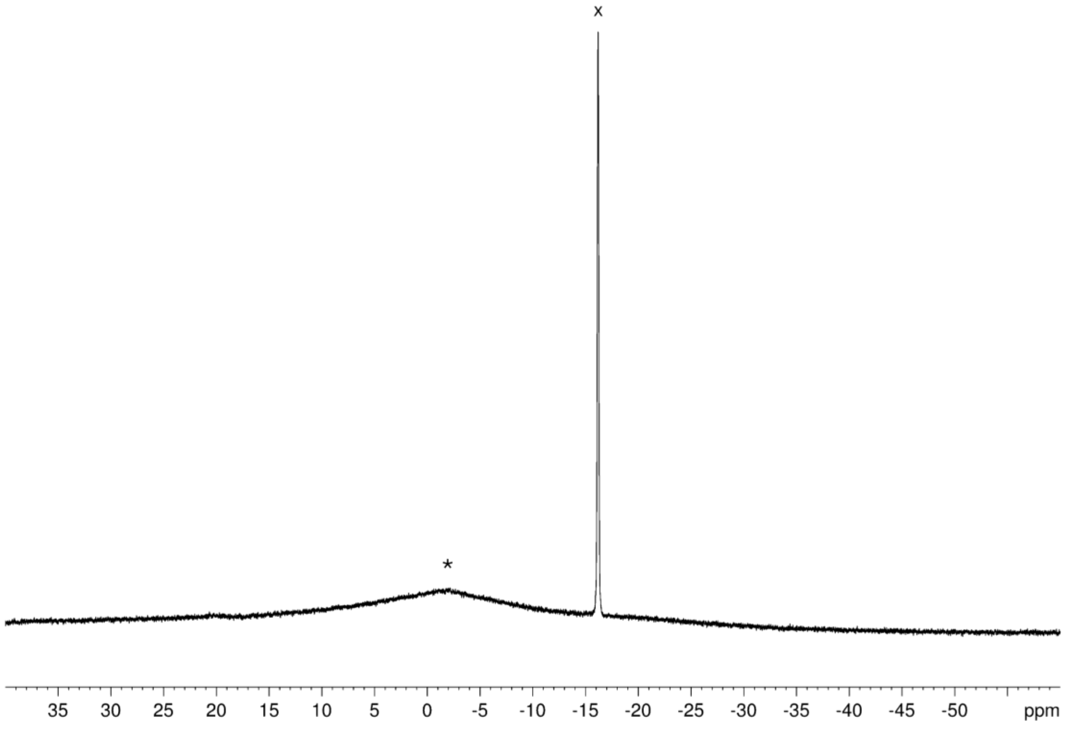

Figure S50. ^{11}B NMR spectrum of [IDipp·GeH_2BH_2][BAr^F] in Et_2O/C_6D_6 at 298 K. * - BH_2 signal, x – BAr^F signal.

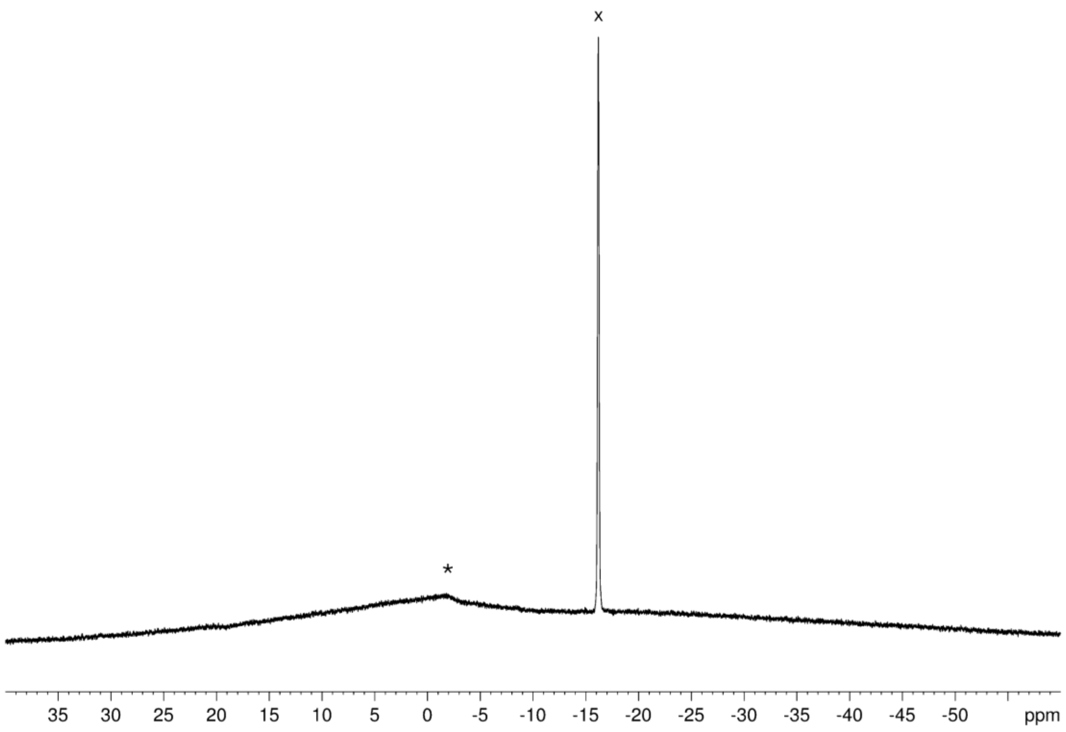

Figure S51. $^{11}B\{^{1}H\}$ NMR spectrum of [IDipp·GeH_2BH_2][BAr^F] in Et_2O/C_6D_6 at 298 K. * - BH_2 signal, x – BAr^F signal.

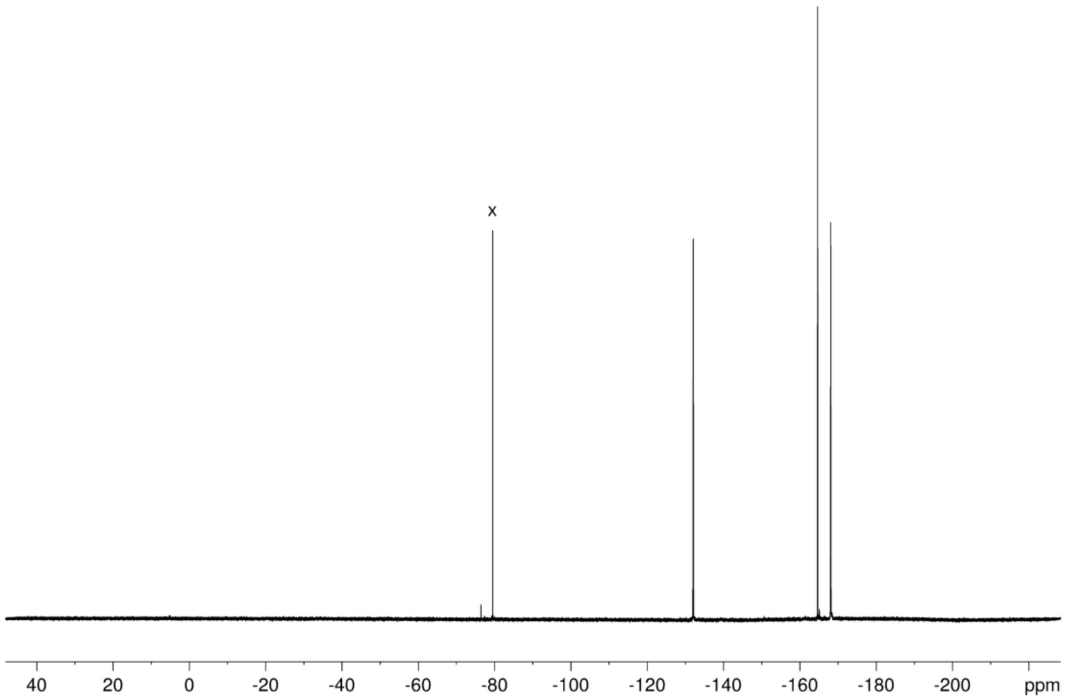

Figure S52. ^{19}F NMR spectrum of [IDipp·GeH_2BH_2][BAr^F] in Et_2O/C_6D_6 at 298 K. x – OTf signal.

iv. Reaction of [IDipp·GeH_2BH_2][BAr^F] with $NHEt_2$

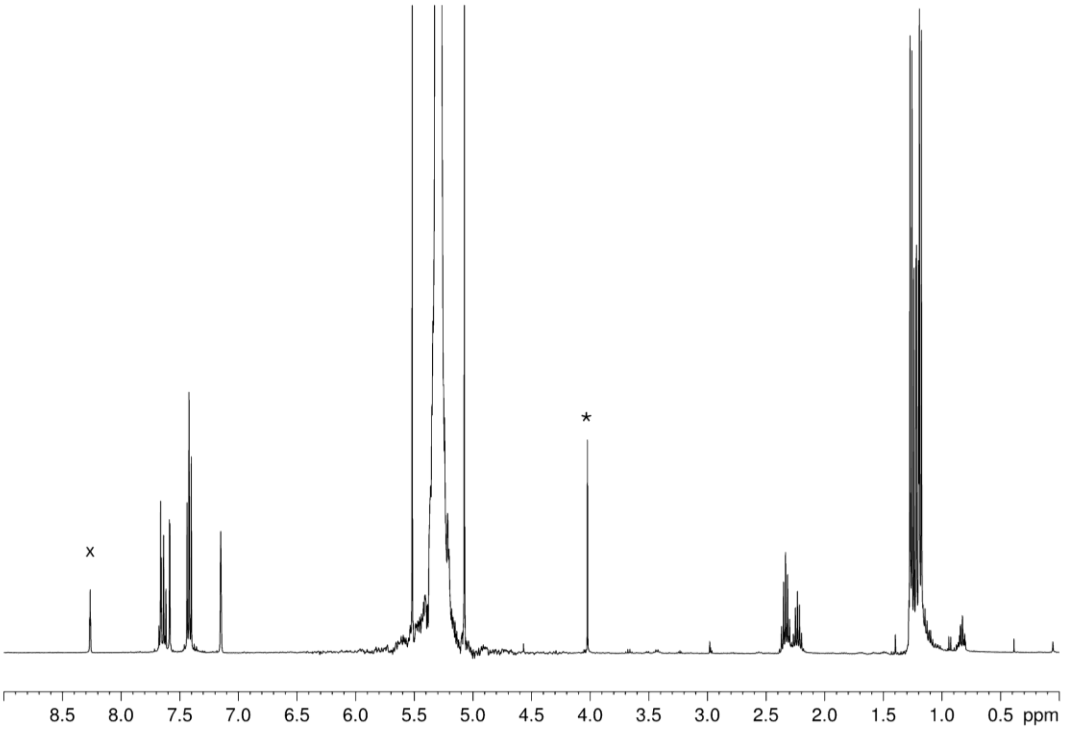

Figure S53. ^{1}H NMR spectrum of the reaction mixture of [IDipp·GeH_2BH_2][BAr^F] with $NHEt_2$ in CH_2Cl_2/C_6D_6 at 298 K. * - [IDipp·GeH_3][OTf] signal, x - [IDippH][OTf] signal.

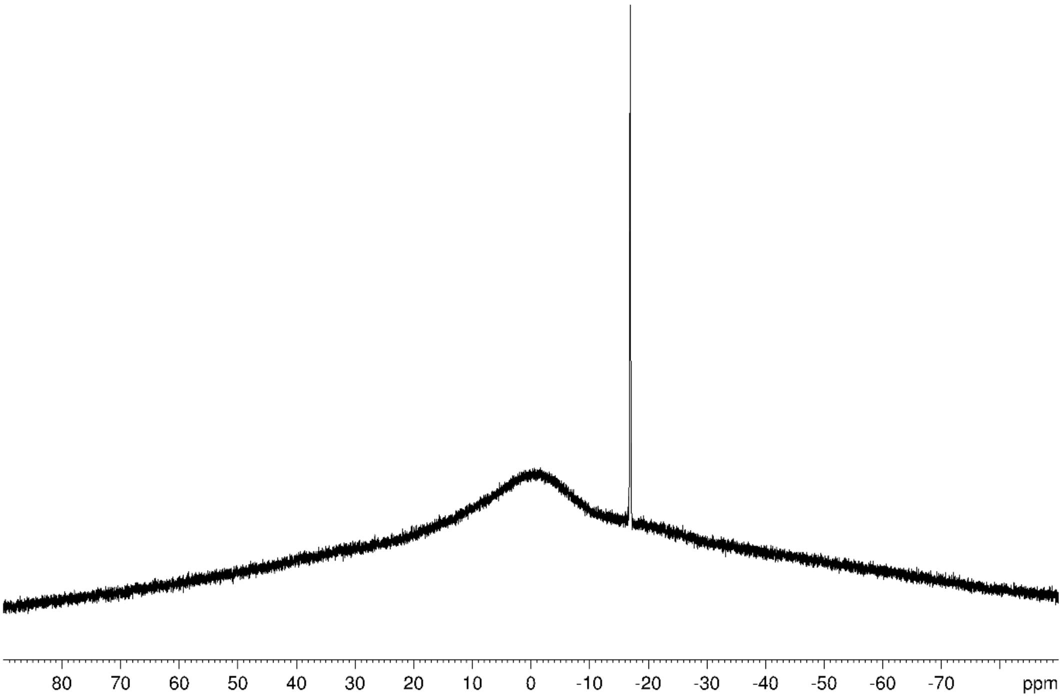

Figure S54. ^{11}B NMR spectrum of the reaction mixture of [IDipp·GeH_2BH_2][BAr^F] with $NHEt_2$ in CH_2Cl_2/C_6D_6 at 298 K.

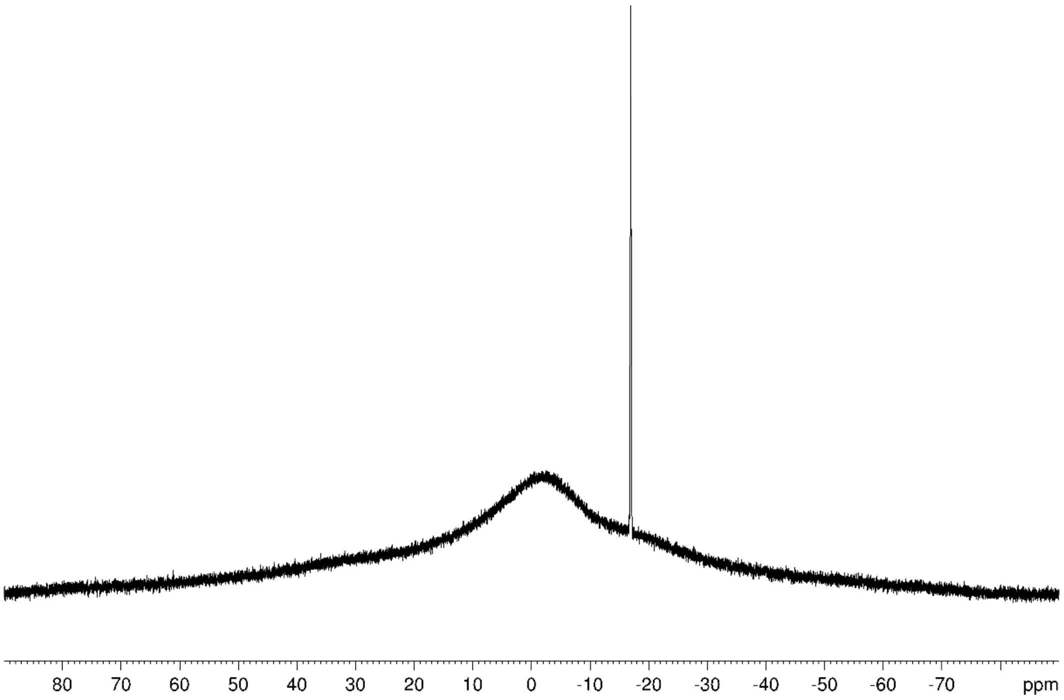

Figure S55. $^{11}B\{^1H\}$ NMR spectrum of the reaction mixture of [IDipp·GeH_2BH_2][BAr^F] with $NHEt_2$ in CH_2Cl_2/C_6D_6 at 298 K.

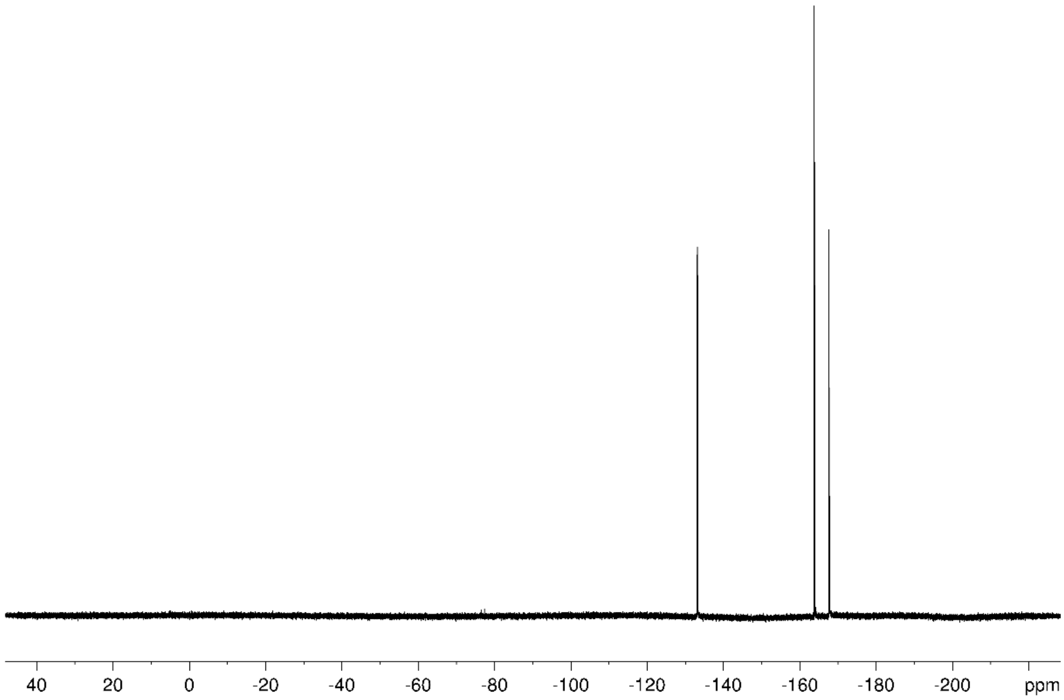

Figure S56. ^{19}F NMR spectrum of the reaction mixture of [IDipp·GeH_2BH_2][BAr^F] with $NHEt_2$ in CH_2Cl_2/C_6D_6 at 298 K.

v. Reaction of [IDipp·GeH_2BH_2][OTf] with $KBAr^F$ in o-DFB

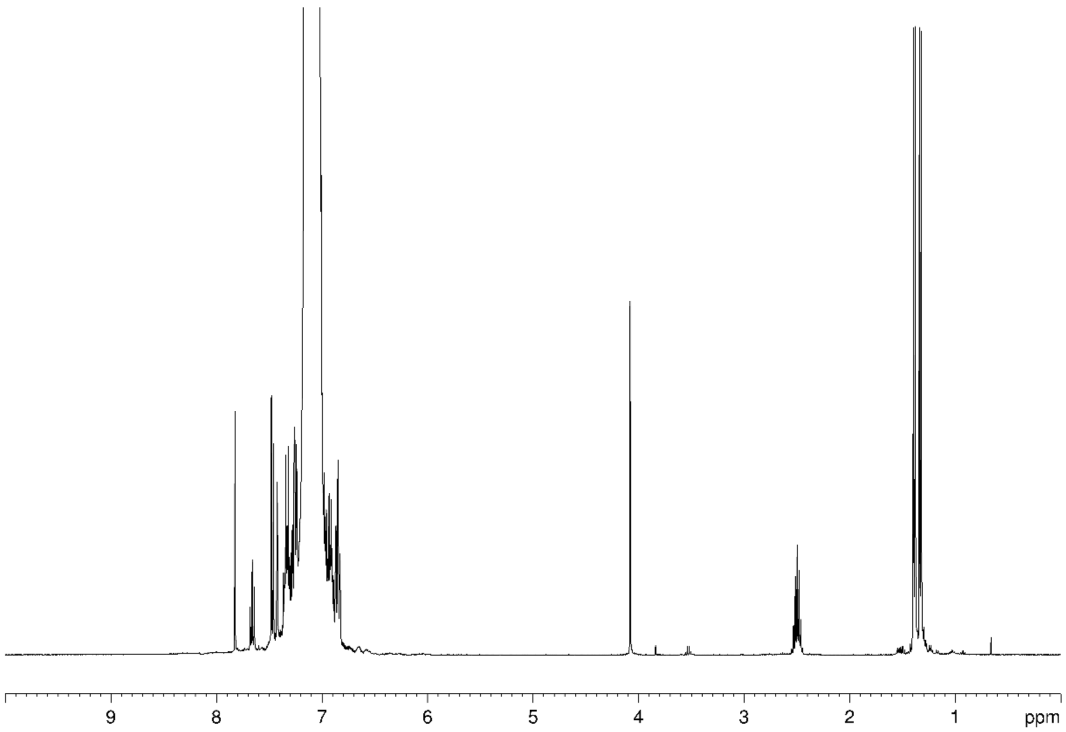

Figure S57. ^{1}H NMR spectrum of [IDipp·GeH_3][BAr^F] in o-DFB/C_6D_6 at 298 K.

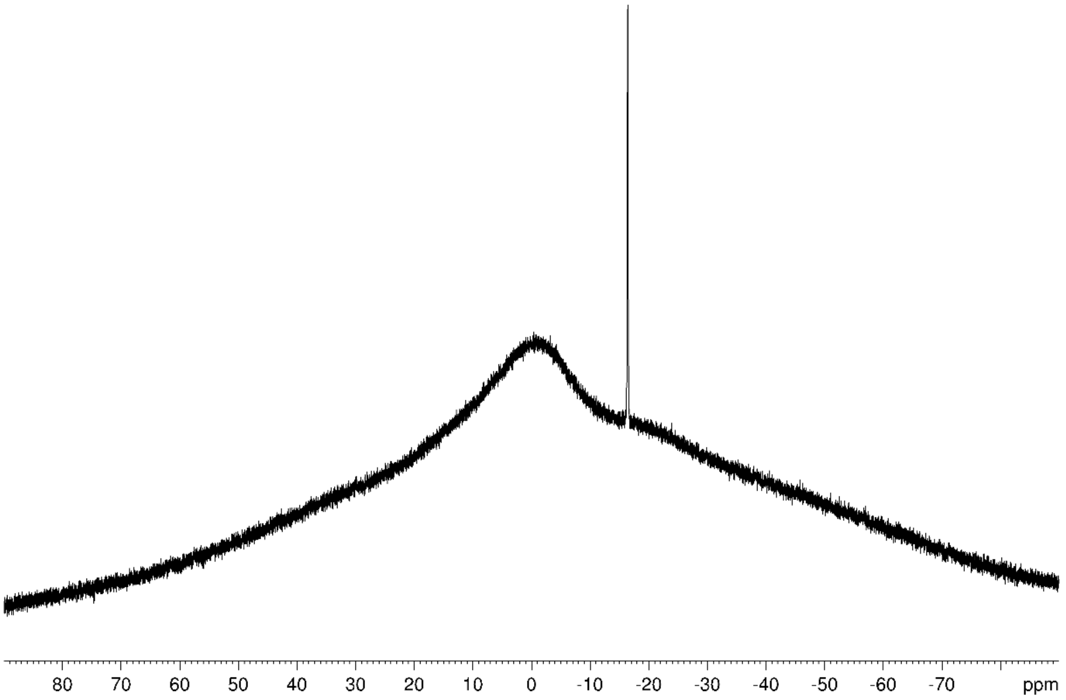

Figure S58. ^{11}B NMR spectrum of [IDipp·GeH_3][BAr^F] in o-DFB/C_6D_6 at 298 K.

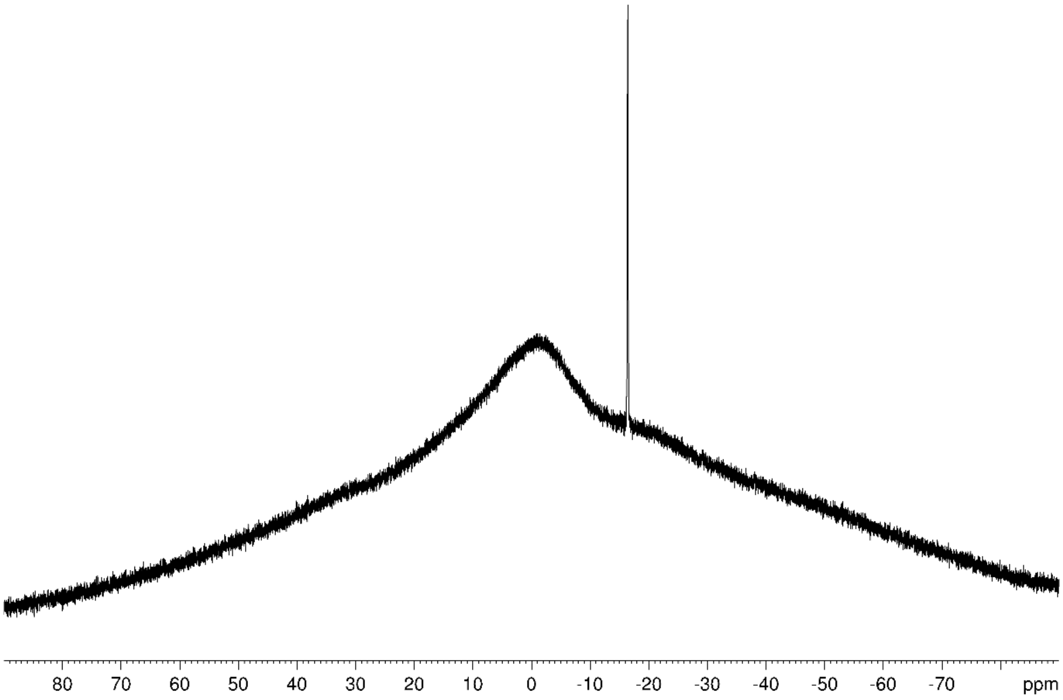

Figure S59. $^{11}B\{^{1}H\}$ NMR spectrum of [IDipp·GeH_3][BAr^F] in o-DFB/C_6D_6 at 298 K.

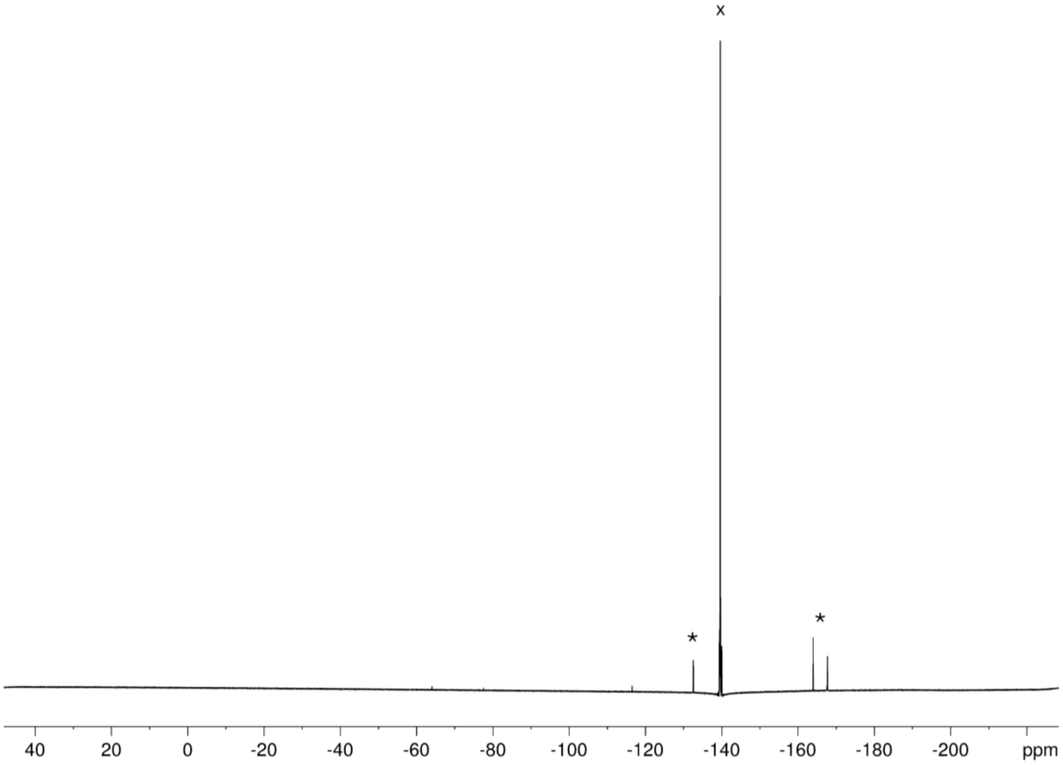

Figure S60. ^{19}F NMR spectrum of the reaction mixture of [IDipp·GeH_3][BAr^F] in o-DFB/C_6D_6 at 298 K. * - $[BAr^F]^-$, x – o-DFB.

vi. Reaction of [IDipp·GeH$_2$BH$_2$][OTf] with TlTEF in o-DFB

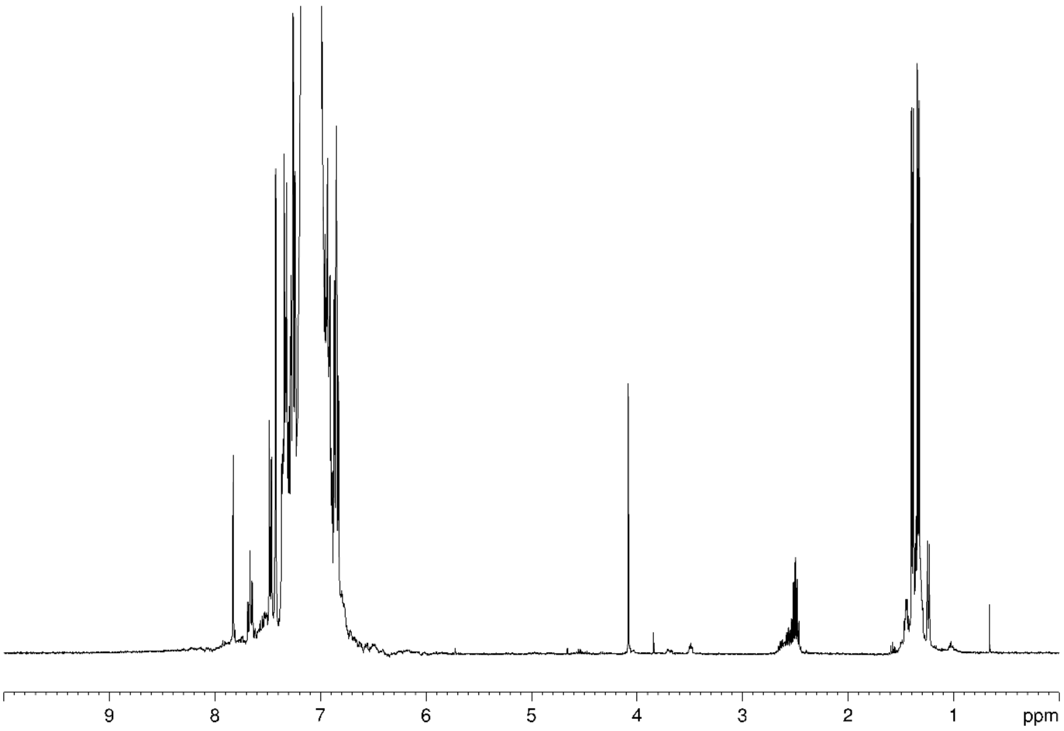

Figure S61. ^{1}H NMR spectrum of [IDipp · GeH$_3$][TEF] in o-DFB/C_6D_6 at 298 K.

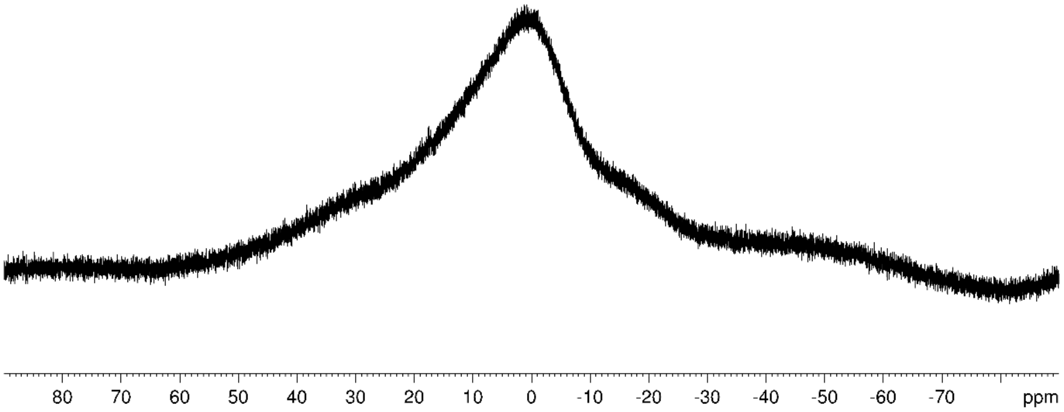

Figure S62. ^{11}B NMR spectrum of [IDipp · GeH$_3$][TEF] in o-DFB/C_6D_6 at 298 K.

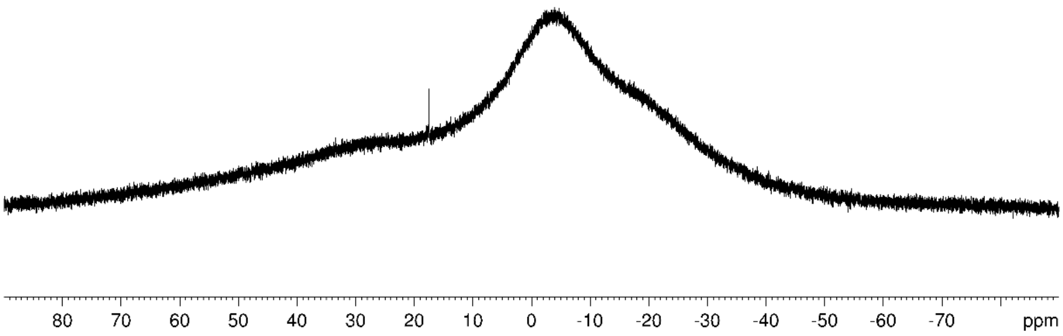

Figure S63. $^{11}B\{H\}$ NMR spectrum of [IDipp · GeH_3][TEF] in o-DFB/C_6D_6 at 298 K.

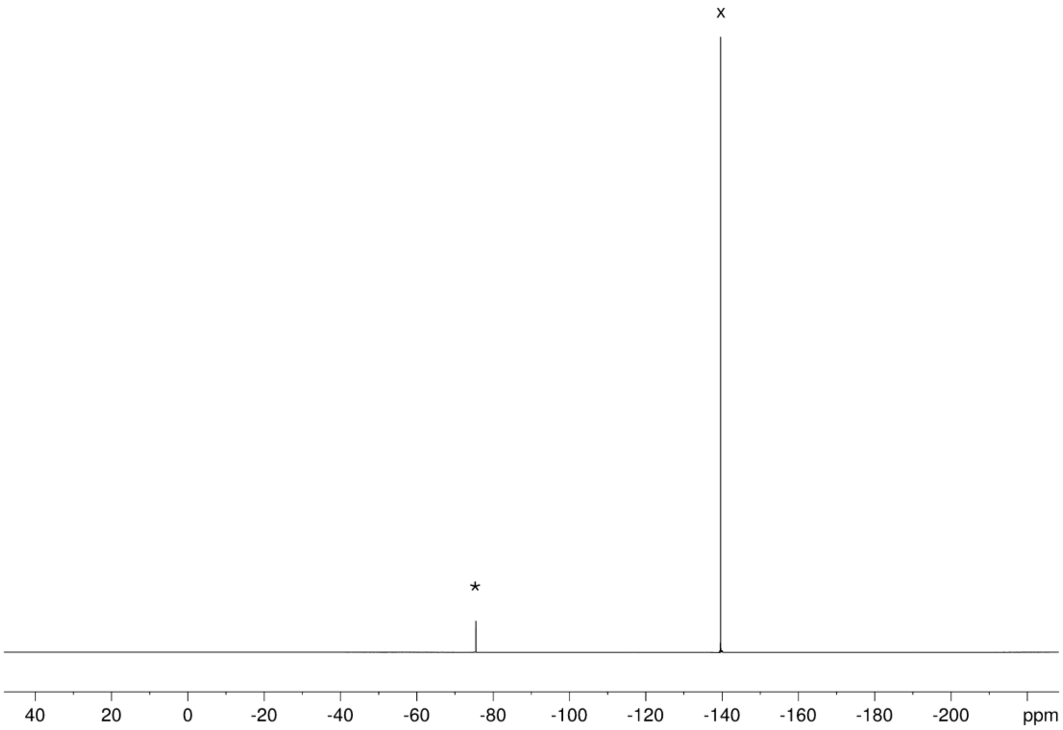

Figure S64. ^{19}F NMR spectrum of [IDipp · GeH_3][TEF] in o-DFB/C_6D_6 at 298 K. * - $[TEF]^-$, x – o-DFB.

k. IDipp·GeH_2BH_2OTf (1) with TMEDA

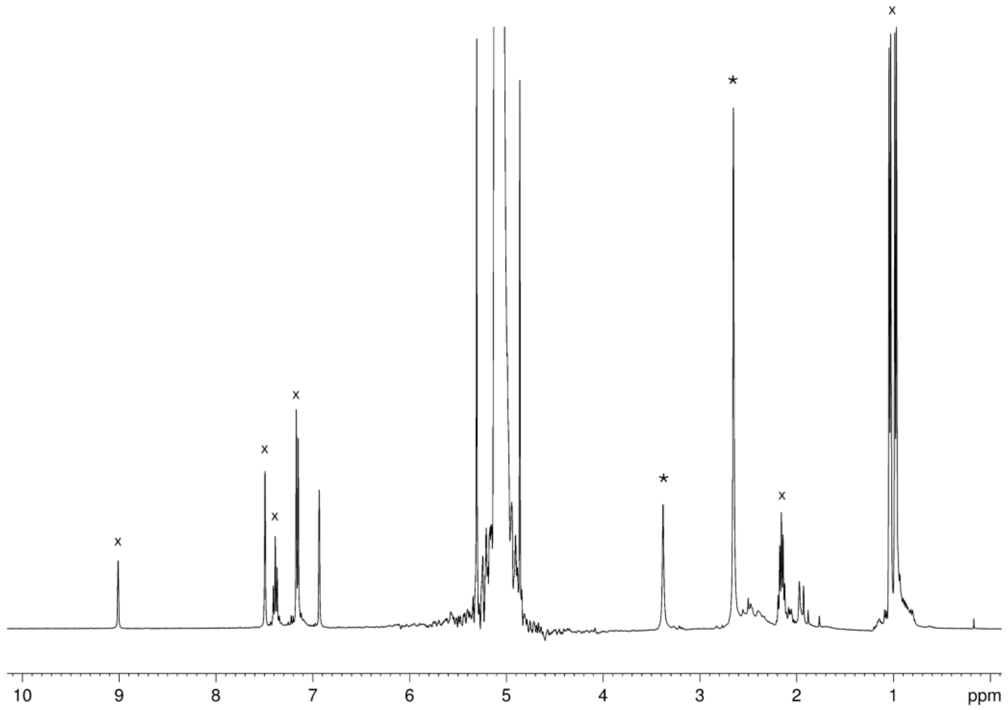

Figure S65. ^{1}H NMR spectrum of mixture of [IDippH][OTf] and [BH_2·TMEDA][OTf] in CH_2Cl_2/C_6D_6 at 298 K. * – [BH_2·TMEDA][OTf], x – [IDippH][OTf].

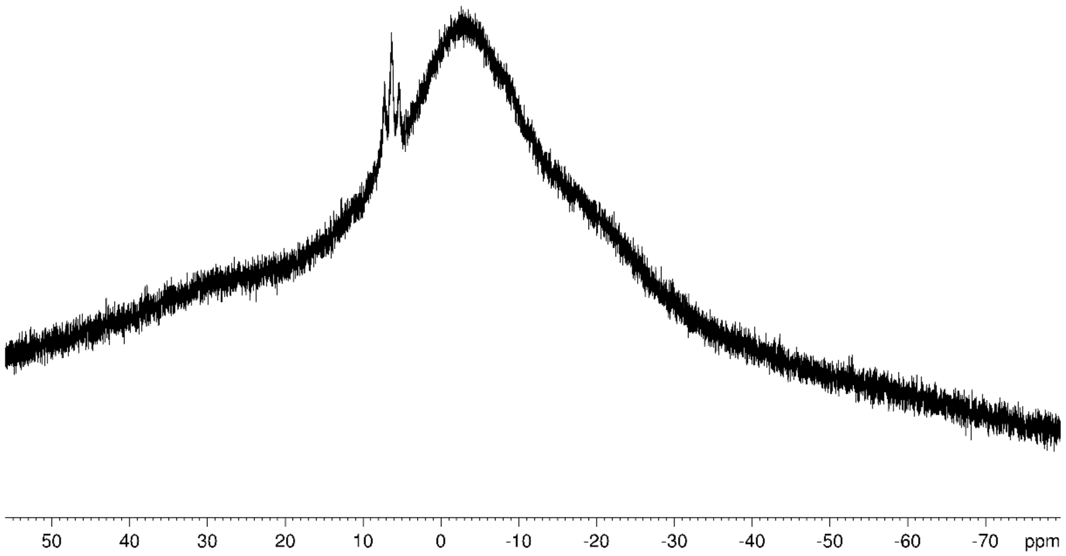

Figure S66. ^{11}B NMR spectrum of [BH_2·TMEDA][OTf] in CH_2Cl_2/C_6D_6 at 298 K.

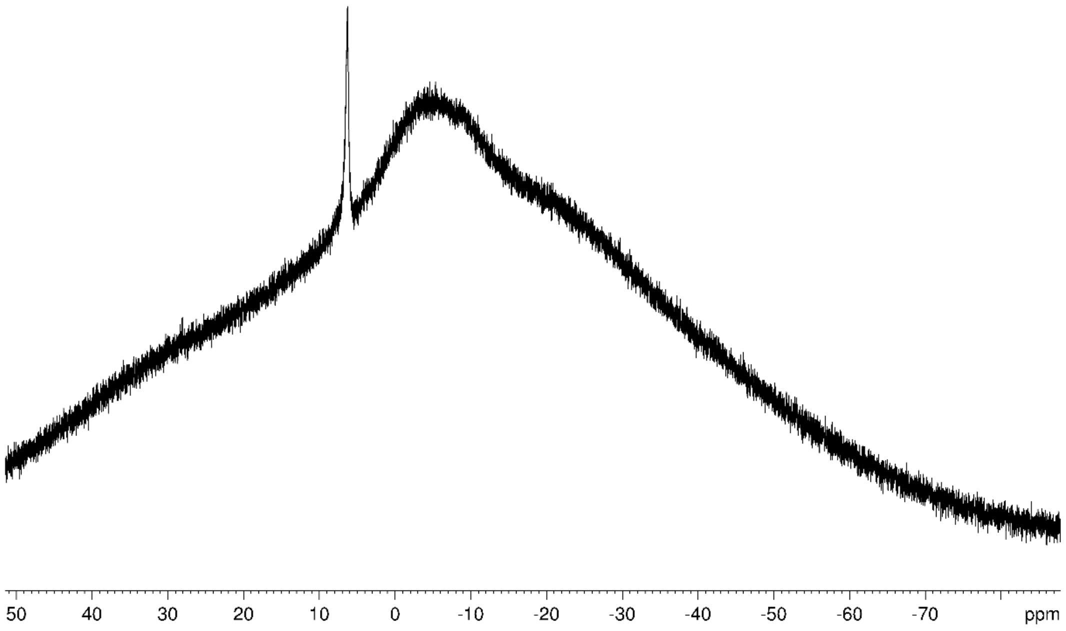

Figure S67. $^{11}B\{^{1}H\}$ NMR spectrum of $[BH_2{\cdot}TMEDA][OTf]$ in CH_2Cl_2/C_6D_6 at 298 K.

3. Crystallographic data

Single-crystal X-Ray diffraction experiments were performed on a XtaLAB Synergy R DW system (Rigaku) equipped with a HyPix-Arc 150 detector. Data were collected using Cu-Kα radiation (λ = 1.54178 Å). Data reduction, scaling and absorption corrections were performed using CrysAlisPro[1] (Rigaku). Using Olex2[2] all structures were solved with ShelXT[3] and a least-square refinement on $F^2$was carried out with ShelXL[4]. All non-hydrogen atoms were refined anisotropically. All the hydrogen atoms at the carbon atoms been located in idealized positions and refined isotropically according to the riding model. Figures were created with Olex2.

Crystallographic data and details of the experiments are given in Tables S1, S2 and S3. CIF files with comprehensive information on the details of the diffraction experiments and full tables of bond lengths and angles are deposited in Cambridge Crystallographic Data Centre under the deposition codes CCDC 2525632 (**2**), 2525633 (**3a**), 2525634 (**3b**), 2525635 (**3c**), 2525636 (**5**), 2525637 (**7**), 2525638 (**8**) and 2525639 (**9**). These data can be obtained free of charge at www.ccdc.cam.ac.uk/conts/retrieving.html (or from the Cambridge Crystallographic Data Centre, 12 Union Road, Cambridge CB2 1EZ, UK; Fax: + 44-1223-336-033; e-mail: deposit@ccdc.cam.ac.uk).

Table S1: Crystallographic data for compounds **2**, **3a**, **3b**.

Compound	**2**	**3a**	**3b**
Internal naming	TP43	TP53	TP60
CCDC number	2525632	2525633	2525634
Formula	$C_{34}H_{55}BF_3GeN_3O_3S$	$C_{33}H_{51}BCl_2F_3N_3O_3S$	$C_{31}H_{47}BF_3N_3O_3S$
d_{calc}/g/cm^3	1.274	1.256	1.153
μ/mm^{-1}	2.030	2.500	1.225
Formula Weight	726.27	708.582	609.58
Color	colorless	colorless	colorless
Shape	block	block	Block
Size/mm^3	0.16 × 0.11 × 0.09	0.18 × 0.17 × 0.1	0.34 × 0.21 × 0.19
T/K	123.01(10)	123.00(10)	123.00(10)
Crystal System	monoclinic	orthorhombic	Triclinic
Space Group	$P2_1/n$	*Pbcn*	$P\bar{1}$
a/Å	9.32490(10)	19.1793(3)	9.4668(3)
b/Å	23.7393(2)	23.1886(3)	10.8636(4)
c/Å	17.11400(10)	16.8526(2)	17.7032(5)
α/°	90	90	85.197(3)
β/°	91.7890(10)	90	77.302(3)
γ/°	90	90	81.843(3)
V/Å^3	3786.62(6)	7495.04(18)	1755.56(10)
Z	4	8	2
Z'	1	1	1
Wavelength/Å	1.54184	1.54184	1.54184
Radiation type	Cu K_α	Cu K_α	Cu K_α
θ_{min}/°	6.368	5.98	5.124
θ_{max}/°	150.632	150.72	150.99
Measured Refl's.	32553	37341	24018
Indep't Refl's	7692	7578	7078
R_{int}	0.0288	0.0496	0.0375
Parameters	442	443	405
Restraints	0	0	0
Largest Peak	0.31	0.61	0.32
Deepest Hole	-0.31	-0.59	-0.35
GooF	1.063	1.022	1.078
wR_2 (all data)	0.0731	0.1628	0.1425
wR_2	0.0711	0.1592	0.1371
R_1 (all data)	0.0340	0.0887	0.0572
R_1	0.0289	0.0803	0.0491

Table S2: Crystallographic data for compounds **3c**, **5**, **7**.

Compound	**3c**	**5**	**7**
Internal naming	TP49	TP218	TP90
CCDC number	2525635	2525636	2525637
Formula	$C_{32}H_{49}BF_3N_3O_3S$	$C_{34}H_{47}BCl_2F_3GeN_3O_3S$	$C_{67.5}H_{91.5}B_2F_6Ge_2N_6O_6S_2$
d_{calc}/g/cm^3	1.196	1.349	1.269
μ/mm^{-1}	1.253	3.262	2.055
Formula Weight	623.61	789.10	1427.88
Color	colorless	colorless	colorless
Shape	block	plate	prism
Size/mm^3	0.29 × 0.1 × 0.09	0.23 × 0.16 × 0.07	0.34 × 0.21 × 0.11
T/K	122.97(10)	123.00(10)	123.00(10)
Crystal System	triclinic	triclinic	triclinic
Space Group	$P\bar{1}$	$P\bar{1}$	$P\bar{1}$
a/Å	9.2322(4)	9.16790(10)	9.58230(10)
b/Å	10.9809(4)	12.9491(2)	18.0266(3)
c/Å	17.4941(7)	17.4730(4)	23.4080(3)
α/°	87.023(3)	82.613(2)	107.0970(10)
β/°	78.994(4)	79.926(2)	93.3170(10)
γ/°	84.311(3)	72.6890(10)	102.7730(10)
V/Å^3	1731.33(12)	1943.36(6)	3736.21(9)
Z	2	2	2
Z'	1	1	1
Wavelength/Å	1.54184	1.54184	1.54184
Radiation type	Cu K_α	Cu K_α	Cu K_α
θ_{min}/°	5.15	7.147	2.648
θ_{max}/°	150.38	150.304	75.508
Measured Refl's.	27902	44584	66278
Indep't Refl's	6979	7859	15168
R_{int}	0.0424	0.0235	0.0215
Parameters	415	476	1102
Restraints	0	3	785
Largest Peak	0.30	0.48	1.036
Deepest Hole	-0.38	-0.45	-0.68
GooF	1.101	1.012	1.027
wR_2 (all data)	0.1267	0.0922	0.1581
wR_2	0.1235	0.0912	0.1525
R_1 (all data)	0.0490	0.0357	0.0638
R_1	0.0445	0.0343	0.0564

Table S3: Crystallographic data for compounds **8**, **9**.

Compound	**8**	**9**
Internal naming	TP214	TP256
CCDC number	2525638	2525639
Formula	$C_{84}H_{108}B_2Cl_4F_6Ge_2N_4O_6P_2S_2$	$C_{86}H_{104}B_2Cl_4F_6Ge_2Mo_4N_4O_{14}P_4S_2$
d_{calc}/g/cm^3	1.238	1.549
μ/mm^{-1}	2.963	6.999
Formula Weight	1818.40	2412.09
Color	colorless	red
Shape	block	plate
Size/mm^3	0.93 × 0.39 × 0.18	0.4 × 0.19 × 0.14
T/K	123.00(10)	123.00(10)
Crystal System	triclinic	triclinic
Space Group	$P\bar{1}$	$P\bar{1}$
a/Å	12.2318(2)	17.8064(2)
b/Å	12.8241(2)	18.2605(2)
c/Å	16.4675(2)	19.0085(2)
α/°	87.9730(10)	100.3330(10)
β/°	70.8630(10)	98.1740(10)
γ/°	88.3800(10)	118.1830(10)
V/Å^3	2438.47(6)	5172.85(11)
Z	1	2
Z'	0.5	1
Wavelength/Å	1.54184	1.54184
Radiation type	Cu K_α	Cu K_α
θ_{min}/°	2.841	2.857
θ_{max}/°	75.351	75.078
Measured Refl's.	122448	120804
Indep't Refl's	9905	20949
R_{int}	0.0492	0.0237
Parameters	865	1174
Restraints	877	0
Largest Peak	0.70	1.00
Deepest Hole	-0.55	-0.76
GooF	1.055	1.099
wR_2 (all data)	0.2500	0.0754
wR_2	0.2467	0.0751
R_1 (all data)	0.0817	0.0296
R_1	0.0788	0.0291

4. Crystal Structures

a. [IDipp · GeH_2BH_2 · NEt_3][OTf] (2)

2 was crystallized from a concentrated DCM solution layered with threefold excess of *n*-hexane at +9 °C as colorless blocks in the monoclinic space group $P2_1/n$. The structure of **2** in the solid state is shown in Figure S68.

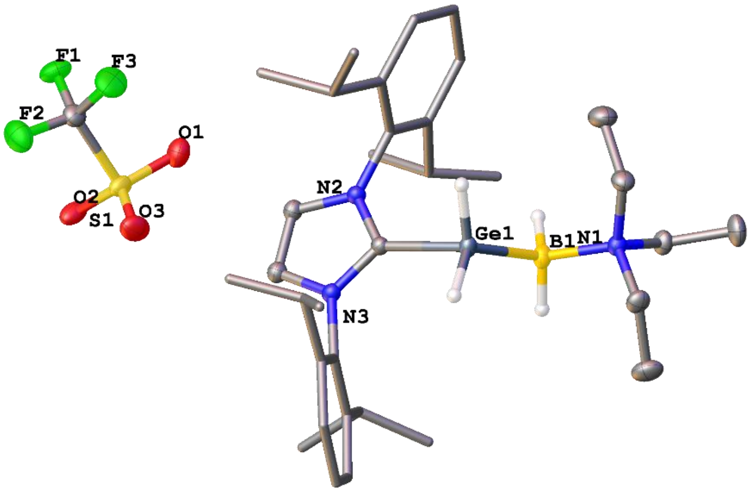

Figure S68. Molecular structure of **2** in the solid state, with anisotropic displacement ellipsoids at a 50% probability level. Hydrogen atoms bound to carbon are omitted for clarity. Selected bond lengths [Å] and angles [°]: Ge1-C1 1.9922(15), Ge1-B1 2.0734(18), B1-N1 1.609(2), C1-Ge1-B1 106.82(7), Ge1-B1-N1 113.41(11).

The hydrogen atom positions located on the Ge and B atoms were located from the difference Fourier map and refined freely.

b. [IDipp · BH_2 · $NHEt_2$][OTf] (3a)

3a was crystallized from a concentrated DCM solution layered with threefold excess of *n*-hexane at +9 °C as colorless blocks in the orthorhombic space group *Pbcn*. The structure of **3a** in the solid state is shown in Figure S69.

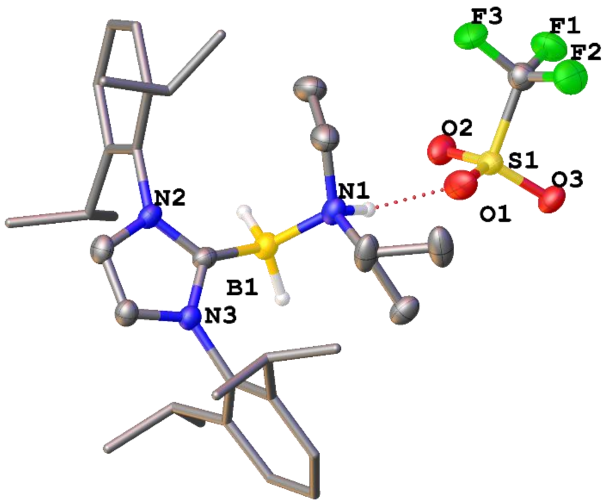

Figure S69. Molecular structure of **3a** in the solid state, with anisotropic displacement ellipsoids at a 50% probability level. Hydrogen atoms bound to carbon are omitted for clarity. Selected bond lengths [Å] and angles [°]: C1-B1 1.629(5), B1-N1 1.603(5), H1N-O1 1.986(6), C1-B1-N1 114.0(3), N1-H1N1-O1 137.36(18).

The hydrogen atom positions located on the N and B atoms were located from the difference Fourier map and refined freely. One of the ethyl groups of $NHEt_2$ appears to be disordered over two positions.

c. [IDipp·BH_2·$NH_2{}^{i}Pr$][OTf] (3b)

3b was crystallized from a concentrated DCM solution layered with threefold excess of *n*-hexane at +9 °C as colorless blocks in the triclinic space group $P\bar{1}$. The structure of **3b** in the solid state is shown in Figure S70.

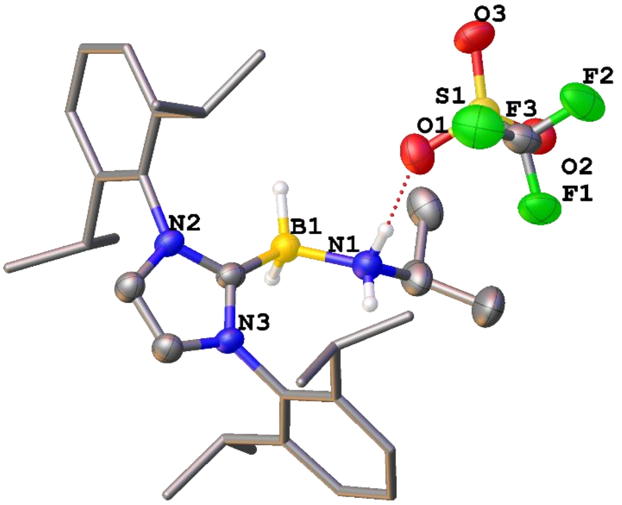

Figure S70. Molecular structure of **3b** in the solid state, with anisotropic displacement ellipsoids at a 50% probability level. Hydrogen atoms bound to carbon are

omitted for clarity. Selected bond lengths [Å] and angles [°]: C1-B1 1.603(2), B1-N1 1.604(2), H1N-O1 1.944(4), C1-B1-N1 111.63(14), N1-H1N1-O1 131.19(19).

The hydrogen atom positions located on the N and B atoms were located from the difference Fourier map and refined freely.

d. [IDipp·BH$_2$·NH$_2$^{t}Bu][OTf] (3c)

3c was crystallized from a concentrated DCM solution layered with threefold excess of *n*-hexane at +9 °C as colorless blocks in the triclinic space group $P\bar{1}$. The structure of **3c** in the solid state is shown in Figure S71.

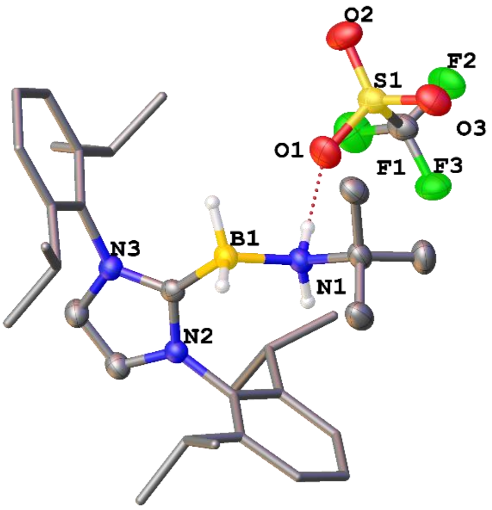

Figure S71. Molecular structure of **3c** in the solid state, with anisotropic displacement ellipsoids at a 50% probability level. Hydrogen atoms bound to carbon are omitted for clarity. Selected bond lengths [Å] and angles [°]: C1-B1 1.612(2), B1-N1 1.608(2), H1N-O1 2.010(2), C1-B1-N1 111.26(12), N1-H1N1-O1 144.16(15).

The hydrogen atom positions located on the N and B atoms were located from the difference Fourier map and refined freely.

e. [IDipp·GeH$_2$BH$_2$·Py][OTf] (5)

5 was crystallized from a concentrated DCM solution layered with threefold excess of *n*-hexane at +9 °C as colorless plates in the triclinic space group $P\bar{1}$. The structure of **5** in the solid state is shown in Figure S72.

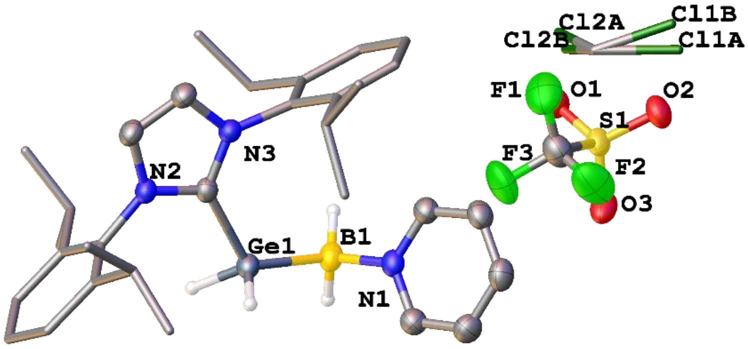

Figure S72. Molecular structure of **5** in the solid state, with anisotropic displacement ellipsoids at a 50% probability level. Hydrogen atoms bound to carbon are omitted for clarity. Selected bond lengths [Å] and angles [°]: Ge1-C1 2.0101(16), Ge1-B1 2.080(2), B1-N1 1.574(2), C1-Ge1-B1 122.05(8), Ge1-B1-N1 110.56(12).

The hydrogen atom positions located on the Ge and B atoms were located from the difference Fourier map and refined freely. Compound **5** incorporates a molecule of CH_2Cl_2 in its crystal structure which appears to be disordered over two positions. SADI restraints were applied to model this disorder.

f. [IDipp·GeH_2BH_2·bipy·BH_2GeH_2·IDipp][OTf]$_2$ (7)

7 was crystallized from a concentrated DCM solution layered with threefold excess of *n*-hexane at +9 °C as colorless prisms in the triclinic space group $P\bar{1}$. The structure of **7** in the solid state is shown in Figure S73.

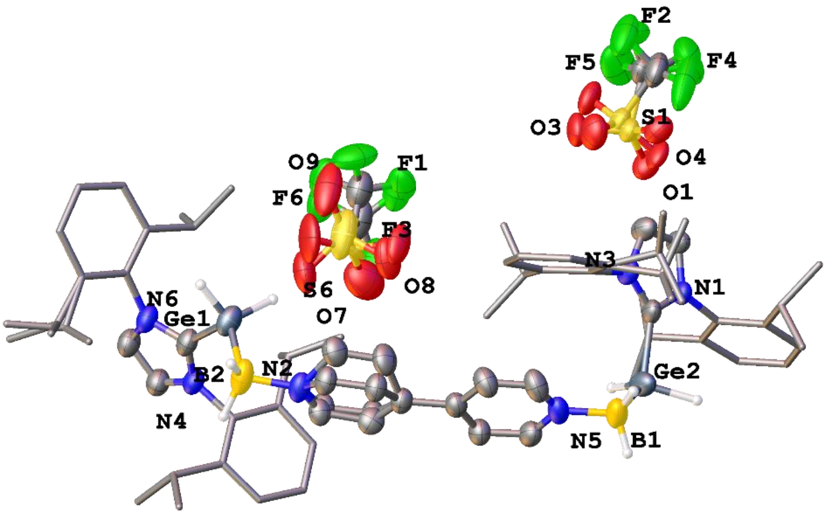

Figure S73. Molecular structure of **7** in the solid state, with anisotropic displacement ellipsoids at a 50% probability level. Hydrogen atoms bound to carbon are omitted for clarity. Selected bond lengths [Å] and angles [°]: Ge1-C10 2.012(3), Ge1-B2

2.079(4), B2-N2 1.585(4), Ge2-C8 1.997(3), Ge2-B1 2.054(4), B1-N5 1.585(4), C10-Ge1-B2 113.46(17), C8-Ge2-B1 123.01(17), Ge1-B2-N2 107.3(2) , Ge2-B1-N5 111.3(2).

The hydrogen atom positions located on the Ge and B atoms were located from the difference Fourier map and refined freely. Compound **7** has the disordered at the ^{i}Pr substituents and at the bipy backbone. The triflate anions are also disordered over two positions. The restraints SADI, DFIX, DANG, SIMU and RIGU were applied to model this disorder. Upon refinement of the crystal structure of compound **7**, there appears to be significant electron density within solvent accessible voids. It was attributed to heavily disordered hexane solvent molecules (0.25 per asymmetric unit), this issue was treated by refinement with a solvent mask (included in the Olex2 software package).

g. [IDipp·GeH_2BH_2·dppe·BH_2GeH_2·IDipp][OTf]$_2$ (8)

8 was crystallized from a concentrated DCM solution -30 °C as colorless plates in the triclinic space group $P\bar{1}$. The structure of **8** in the solid state is shown in Figure S74.

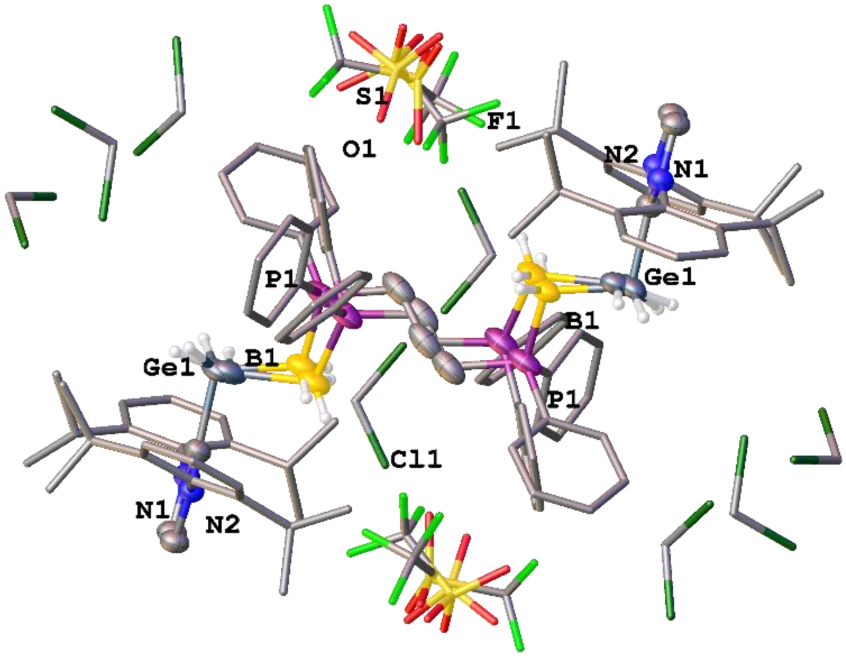

Figure S74. Molecular structure of **8** in the solid state, with anisotropic displacement ellipsoids at a 50% probability level. Hydrogen atoms bound to carbon are omitted for clarity. Selected bond lengths [Å] and angles [°]: Ge1-C6 1.975(5), Ge1-B1 2.037(19), B1-P1 1.891(18), C6-Ge1-B1 106.1(5), Ge1-B1-P1 110.5(6).

The hydrogen atom positions located on the Ge and B atoms were located from the difference Fourier map and refined freely. Compound **8** incorporates in its crystal structure four molecules of CH_2Cl_2 per asymmetric unit. Notably, both the anion, as well as the di-cation are heavily disordered. Additionally, the disorder of the di-cationic part

of **8** is complicated by its position on the inversion centre in the unit cell. Overall, modelling the disorder requires multiple DFIX, DANG, SIMU and ISOR restraints, but ultimately cannot be fully resolved affording poor R_1 = 7.88% and wR_2 = 25% values. Thus, structural parameters derived of this data set should be considered carefully.

h. $[Cp_2Mo_2(CO)_4(\eta^2\text{-}P_2)\cdot BH_2GeH_2\cdot IDipp][OTf]$ (9)

9 was crystallized from a concentrated DCM solution layered with threefold excess of *n*-hexane at +9 °C as red plates in the triclinic space group $P\bar{1}$. The structure of **9** in the solid state is shown in Figure S75.

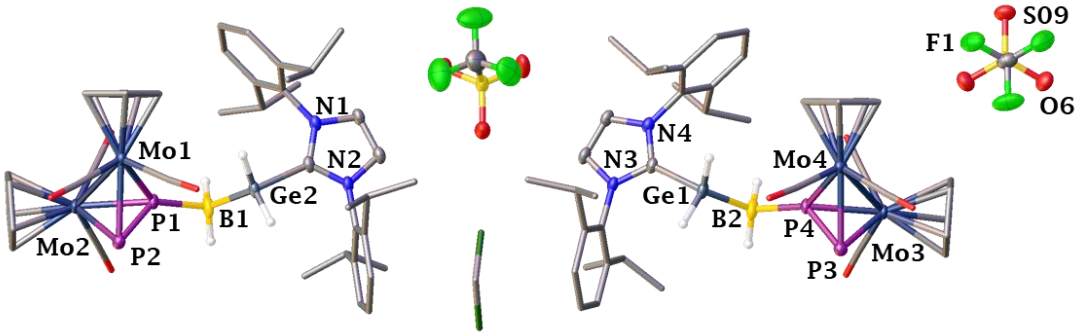

Figure S75. Molecular structure of **9** in the solid state, with anisotropic displacement ellipsoids at a 50% probability level. Hydrogen atoms bound to carbon are omitted for clarity. Selected bond lengths [Å] and angles [°]: Ge1-C28 1.994(2), Ge1-B2 2.055(3), B2-P4 1.949(3), P4-P3 2.0725(8), Ge1-C6 1.991(2), Ge2-B1 2.053(3), B1-P1 1.953(3), P1-P2 2.0742(8), C28-Ge1-B2 113.07(10), Ge1-B2-P4 106.55(13), B2-P4-P3 135.45(11), C6-Ge2-B1 115.82(10), Ge2-B1-P1 103.40(13), B1-P1-P2 134.55(11).

The hydrogen atom positions located on the Ge and B atoms were located from the difference Fourier map and refined freely. Compound **9** incorporates in its crystal structure two molecules of CH_2Cl_2 per an asymmetric unit, one of which appears to be significantly disordered. This issue was treated by refinement with a solvent mask (included in the Olex2 software package).

5. Computational Details

Table S4. Total energies E_0°, sum of electronic and thermal enthalpies H_{298}° (Hartree) and standard entropies S_{298}° (cal mol^{-1} K^{-1}). B3LYP/def2-TZVP level of theory.

Compound	E_0°	H_{298}°	S_{298}°
GeH_2	-2078.19678383	-2078.182210	54.092
NEt_3	-292.52733603	-292.312124	93.427
$NHEt_2$	-213.88388629	-213.727260	80.108
BH_2NEt_2	-239.37543166	-239.204777	84.024
$IDippH^+$	-1160.89771178	-1160.284479	205.009
IDipp·GeH_3^+ (**4**$^+$)	-3239.14422141	-3238.514834	214.034
IDipp·$GeH_2BH_2^+$ (**1**$^+$)	-3264.57731291	-3263.933621	222.649
IDipp·GeH_2BH_2·NEt_3^+ (**2**$^+$)	-3557.17150482	-3556.305958	263.266
IDipp·GeH_2BH_2·$NHEt_2^+$	-3478.53034503	-3477.723901	254.821
IDipp·BH_2·NEt_3^+	-1478.91186135	-1478.064314	240.230
IDipp·BH_2·$NHEt_2^+$ (**3a**$^+$)	-1400.28091558	-1399.492347	234.845

Table S5. Optimized geometries of theoretically studied compounds. xyz coordinates in angstroms. B3LYP/def2-TZVP level of theory.

GeH_2			
32	0.000000000	0.066076000	0.000000000
1	1.137855000	-1.057626000	0.000000000
1	-1.137855000	-1.056814000	0.000000000
NEt_3			
7	-0.000119000	-0.297122000	-0.018110000
6	1.213823000	-1.079667000	0.198343000
6	-1.214177000	-1.079476000	0.198223000
1	-1.389126000	-1.268424000	1.273981000
1	-1.060622000	-2.056521000	-0.265300000
1	1.060067000	-2.056808000	-0.264922000
1	1.388738000	-1.268376000	1.274144000
6	2.460372000	-0.445945000	-0.409387000
1	3.323357000	-1.098050000	-0.257867000
1	2.327340000	-0.289877000	-1.481249000
1	2.695227000	0.517112000	0.047050000
6	-2.460541000	-0.445331000	-0.409391000
1	-2.695362000	0.517571000	0.047408000
1	-2.327317000	-0.288827000	-1.481170000
1	-3.323637000	-1.097385000	-0.258262000
6	0.000015000	0.990172000	0.692590000
1	-0.871992000	1.043130000	1.353805000
1	0.871646000	1.042604000	1.354342000

6	0.000576000	2.202332000	-0.236364000
1	-0.879893000	2.200627000	-0.880355000
1	0.880969000	2.199784000	-0.880454000
1	0.001029000	3.130786000	0.341527000

$NHEt_2$

7	-0.000012000	-0.268773000	0.073430000
6	-1.223131000	0.517237000	-0.022074000
6	1.223196000	0.517373000	-0.021353000
1	1.236812000	1.215319000	0.821728000
1	1.252184000	1.136927000	-0.935138000
1	-1.252248000	1.135888000	-0.936493000
1	-1.236719000	1.216093000	0.820254000
1	0.000346000	-0.983232000	-0.647701000
6	-2.457676000	-0.371178000	0.031294000
1	-3.369066000	0.227801000	-0.013856000
1	-2.477888000	-1.067185000	-0.811875000
1	-2.471561000	-0.956843000	0.951752000
6	2.457608000	-0.371223000	0.030990000
1	2.471320000	-0.958207000	0.950606000
1	2.477841000	-1.066074000	-0.813167000
1	3.369081000	0.227675000	-0.013258000

BH_2NEt_2

7	-0.000071000	0.136894000	-0.000156000
6	1.202414000	-0.709445000	0.000206000
6	-1.202482000	-0.709581000	-0.000386000
1	-1.149279000	-1.369417000	0.874321000
1	-1.149599000	-1.368443000	-0.875837000
1	1.149449000	-1.369082000	-0.874663000
1	1.149400000	-1.368527000	0.875499000
6	2.540827000	0.007649000	0.000034000
1	3.335660000	-0.741111000	0.000241000
1	2.666675000	0.635802000	-0.881750000
1	2.666524000	0.636140000	0.881577000
6	-2.540751000	0.007680000	0.000300000
1	-2.666062000	0.635559000	0.882335000
1	-2.666534000	0.636492000	-0.881003000
1	-3.335819000	-0.740830000	0.000111000
5	-0.000034000	1.526045000	-0.000089000
1	1.028099000	2.123305000	-0.000041000
1	-1.027883000	2.123816000	-0.000177000

$IDippH^+$

1	-0.000016000	0.000078000	-1.323053000
7	1.086986000	0.000000000	0.525857000
7	-1.086986000	0.000007000	0.525851000
6	0.000000000	0.000037000	-0.247387000
6	0.679027000	-0.000072000	1.844396000
1	1.385989000	-0.000106000	2.654164000

6	2.462700000	-0.000009000	0.052030000
6	-2.462709000	0.000016000	0.052036000
6	-3.089559000	1.237602000	-0.159001000
6	-0.679032000	-0.000050000	1.844397000
1	-1.386004000	-0.000064000	2.654156000
6	3.089585000	-1.237593000	-0.158923000
6	-4.407216000	1.202638000	-0.613212000
1	-4.931635000	2.131888000	-0.791292000
6	-2.409669000	-2.577764000	0.082359000
1	-1.396018000	-2.392186000	0.442895000
6	-3.089613000	-1.237560000	-0.158914000
6	-4.407272000	-1.202571000	-0.613112000
1	-4.931737000	-2.131810000	-0.791116000
6	3.089569000	1.237564000	-0.159011000
6	-5.058092000	0.000040000	-0.838867000
1	-6.081452000	0.000049000	-1.191226000
6	-2.409588000	2.577792000	0.082276000
1	-1.395873000	2.392190000	0.442620000
6	4.407239000	-1.202626000	-0.613139000
1	4.931685000	-2.131875000	-0.791148000
6	4.407221000	1.202581000	-0.613235000
1	4.931652000	2.131826000	-0.791312000
6	2.409634000	-2.577792000	0.082354000
1	1.396033000	-2.392211000	0.443029000
6	2.409647000	2.577771000	0.082304000
1	1.395919000	2.392202000	0.442628000
6	-2.286420000	-3.388785000	-1.217384000
1	-3.266749000	-3.648620000	-1.619308000
1	-1.748761000	-4.319943000	-1.031540000
1	-1.749443000	-2.834701000	-1.989602000
6	5.058078000	-0.000025000	-0.838900000
1	6.081434000	-0.000032000	-1.191271000
6	-3.130307000	-3.386168000	1.173206000
1	-3.193218000	-2.830298000	2.110312000
1	-2.596002000	-4.317682000	1.367014000
1	-4.146460000	-3.644618000	0.872068000
6	-3.130071000	3.386043000	1.173346000
1	-4.146282000	3.644490000	0.872396000
1	-2.595765000	4.317549000	1.367190000
1	-3.192819000	2.830050000	2.110392000
6	3.130383000	-3.386271000	1.173075000
1	2.596054000	-4.317760000	1.366935000
1	3.193453000	-2.830423000	2.110184000
1	4.146481000	-3.644769000	0.871791000
6	3.130164000	3.385927000	1.173428000
1	4.146383000	3.644352000	0.872488000
1	3.192898000	2.829856000	2.110429000

1	2.595894000	4.317437000	1.367347000
6	2.286658000	3.389017000	-1.217314000
1	1.749061000	4.320191000	-1.031378000
1	1.749726000	2.835110000	-1.989691000
1	3.267055000	3.648842000	-1.619079000
6	-2.286564000	3.388976000	-1.217380000
1	-1.749716000	2.834993000	-1.989760000
1	-1.748875000	4.320109000	-1.031503000
1	-3.266956000	3.648875000	-1.619109000
6	2.286195000	-3.388724000	-1.217425000
1	3.266464000	-3.648478000	-1.619548000
1	1.749055000	-2.834604000	-1.989504000
1	1.748613000	-4.319920000	-1.031555000
IDipp·GeH_3^+ (**4**$^+$)			
32	0.025012000	0.032095000	-2.063057000
7	1.075852000	-0.011080000	0.735698000
7	-1.087962000	-0.011674000	0.735842000
6	-0.006148000	-0.001914000	-0.069120000
6	0.672692000	-0.027344000	2.053035000
1	1.379680000	-0.037123000	2.862476000
6	2.462678000	-0.005407000	0.300021000
6	-2.479370000	-0.005606000	0.315296000
6	-3.115931000	1.233476000	0.143129000
6	-0.682742000	-0.027677000	2.053240000
1	-1.390028000	-0.037592000	2.862608000
6	3.107116000	-1.240592000	0.118892000
6	-4.455459000	1.204546000	-0.243435000
1	-4.986553000	2.135712000	-0.388219000
6	-2.444303000	-2.582924000	0.362449000
1	-1.390213000	-2.404229000	0.582899000
6	-3.124109000	-1.239587000	0.136284000
6	-4.463345000	-1.199770000	-0.249666000
1	-5.000447000	-2.126623000	-0.399932000
6	3.098852000	1.234482000	0.121660000
6	-5.121087000	0.005143000	-0.440031000
1	-6.161328000	0.009355000	-0.738930000
6	-2.428980000	2.571099000	0.381332000
1	-1.375312000	2.385069000	0.597733000
6	4.443162000	-1.200762000	-0.277488000
1	4.980177000	-2.127099000	-0.429042000
6	4.435120000	1.204160000	-0.275604000
1	4.966204000	2.134209000	-0.424865000
6	2.433404000	-2.581246000	0.377901000
1	1.360643000	-2.411658000	0.489779000
6	2.418319000	2.569663000	0.391321000
1	1.344565000	2.395947000	0.486410000
6	-2.497639000	-3.474573000	-0.887799000

1	-3.522168000	-3.749031000	-1.142413000
1	-1.944986000	-4.399153000	-0.713250000
1	-2.062568000	-2.978837000	-1.757371000
6	5.098711000	0.004107000	-0.475011000
1	6.136760000	0.007867000	-0.781522000
6	-3.042194000	-3.307822000	1.579816000
1	-2.975066000	-2.700123000	2.483911000
1	-2.511860000	-4.244194000	1.761099000
1	-4.094616000	-3.547351000	1.419694000
6	-3.020690000	3.285665000	1.607865000
1	-4.072441000	3.531407000	1.452840000
1	-2.485765000	4.217704000	1.797808000
1	-2.953913000	2.667979000	2.505180000
6	2.936792000	-3.197521000	1.695016000
1	2.417985000	-4.135959000	1.897883000
1	2.773698000	-2.530911000	2.543716000
1	4.005856000	-3.410588000	1.643246000
6	2.903119000	3.166009000	1.724642000
1	3.972575000	3.380643000	1.690150000
1	2.729400000	2.486265000	2.560706000
1	2.380568000	4.100715000	1.935040000
6	2.609512000	3.575139000	-0.753393000
1	2.025919000	4.476162000	-0.558200000
1	2.287892000	3.164126000	-1.711339000
1	3.651437000	3.881070000	-0.854591000
6	-2.481022000	3.476278000	-0.859117000
1	-2.051507000	2.987563000	-1.735460000
1	-1.922432000	4.395722000	-0.676583000
1	-3.504682000	3.759457000	-1.107541000
6	2.611769000	-3.567693000	-0.785524000
1	3.653952000	-3.864472000	-0.908963000
1	2.271452000	-3.143948000	-1.731401000
1	2.037313000	-4.475580000	-0.594938000
1	0.603892000	1.388861000	-2.463153000
1	0.922331000	-1.124011000	-2.499393000
1	-1.421386000	-0.141080000	-2.513314000
IDipp·$GeH_2BH_2^+$ (**1**$^+$)			
32	-0.000092000	0.087643000	2.037880000
7	-1.081491000	-0.051481000	-0.772356000
7	1.081570000	-0.051417000	-0.772266000
6	0.000009000	-0.012267000	0.034967000
6	-0.676873000	-0.119227000	-2.089247000
1	-1.383772000	-0.164800000	-2.897393000
6	-2.473381000	-0.062124000	-0.353695000
6	2.473480000	-0.061987000	-0.353659000
6	3.147632000	1.165027000	-0.239359000
6	0.677053000	-0.119190000	-2.089195000

1	1.384023000	-0.164608000	-2.897289000
6	-3.088189000	-1.305052000	-0.130421000
6	4.489221000	1.114676000	0.138282000
1	5.048674000	2.034570000	0.237693000
6	2.376836000	-2.637936000	-0.317409000
1	1.312256000	-2.443540000	-0.459316000
6	3.088410000	-1.304908000	-0.130581000
6	4.431398000	-1.286250000	0.243870000
1	4.944649000	-2.220553000	0.426589000
6	-3.147614000	1.164877000	-0.239428000
6	5.123180000	-0.093616000	0.380228000
1	6.165815000	-0.105787000	0.670539000
6	2.499670000	2.504858000	-0.561519000
1	1.416671000	2.366370000	-0.580511000
6	-4.431136000	-1.286463000	0.244241000
1	-4.944281000	-2.220792000	0.427106000
6	-4.489113000	1.114457000	0.138467000
1	-5.048635000	2.034303000	0.237901000
6	-2.376568000	-2.638053000	-0.317318000
1	-1.311982000	-2.443618000	-0.459113000
6	-2.499793000	2.504650000	-0.562095000
1	-1.416741000	2.366426000	-0.580224000
6	2.505896000	-3.551051000	0.910921000
1	3.538314000	-3.860656000	1.077978000
1	1.915102000	-4.456902000	0.765605000
1	2.154028000	-3.059250000	1.818822000
6	-5.122954000	-0.093872000	0.380635000
1	-6.165535000	-0.106058000	0.671141000
6	2.880768000	-3.352385000	-1.583410000
1	2.753779000	-2.735249000	-2.474610000
1	2.333443000	-4.284051000	-1.736546000
1	3.940816000	-3.597821000	-1.499716000
6	2.923522000	2.991906000	-1.958763000
1	4.001114000	3.159640000	-2.001365000
1	2.427417000	3.933543000	-2.200298000
1	2.670643000	2.268841000	-2.735888000
6	-2.880396000	-3.352348000	-1.583453000
1	-2.333100000	-4.284023000	-1.736640000
1	-2.753298000	-2.735126000	-2.474579000
1	-3.940467000	-3.597738000	-1.499883000
6	-2.922823000	2.990508000	-1.960017000
1	-4.000444000	3.157848000	-2.003451000
1	-2.669184000	2.266961000	-2.736441000
1	-2.426859000	3.932127000	-2.201909000
6	-2.799034000	3.580109000	0.491686000
1	-2.215723000	4.478410000	0.284241000
1	-2.552188000	3.242699000	1.499731000

1	-3.850254000	3.870359000	0.488910000
6	2.797912000	3.579526000	0.493356000
1	2.550121000	3.241331000	1.500908000
1	2.214804000	4.477996000	0.286066000
1	3.849148000	3.869732000	0.491827000
6	-2.505680000	-3.551345000	0.910868000
1	-3.538068000	-3.861157000	1.077730000
1	-2.154020000	-3.059616000	1.818890000
1	-1.914719000	-4.457082000	0.765515000
5	-0.001132000	2.015188000	2.781732000
1	-0.001476000	2.969961000	2.081550000
1	-0.001373000	2.135886000	3.961910000
1	-1.249982000	-0.702714000	2.444177000
1	1.250756000	-0.701194000	2.444181000

IDipp·GeH_2BH_2·NEt_3^+ (**2^+**)

32	0.243077000	0.706744000	-0.993439000
7	-0.196804000	-1.818126000	0.653003000
7	-2.013791000	-0.660018000	0.510287000
7	2.673329000	2.699194000	-0.874631000
6	-0.707123000	-0.666041000	0.158487000
6	-1.184116000	-2.533090000	1.301306000
1	-0.986156000	-3.483236000	1.761722000
6	1.179197000	-2.273839000	0.562243000
6	-2.995522000	0.370835000	0.226505000
6	3.676081000	3.335956000	0.068045000
1	4.193932000	2.512163000	0.552161000
1	3.089148000	3.826382000	0.839832000
6	-3.175751000	1.393513000	1.170306000
6	-2.320736000	-1.807222000	1.214478000
1	-3.311676000	-1.996013000	1.584755000
6	1.575403000	-2.990148000	-0.579531000
6	-4.147745000	2.352520000	0.886246000
1	-4.320795000	3.157911000	1.587262000
6	-3.596313000	-0.875168000	-1.944919000
1	-2.718652000	-1.458340000	-1.661611000
6	-3.754160000	0.265786000	-0.949698000
6	-4.710828000	1.254364000	-1.176008000
1	-5.316825000	1.212956000	-2.071143000
6	2.030145000	-2.035169000	1.655435000
6	-4.903704000	2.287768000	-0.272943000
1	-5.653599000	3.043068000	-0.470474000
6	-2.406537000	1.466353000	2.482604000
1	-1.601591000	0.730601000	2.451900000
6	1.836748000	3.759700000	-1.562707000
1	1.154062000	3.222683000	-2.221055000
1	2.507522000	4.331898000	-2.204374000
6	2.897648000	-3.431739000	-0.622438000

1	3.245515000	-3.986789000	-1.482683000
6	3.380151000	1.957181000	-1.992965000
1	3.875342000	2.706045000	-2.611906000
1	2.591905000	1.515634000	-2.601181000
6	3.338244000	-2.506605000	1.552285000
1	4.027720000	-2.343300000	2.369019000
6	0.612875000	-3.364342000	-1.698745000
1	-0.256600000	-2.708724000	-1.634517000
6	1.565902000	-1.363017000	2.941654000
1	0.626656000	-0.846477000	2.736201000
6	-3.355910000	-0.374005000	-3.376672000
1	-4.220372000	0.165572000	-3.766200000
1	-3.173593000	-1.219974000	-4.041758000
1	-2.493101000	0.291089000	-3.427824000
6	3.770378000	-3.188424000	0.426019000
1	4.790805000	-3.546015000	0.371317000
6	1.053112000	4.704749000	-0.668574000
1	0.340941000	4.181647000	-0.035461000
1	0.493909000	5.384005000	-1.314126000
1	1.696673000	5.317136000	-0.037778000
6	-4.807464000	-1.821837000	-1.892366000
1	-4.963138000	-2.223510000	-0.889529000
1	-4.661868000	-2.661711000	-2.574099000
1	-5.723186000	-1.306898000	-2.188171000
6	4.672074000	4.307854000	-0.547366000
1	5.313306000	3.841779000	-1.295081000
1	5.320787000	4.671605000	0.250937000
1	4.195930000	5.180134000	-0.995276000
6	-3.315289000	1.099270000	3.668336000
1	-4.133104000	1.814458000	3.773073000
1	-2.745872000	1.104000000	4.599547000
1	-3.756815000	0.108505000	3.547337000
6	0.115782000	-4.809852000	-1.513839000
1	-0.608680000	-5.064939000	-2.289450000
1	-0.363867000	-4.954216000	-0.544318000
1	0.944030000	-5.517627000	-1.581851000
6	4.377009000	0.885534000	-1.587469000
1	4.778953000	0.446471000	-2.502247000
1	3.919236000	0.089755000	-1.005433000
1	5.220731000	1.287237000	-1.027273000
6	1.290089000	-2.417106000	4.030061000
1	2.206328000	-2.947297000	4.296631000
1	0.561079000	-3.162042000	3.708072000
1	0.905834000	-1.938353000	4.932575000
6	2.549529000	-0.308180000	3.466105000
1	2.120231000	0.193630000	4.335059000
1	2.765545000	0.445042000	2.710015000

1	3.491551000	-0.755661000	3.787025000
6	-1.748793000	2.835891000	2.702933000
1	-1.083116000	3.094529000	1.878836000
1	-1.158251000	2.822930000	3.620635000
1	-2.489703000	3.630397000	2.802772000
6	1.210217000	-3.178197000	-3.099651000
1	2.021908000	-3.880724000	-3.293742000
1	1.594338000	-2.167558000	-3.241354000
1	0.443461000	-3.356730000	-3.855224000
5	1.777356000	1.698776000	0.048118000
1	1.260261000	2.344791000	0.915736000
1	2.505735000	0.865389000	0.498748000
1	0.605758000	-0.193375000	-2.203021000
1	-0.976505000	1.523274000	-1.477119000

IDipp·GeH_2BH_2·$NHEt_2^+$

32	0.072530000	0.801002000	-1.178314000
7	0.826048000	-1.328167000	0.851020000
7	-1.319995000	-1.136534000	0.699559000
7	0.907902000	3.779840000	-1.476415000
6	-0.154981000	-0.630143000	0.234153000
6	0.275317000	-2.273427000	1.693077000
1	0.885867000	-2.938411000	2.275252000
6	2.258229000	-1.149389000	0.686374000
6	-2.664982000	-0.727655000	0.337574000
1	1.515695000	4.483232000	-1.055418000
6	-3.295623000	0.253678000	1.117883000
6	-1.067584000	-2.152118000	1.600398000
1	-1.862024000	-2.688670000	2.085854000
6	2.902382000	-1.833847000	-0.358162000
6	-4.601907000	0.596698000	0.769723000
1	-5.126164000	1.346712000	1.346474000
6	-2.628760000	-2.479457000	-1.549975000
1	-1.580444000	-2.540485000	-1.253938000
6	-3.291997000	-1.375526000	-0.738300000
6	-4.597941000	-0.989064000	-1.036055000
1	-5.117859000	-1.462934000	-1.857772000
6	2.940609000	-0.354256000	1.622846000
6	-5.245414000	-0.013363000	-0.294993000
1	-6.260745000	0.267551000	-0.543979000
6	-2.640487000	0.905538000	2.327707000
1	-1.584200000	0.632575000	2.335017000
6	-0.447190000	4.426267000	-1.615112000
1	-1.181489000	3.623603000	-1.665924000
1	-0.467168000	4.957976000	-2.567570000
6	4.281392000	-1.660870000	-0.471254000
1	4.817754000	-2.167482000	-1.261648000
6	1.500353000	3.483602000	-2.826893000

1	1.404097000	4.390500000	-3.427055000
1	0.872926000	2.720227000	-3.288819000
6	4.319010000	-0.225680000	1.457016000
1	4.883640000	0.378504000	2.153527000
6	2.178392000	-2.792844000	-1.293853000
1	1.109549000	-2.580561000	-1.241020000
6	2.256698000	0.293571000	2.819996000
1	1.182250000	0.314453000	2.628577000
6	-2.651761000	-2.190032000	-3.058194000
1	-3.669011000	-2.182569000	-3.452410000
1	-2.100329000	-2.963843000	-3.595135000
1	-2.195323000	-1.226423000	-3.287489000
6	4.980934000	-0.863390000	0.420346000
1	6.052098000	-0.748382000	0.313736000
6	-0.752554000	5.388149000	-0.480486000
1	-0.743774000	4.891611000	0.487908000
1	-1.741312000	5.822011000	-0.632281000
1	-0.035409000	6.212453000	-0.457134000
6	-3.268010000	-3.844785000	-1.244576000
1	-3.217172000	-4.085684000	-0.181255000
1	-2.755253000	-4.635646000	-1.794990000
1	-4.319205000	-3.861761000	-1.537754000
6	-3.258713000	0.379930000	3.634694000
1	-4.316725000	0.639943000	3.700987000
1	-2.752204000	0.816505000	4.497409000
1	-3.180053000	-0.705878000	3.711161000
6	2.382455000	-4.247701000	-0.833170000
1	1.829807000	-4.932207000	-1.479369000
1	2.040165000	-4.401886000	0.191471000
1	3.437528000	-4.524487000	-0.875028000
6	2.952673000	3.047050000	-2.775164000
1	3.324449000	2.945829000	-3.795504000
1	3.080964000	2.094341000	-2.267028000
1	3.573608000	3.791066000	-2.271146000
6	2.487701000	-0.545263000	4.090589000
1	3.549248000	-0.581685000	4.342413000
1	2.141832000	-1.573408000	3.974454000
1	1.957298000	-0.105608000	4.937235000
6	2.696282000	1.744711000	3.057590000
1	2.101918000	2.180986000	3.862359000
1	2.560907000	2.353862000	2.165175000
1	3.742177000	1.806870000	3.361984000
6	-2.704312000	2.438419000	2.274115000
1	-2.263817000	2.821867000	1.353198000
1	-2.154384000	2.864372000	3.115050000
1	-3.730368000	2.803786000	2.336598000
6	2.592909000	-2.632610000	-2.762559000

1	3.627115000	-2.936775000	-2.929665000
1	2.483754000	-1.601671000	-3.100630000
1	1.966242000	-3.263715000	-3.394810000
5	0.941833000	2.555368000	-0.409458000
1	0.314512000	2.893494000	0.555481000
1	2.086587000	2.325310000	-0.144915000
1	0.857588000	-0.003866000	-2.242545000
1	-1.371682000	0.862366000	-1.726900000
IDipp·BH_2·NEt_3^+			
7	-1.134533000	-0.041608000	-1.012138000
7	1.022165000	-0.078685000	-1.064435000
7	0.072088000	-0.227058000	2.604028000
6	-0.034304000	0.083882000	-0.215144000
6	-0.764797000	-0.257087000	-2.322400000
1	-1.491052000	-0.365776000	-3.105812000
6	-2.538842000	0.045545000	-0.651826000
6	2.444939000	-0.024899000	-0.774711000
6	3.077691000	1.229451000	-0.746069000
6	0.582762000	-0.281337000	-2.354715000
1	1.266017000	-0.415356000	-3.172169000
6	-3.269171000	-1.151074000	-0.530946000
6	4.454507000	1.241753000	-0.523524000
1	4.977131000	2.187854000	-0.489459000
6	2.496636000	-2.604708000	-0.840092000
1	1.415213000	-2.483575000	-0.744974000
6	3.148894000	-1.237621000	-0.659408000
6	4.525055000	-1.157403000	-0.445729000
1	5.102886000	-2.066608000	-0.351132000
6	-3.133429000	1.315115000	-0.557866000
6	5.170351000	0.065920000	-0.367027000
1	6.239505000	0.102657000	-0.201648000
6	2.351412000	2.537923000	-1.025868000
1	1.279330000	2.356041000	-0.953964000
6	1.285030000	-1.136497000	2.603078000
1	1.266653000	-1.645514000	1.641522000
1	1.108661000	-1.892893000	3.370849000
6	-4.629310000	-1.040088000	-0.242938000
1	-5.226911000	-1.935883000	-0.141530000
6	-1.119745000	-1.172189000	2.650487000
1	-0.925807000	-1.885797000	3.454057000
1	-1.085006000	-1.727197000	1.714535000
6	-4.495683000	1.357725000	-0.261393000
1	-4.989515000	2.315772000	-0.174946000
6	-2.666922000	-2.530428000	-0.780768000
1	-1.578936000	-2.438266000	-0.763984000
6	-2.387154000	2.610982000	-0.844755000
1	-1.318096000	2.399623000	-0.834334000

6	2.934422000	-3.638208000	0.208570000
1	3.986462000	-3.904095000	0.099933000
1	2.357795000	-4.556446000	0.085637000
1	2.786652000	-3.283195000	1.228565000
6	-5.234701000	0.197371000	-0.096919000
1	-6.292034000	0.258021000	0.127012000
6	2.648225000	-0.511062000	2.841799000
1	2.872452000	0.293900000	2.147319000
1	3.396430000	-1.291165000	2.696664000
1	2.760744000	-0.149026000	3.861928000
6	2.777246000	-3.155772000	-2.250900000
1	2.441677000	-2.475012000	-3.033399000
1	2.268497000	-4.110695000	-2.395092000
1	3.846642000	-3.320426000	-2.394272000
6	2.645024000	3.015603000	-2.459583000
1	3.707157000	3.230125000	-2.591475000
1	2.089549000	3.930093000	-2.675569000
1	2.363404000	2.267430000	-3.202623000
6	-3.065669000	-3.048549000	-2.175260000
1	-2.590731000	-4.010958000	-2.374261000
1	-2.775475000	-2.359820000	-2.968917000
1	-4.146188000	-3.187847000	-2.240072000
6	-2.496730000	-0.558182000	2.853381000
1	-3.239661000	-1.328149000	2.645713000
1	-2.689882000	0.273862000	2.182215000
1	-2.661382000	-0.233011000	3.879204000
6	-2.742139000	3.131484000	-2.249522000
1	-3.803308000	3.377401000	-2.320143000
1	-2.520570000	2.394856000	-3.023775000
1	-2.173564000	4.036059000	-2.473156000
6	-2.638590000	3.695490000	0.211529000
1	-2.009270000	4.563439000	0.007853000
1	-2.405780000	3.338325000	1.214925000
1	-3.673705000	4.039850000	0.204455000
6	2.685516000	3.639468000	-0.010872000
1	2.496474000	3.311480000	1.011422000
1	2.068563000	4.518957000	-0.202550000
1	3.727217000	3.956130000	-0.079990000
6	-3.051663000	-3.570925000	0.281382000
1	-4.117870000	-3.799197000	0.257436000
1	-2.805962000	-3.242105000	1.291018000
1	-2.519305000	-4.504903000	0.093960000
5	0.031706000	0.741298000	1.284582000
1	1.036808000	1.383126000	1.306644000
1	-0.931275000	1.440132000	1.410496000
6	0.043644000	0.610820000	3.877528000
1	-0.998361000	0.843751000	4.063285000

1	0.375038000	-0.051600000	4.680740000
6	0.811751000	1.925129000	3.907895000
1	0.388904000	2.650644000	3.217501000
1	1.871428000	1.826423000	3.696614000
1	0.710248000	2.327750000	4.918167000
IDipp · BH_2 · $NHEt_2^+$ (**3a**$^+$)			
7	1.079933000	-0.177867000	-0.834594000
7	-1.079539000	-0.177902000	-0.834696000
7	-0.000688000	0.816655000	2.619062000
6	0.000132000	-0.151931000	-0.004757000
6	0.675218000	-0.240630000	-2.150598000
1	1.379503000	-0.288710000	-2.959794000
6	2.487543000	-0.187436000	-0.474133000
6	-2.487169000	-0.187812000	-0.474292000
1	-0.000901000	0.348891000	3.525808000
6	-3.088052000	-1.415911000	-0.150321000
6	-0.674702000	-0.240656000	-2.150689000
1	-1.378904000	-0.288748000	-2.959947000
6	3.208918000	1.017740000	-0.559975000
6	-4.449610000	-1.397146000	0.151930000
1	-4.948005000	-2.321329000	0.410336000
6	-2.596458000	2.326620000	-1.047541000
1	-1.510436000	2.251955000	-0.953301000
6	-3.209009000	1.017050000	-0.560751000
6	-4.567945000	0.967753000	-0.250362000
1	-5.159119000	1.871542000	-0.303093000
6	3.088921000	-1.415483000	-0.150859000
6	-5.180560000	-0.221227000	0.111355000
1	-6.237075000	-0.233884000	0.346667000
6	-2.346189000	-2.744711000	-0.193555000
1	-1.276218000	-2.538481000	-0.216958000
6	-1.244448000	1.662492000	2.595070000
1	-1.425860000	1.934859000	1.558344000
1	-1.027520000	2.579862000	3.144449000
6	4.567929000	0.968769000	-0.249833000
1	5.158725000	1.872830000	-0.302059000
6	1.242538000	1.663269000	2.596095000
1	1.024927000	2.579916000	3.146411000
1	1.423803000	1.936890000	1.559642000
6	4.450537000	-1.396426000	0.151028000
1	4.949350000	-2.320578000	0.408759000
6	2.595809000	2.327343000	-1.045921000
1	1.509847000	2.252374000	-0.951190000
6	2.347387000	-2.744480000	-0.194325000
1	1.277362000	-2.538454000	-0.217973000
6	-3.043898000	3.553011000	-0.237542000
1	-4.097725000	3.781678000	-0.400237000

1	-2.474793000	4.429970000	-0.550114000
1	-2.897357000	3.421173000	0.834743000
6	5.181059000	-0.220206000	0.110992000
1	6.237635000	-0.232644000	0.346050000
6	-2.448680000	0.971819000	3.210063000
1	-2.746004000	0.085132000	2.656164000
1	-3.290360000	1.664773000	3.217376000
1	-2.251991000	0.688564000	4.247128000
6	-2.916950000	2.556297000	-2.536775000
1	-2.570869000	1.736098000	-3.165581000
1	-2.442915000	3.473643000	-2.890593000
1	-3.993482000	2.655187000	-2.686854000
6	-2.696813000	-3.509806000	-1.482628000
1	-3.758734000	-3.760550000	-1.512817000
1	-2.131162000	-4.441785000	-1.535695000
1	-2.468606000	-2.925874000	-2.376050000
6	2.915528000	2.557735000	-2.535215000
1	2.441258000	3.475219000	-2.888363000
1	2.569177000	1.737819000	-3.164222000
1	3.991982000	2.656768000	-2.685764000
6	2.447217000	0.972858000	3.210462000
1	3.288041000	1.666833000	3.219576000
1	2.745928000	0.087543000	2.655156000
1	2.250288000	0.687433000	4.246891000
6	2.698431000	-3.509496000	-1.483295000
1	3.760403000	-3.760065000	-1.513283000
1	2.470312000	-2.925598000	-2.376764000
1	2.132974000	-4.441589000	-1.536516000
6	2.606104000	-3.617909000	1.040677000
1	1.981450000	-4.511667000	0.998895000
1	2.371894000	-3.086330000	1.963198000
1	3.643112000	-3.952469000	1.093577000
6	-2.604996000	-3.618289000	1.041325000
1	-2.371271000	-3.086695000	1.963964000
1	-1.980002000	-4.511809000	0.999677000
1	-3.641897000	-3.953244000	1.093869000
6	3.043362000	3.553506000	-0.235626000
1	4.096929000	3.782787000	-0.399149000
1	2.897887000	3.420973000	0.836724000
1	2.473548000	4.430363000	-0.547192000
5	-0.000031000	-0.449219000	1.593631000
1	-0.985385000	-1.075801000	1.851533000
1	0.985558000	-1.075125000	1.851987000

References

(1) Oxford Diffraction /Agilent Technologies UK Ltd, Yarnton, England. CrysAlis PRO **2014**.
(2) Dolomanov, O. V.; Bourhis, L. J.; Gildea, R. J.; Howard, J. A. K.; Puschmann, H. OLEX2 : a complete structure solution, refinement and analysis program. *J. Appl. Cryst.* **2009**, *42* (2), 339–341. DOI: 10.1107/S0021889808042726.
(3) Sheldrick, G. M. SHELXT - integrated space-group and crystal-structure determination. *Acta Cryst.* **2015**, *71A* (Pt 1), 3–8. DOI: 10.1107/S2053273314026370.
(4) Sheldrick, G. M. Crystal structure refinement with SHELXL. *Acta Cryst.* **2015**, *71C* (Pt 1), 3–8. DOI: 10.1107/S2053229614024218.
